# Novel Virus Identification through Metagenomics: A Systematic Review

**DOI:** 10.3390/life12122048

**Published:** 2022-12-07

**Authors:** Cristian Bassi, Paola Guerriero, Marina Pierantoni, Elisa Callegari, Silvia Sabbioni

**Affiliations:** 1 Department of Translational Medicine, University of Ferrara, 44121 Ferrara, Italy; 2 Laboratorio per Le Tecnologie delle Terapie Avanzate (LTTA), University of Ferrara, 44121 Ferrara, Italy; 3 Department of Life Science and Biotechnology, University of Ferrara, 44121 Ferrara, Italy

**Keywords:** viral metagenomics, NGS, novel virus, plant, animal, environment

## Abstract

Metagenomic Next Generation Sequencing (mNGS) allows the evaluation of complex microbial communities, avoiding isolation and cultivation of each microbial species, and does not require prior knowledge of the microbial sequences present in the sample. Applications of mNGS include virome characterization, new virus discovery and full-length viral genome reconstruction, either from virus preparations enriched in culture or directly from clinical and environmental specimens. Here, we systematically reviewed studies that describe novel virus identification through mNGS from samples of different origin (plant, animal and environment). Without imposing time limits to the search, 379 publications were identified that met the search parameters. Sample types, geographical origin, enrichment and nucleic acid extraction methods, sequencing platforms, bioinformatic analytical steps and identified viral families were described. The review highlights mNGS as a feasible method for novel virus discovery from samples of different origins, describes which kind of heterogeneous experimental and analytical protocols are currently used and provides useful information such as the different commercial kits used for the purification of nucleic acids and bioinformatics analytical pipelines.

## 1. Introduction

Viruses are the most abundant organisms on Earth [1] and play a key role in the ecosystem in which they reside [2]. Virus interactions affect their own abundance and evolution [3]; furthermore they have a deep impact on host individuals, populations and communities [4,5], as well as on environment biogeochemical cycles [6]. 

Next generation sequencing (NGS) technologies make it possible to retrieve, directly and quickly, millions of RNA or DNA sequences from different types of samples, such as environmental [7,8,9], animal/clinical [10,11] and vegetal [12,13,14,15,16]. In particular, the shotgun metagenomic sequencing (mNGS) has allowed the development of methods of analysis of complex microbial communities and the discovery of novel viruses, greatly expanding our knowledge of the Earth’s virome [17,18,19].

Before mNGS approach development, virus discovery was challenging due to highly variable genomes: the lack of shared sequences hindered the employment of an amplicon-based strategy [20], which can be exploited for bacteria analysis through 16S rRNA gene sequencing [21]. Shotgun mNGS overcame this challenge, providing the untargeted sequencing of all the microbial genomes present in the sample; then, the found reads could be classified based on their similarity to the reference genomes [9,20].

In the last years, metagenomics applications have expanded into several field. mNGS moved from research to clinical laboratories, modifying the approach for infectious disease diagnosis and treatment, as well as improving cancer-associated viruses analysis [10]. Furthermore, mNGS revolutionized virome ecology studies, which previously analyzed viruses individually, rather than as a whole, hindering the discovery of their multiplicity as well the breakthrough of their possible interactions [3].

Contribution of mNGS was decisive for SARS-CoV-2 identification [22,23] as well as for monitoring its evolution and spread patterns [24,25]. mNGS technology is also critical to develop multidisciplinary programs and policies to support animal and environmental health, focusing on food safety, antimicrobial resistance, control of zoonotic disease and the study of neglected tropical diseases, which are the main goals of the “One Health approach” (https://www.who.int/europe/initiatives/one-health, accessed on 28 June 2022). This term was used for the first time in 2003–2004 to underline the interconnection between human, animal and environment health after the Severe acute Respiratory Disease (SARS) and avian influenza H5N1 worldwide transmission [26]. 

Notwithstanding the progress made, virus analysis exploiting mNGS is still complex because of several critical issues. Sample collection and storage affect both the quality and accuracy of metagenomic data: validated procedures for a specific type of sample might be not suitable for others [27]. Furthermore, the enrichment process and nucleic acid extraction techniques should be carefully selected, since they might impact the amount of certain microbial species in the sample, causing an overestimation of the most abundant species or of those microorganisms that can be more easily lysed [28]. Another critical point concerns the sequence assembly; indeed, choosing the most suitable assembly software is essential to identify not only the most represented microorganism in the sample but also the less abundant ones, which is a typical condition of viruses [29,30,31,32].

Assessing the current state of the art for mNGS applications is relevant to develop strategies and resources to support the application of NGS more broadly, contributing to the design of effective disease control and prevention strategies [33].The main objectives of this critical review are to provide an overview of (1) the published literature on novel virus identification through mNGS in environmental, animal and plant samples, (2) different nucleic acid purification and sequencing platforms and (3) the critical steps in data analysis process, highlighting the bio-informatic tools available. 

## 2. Material and Methods

### 2.1. Search Strategy and Selection Criteria

We conducted a search of all eligible studies in the MEDLINE electronic database on 30 November 2021, without imposing time limits to the search, to provide an overview of studies that performed mNGS to identify new viruses from samples of various origins (plants, animals and the environment). The following search string was used: (“environmental genomics” [mesh] OR ecogenomics [tiab] or “community genomics” [tiab] OR metagenomics [tiab] or “environmental genomics” [tiab] or ecogenomics [tiab] or community genomics [tiab]) AND (“viruses” [tiab] OR “viruses” [MeSH Terms] OR “virus’s” [tiab] OR “viruses” [tiab] OR “virus” [tiab])) AND (Novel [tiab] OR new* [tiab] OR original [tiab] OR unknown [tiab]). The identified references were imported on EndNote [34]. Two independent reviewers (M.P. and P.G.) screened the titles and abstracts of all unique references. Full texts of remaining articles were assessed for eligibility, after a first screening. Exclusion criteria were use of languages other than English, articles describing known viruses, articles not performing NGS and articles that did not report original data (i.e., review papers, editorial, and commentaries). Only studies that described novel viruses identified by mNGS in specimens from animals, the environment or plants were included. The workflow of the systematic review was reported following the guidelines of the Preferred Reporting Items for Systematic Reviews and Meta-Analyses (PRISMA) [35].

### 2.2. Data Extraction and Analysis

Two authors (M.P. and P.G.) performed data extraction independently. The following information was extracted from each included study: year of publication, journal name, first author, type of sample (environmental, vegetal, animal), geographical origin of the sample, sample size, enrichment method, nucleic acid extraction kits, nucleic acid extraction method, sequencing platform, de novo assembly (if any), viral genome type (RNA or DNA), viral family detected, virus detected. The information on taxonomy of different virus families was retrieved through the Taxonomy Browser [36].

Enrichment methods were stratified in nine groups: pore filtration, tangential flow filtration, ultracentrifugation, polyethylene glycol (PEG) precipitation, FeCl3 precipitation, ultrafiltration, chemical flocculation, fluidic circuit and syringe filtration. Nucleic acid extraction strategies were stratified in four groups: type of nucleic acid (DNA only, RNA only, both DNA and RNA), manufacturer, extraction method (column-based, solvent-based, magnetic bead–based, magnetic glass particle–based, silica membrane–based, filter tube, other/multiple) and type of sample actually used with that specific method. Sample size was stratified in four groups: ≤10, 11–100, 101–500 and >500. 

The sequencing platforms were divided into 4 primary sequencing platforms: Ion Torrent (Thermo Fisher, Waltham, MA, USA), Illumina (San Diego, CA, USA), Sanger (Hinxton, UK), 454 pyrosequencing (Roche, Basel, Switzerland).

## 3. Results

### 3.1. Literature Search and General Characteristics of the Included Studies

**Figure 1:**
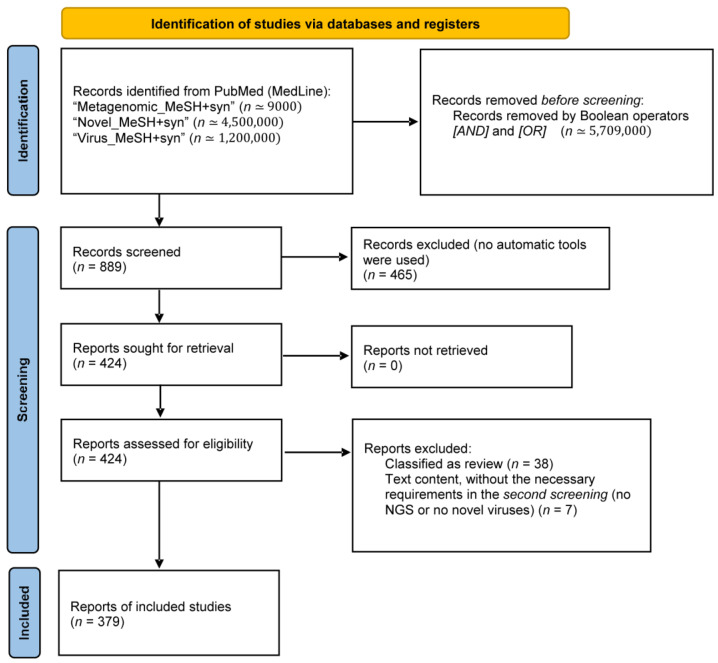
**Flowchart of the literature search.** Search was performed following the guidelines of the preferred reporting items for systematic reviews and meta-analyses (PRISMA).

A total of 889 records were identified, and 465 were excluded after the first screening by title and abstract analysis, since the identified virus was already known. From the remaining 424 eligible records, 38 non-original articles (reviews) and 7 articles (no NGS or no novel viruses) were excluded, leaving 379 studies included in data extraction and analysis (Figure 1). The list of the 379 references is provided in Table S2.

**Figure 2:**
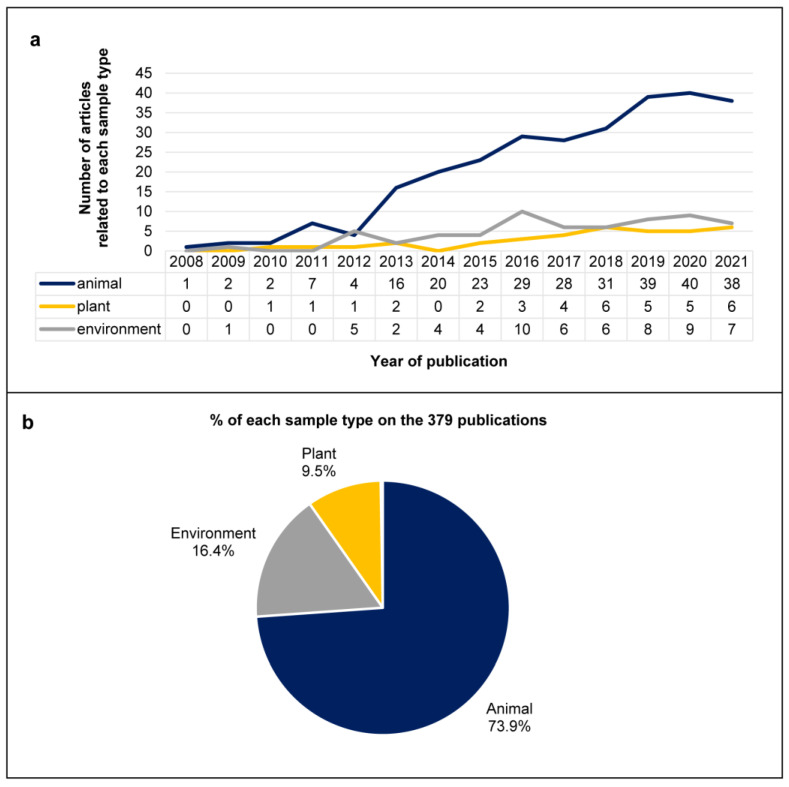
**Summary of the publications included in the study per year and per type of sample**. (**a**) Trend for annual publication: each column represents the number of articles by year of publication, from 2008 to 2021, divided by type of sample (environment, animal, plant). (**b**) Percentage of each sample type of the 379 publications: animal (dark blue), environment (light blue), yellow (plant).

The earliest included article was published in 2008, with a gradual increase in subsequent years, reaching an apparent plateau with up to 54 elements in 2020. After 2013, there was a sharp increase in the number of articles concerning animal viruses, which accounted for 73.9% of the total, compared to 9.5% and 16.4% for viruses identified in plants and environmental samples, respectively (Figure 2a,b).

**Figure 3:**
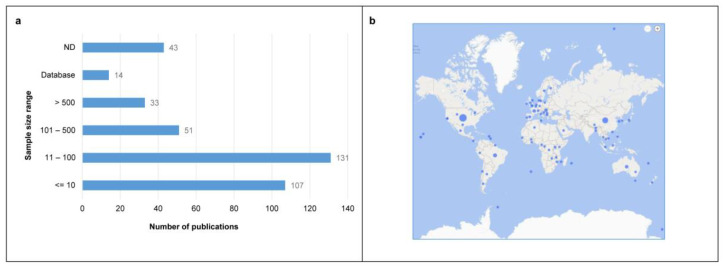
**Sample size and geographical provenience.** (**a**) Number of publications for each sample size range; X axes: number of publications, Y axes; sample size range. ND: sample size not specified. (**b**) Map showing the geographical provenance of the samples; dots indicate the samples/publications (dot dimension reflects different publication numbers). (**b**) was generated with Bing Maps add-on for Microsoft Office Excel.

The number of samples analyzed in the published literature was highly variable, between 1 and over 200,000 samples (mosquito specimens [37]). The most frequent sample size was between 11–100 (published papers *n* = 131). A total of 107 papers showed a sample size ≤10, 51 articles analyzed a number of samples between 101–500, and 43 analyzed >500 samples. In addition, 43 papers did not state the sample size, and 14 papers analyzed sample sets already present in databases (Figure 3a).

From a geographical point of view, the studies were carried out in all 6 World Health Organization (WHO) regions, with 36% in the Americas Region (*n* = 123), 29% in the South-East Asia Region (*n* = 99) and 26% in the European Region (*n* = 90), followed by the Africa Region (6%, *n* = 21), Western Pacific Region (2%, *n* = 6) and Eastern Mediterranean Region (*n* = 1) (Figure 3b). A total of 30 studies did not indicate the exact origin of the samples, while those taken from the Arctic (*n* = 2), Antarctic (*n* = 3) and oceans (*n* = 8) are not included in the WHO classification.

### 3.2. Enrichment Strategies, Nucleic Acid Purificationand Sequencing Platforms

**Figure 4:**
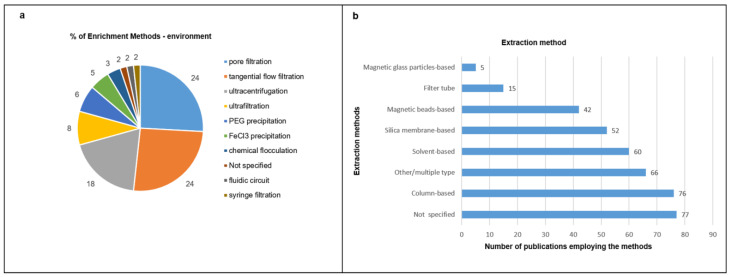
**Sample enrichment and purification.** (**a**) Enrichment methods used for environmental samples prior to extraction. The figure shows the different enrichment methods and the percentage of each method of the total number of publications (environmental sample). (**b**) Extraction method. The graph shows the different extraction methods used for all samples. X axis: publication number; Y axis: extraction method.

Limited to the environmental samples, several viral enrichment strategies were employed, with the pore filtration and tangential flow filtration method (24%, *n* = 15 each) being the most popular; other methods included ultracentrifugation (18%, *n* = 11), ultrafiltration (8%, *n* = 5), PEG precipitation (6%, *n* = 4), FeCl3 precipitation (5%, *n* = 3), chemical flocculation (3%, *n* = 2), fluidic circuit (2%, *n* = 1) and syringe filtration (2%, *n* = 1). One report (2%) did not indicate sample enrichment (Figure 4a). Some articles used more than one enrichment method: ultracentrifugation and tangential flow filtration [38,39]; ultrafiltration and ultracentrifugation [40,41,42]; tangential flow filtration and PEG precipitation [43].

For extraction methods, commercial kits were employed. These kits allowed extraction of both DNA and RNA (manufacturer *n* = 16: Beckman Coulter, Brea, CA, USA; Biomérieux, Marcy l’Etoile, France; Biosearch Technologies, Hoddesdon, UK; Intron Biotechnology, Sagimakgol-ro, Jungwon-gu Seongnam, Gyeonggi, Republic Of Korea; Invitrogen, Waltham, MA, USA; Life Technologies, Carlsbad, CA, USA; Macherey-Nagel, Düren, Germany; Omega Biotek, Norcross, GA, USA; Perkin Elmer, Waltham, MA, USA; Promega, Madison, WI, USA; Qiagen, Hilden, Germany; Roche; Sigma-Aldrich, St. Louis, MO, USA; TaKaRa, Kusatsu, Japan; ThermoFisher; Zymo Research, Irvine, CA, USA), DNA only (manufacturer *n* = 12: GE Healthcare, Chicago, IL, USA; Invitrogen; Macherey–Nagel; Mo Bio, Carlsbad, CA, USA; MP Biomedicals, Irvine, CA, USA; PacBio, Menlo Park, CA, USA; Promega; Qiagen; Roche; Sigma-Aldrich; TaKaRa; ThermoFisher) or RNA only (manufacturer *n* = 12: Ambion, Foster City, CA, USA;. Life Technologies; Macherey-Nagel; Mo Bio; NEB, Ipswich, MA, USA; NipponGene, Tokyo, Japan; NZYTech, Lisboa, Portugal; Qiagen; Roche; Sigma-Aldrich; ThermoFisher; Zymo Research).

Commercial kits are based on different extraction methods. The most employed are column-based (19%, *n* = 76), followed by solvent-based (15%, *n* = 60), silica membrane–based (13%; *n* = 52), magnetic bead–based (11%, *n* = 42), filter tube–based (4%, *n* = 15) and magnetic glass particle–based (1%, *n* = 5); 66 (17%) reports indicated other/multiple methods, and 77 reports (20%) did not specify the method used (Figure 4b).

The most employed commercial kit was QIAamp viral RNA mini kit (Qiagen) [25,44,45,46,47,48,49,50,51,52,53,54,55,56,57,58,59,60,61,62,63,64,65,66,67,68,69,70,71,72,73,74,75,76,77,78,79,80,81,82,83,84,85,86,87,88,89,90,91,92,93,94,95,96], followed by MagMAX Viral RNA Isolation kit (ThermoFisher) [37,60,97,98,99,100,101,102,103,104,105,106,107,108,109,110,111,112,113,114,115,116,117], QIAamp MinElute Virus Spin Kit (Qiagen) [118,119,120,121,122,123,124,125,126,127,128,129,130,131,132,133,134,135,136,137,138,139], High Pure Viral Nucleic Acid kit (Roche) [140,141,142,143,144,145,146,147,148,149,150], QIAamp viral RNA kit (Qiagen) [38,151,152,153,154,155,156,157,158], QIAamp DNA Mini Kit (Qiagen) [25,43,159,160,161,162,163], RNeasy Mini Kit (Qiagen) [159,160,161,164,165,166,167], DNeasy Blood & Tissue Kit (Qiagen) [39,168,169,170,171,172] and RNeasy Plus Mini Kit (Qiagen) [118,173,174,175,176,177]. Supplementary Table S1 shows the complete list of commercial kits employed and specimens from which the nucleic acid (DNA, RNA, or both) was extracted (Table S1). Notably, DNA purification kits also allow RNA purification, which is identified as long as a retrotranscription step is introduced before library preparation [171,178].

**Figure 5:**
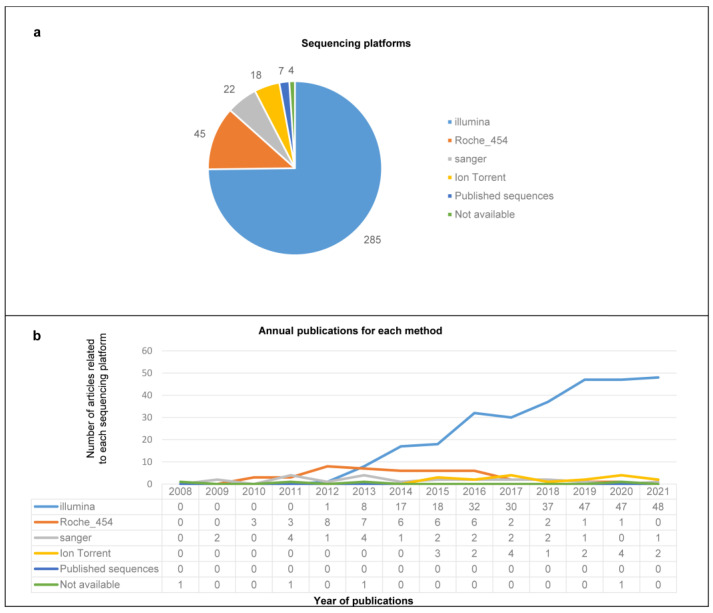
**Sequencing platforms.** (**a**) The number of publications employing the different platforms. (**b**) Annual publications for each sequencing platform. Each column represents the number of articles by year of publication, from 2008 to 2021, divided by platform. “Published sequences” refers to analysis of previously published sequences.

Regarding the sequencing platforms, the studies were carried out using four sequencing methods: Illumina, Sanger, Ion Torrent and Roche 454. The most utilized platform was Illumina (75%, *n* = 285), followed by Roche 454 (12%, *n* = 45), Sanger sequencing (6%, *n* = 22), and Ion Torrent (5%: *n* = 18). Seven records (2%) included analysis of public viral metagenomes deposited in databases (as NCBI RefSeq, short read archive (SRA) database, ICTV database) [179,180,181,182,183,184,185], and four records did not specify which sequencing platforms were used [124,128,129,186] (Figure 5a). Illumina has become the most used platform since 2013 (Figure 5b).

Lastly, for the identification of viral genomes, 62% of the articles (*n* = 234) used a strategy based on overlapping reads and generations of “contigs”. This strategy, named de novo assembly, allows the reconstruction of the original genome, starting from the sequenced fragments. Among the 234 articles, 77% concerned samples of animal origin, 12% samples of environmental origin, 10% plant origin. A progressive increase in de novo assembly is observed from 2016 until 2019 (Figure S1).

### 3.3. Overview of the Extracted Characteristics

For each included article, it was then possible to collect and present data regarding first author, date of publication, host, sample size and provenance, type of specimen, enrichment strategies, nucleic acid purification kits, retro-transcription, sequencing platforms, de novo assembly, genome, viral family and name of the novel virus identified (Table S2).

**Figure 6:**
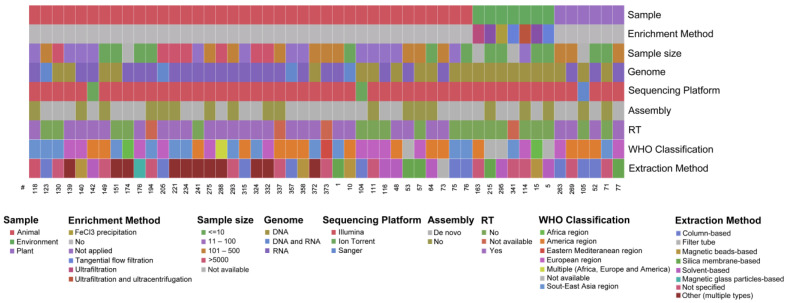
**An overview of 51 included publications (year 2021).** Each column represents one paper, and numbers refer to the list of references provided in Supplementary File S1. Each row describes one specific characteristic (from top to bottom): sample, enrichment method, sample size, viral genome, sequencing platform, de novo assembly, retro-transcription (RT), provenance of the sample (WHO classification) and extraction method. Categories and color codes for each data field are indicated in the figure legend. Gray boxes indicate that the technique was not performed or the information was not provided.

By way of example, a graphical overview of the main characteristics extracted from articles published in 2021 is provided (Figure 6). Each paper is cited in the x axis (numbers refer to the list of references provided in Table S2) and is represented by a column describing its characteristics. By means of color coding, it is possible to identify sample type, origin and size; the method used for enrichment, purification, sequencing and analysis; and the viral genome.

### 3.4. Viral Genomes and Viral Families in Different Sample Types

**Figure 7:**
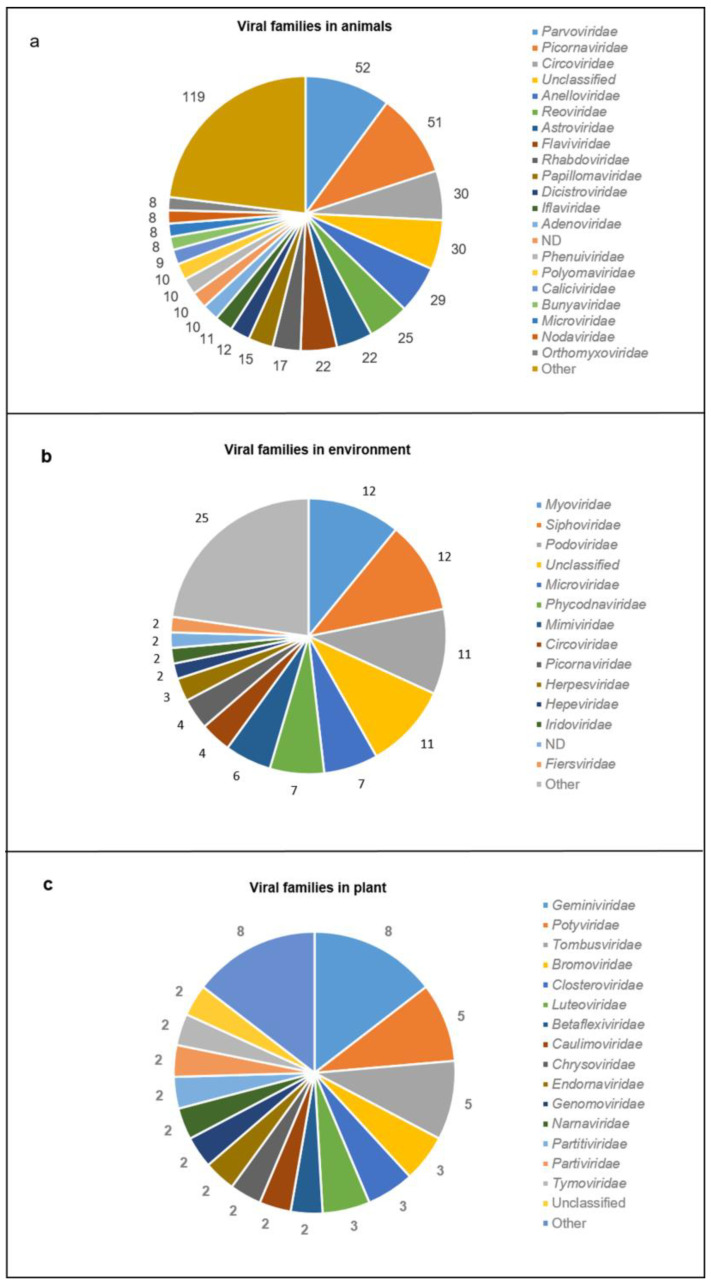
**Viral families discovered in different sample types.** The figure shows the viral families and the number of new viruses belonging to each family in (**a**) animal samples; “other”: families that have been described in less than eight reports; ND: viruses not belonging to known families; (**b**) environmental samples; “other”: families that have been described in a single report; ND: viruses not belonging to known families; (**c**) plant samples; “other”: families that have been described in a single report. For all sample types, “unclassified”: as defined in NCBI taxonomy and viral zone databases.

Among the articles describing novel viruses in animal samples, 270 viruses were identified as belonging to known viral families. In particular *Parvoviridae*, *Picornaviridae*, *Circoviridae*, *Anelloviridae*, *Reoviridae*, *Astroviridae*, *Flaviviridae*, *Rhabdoviridae*, *Papillomaviridae* and *Dicistroviridae*, are the most represented families (Figure 7a); for 10 publications, it was not possible to identify the family to which they belong [125,130,180,181,187,188,189,190,191,192]. Among the articles describing novel viruses in the environment, 60 viruses were identified as belonging to known viral families; in particular, *Siphoviridae*, *Myoviridae*, *Podoviridae*, *Microviridae*, *Phycodnaviridae*, *Mimiviridae*, *Picornaviridae*, *Circoviridae*, *Herpesviridae* and *Hepeviridae* are the most represented (Figure 7b); for two publications it was not possible to identify the family to which they belong [193,194]. Among the articles describing novel viruses in plants, all 36 articles described novel viruses belonging to known viral families, with *Geminiviridae*, *Tombusviridae*, *Potyviridae*, *Luteoviridae*, *Bromoviridae*, *Closteroviridae*, Unclassified, *Genomoviridae*, *Partitiviridae*, *Tymoviridae* and *Narnaviridae* being the most represented families (Figure 7c).

**Figure 8:**
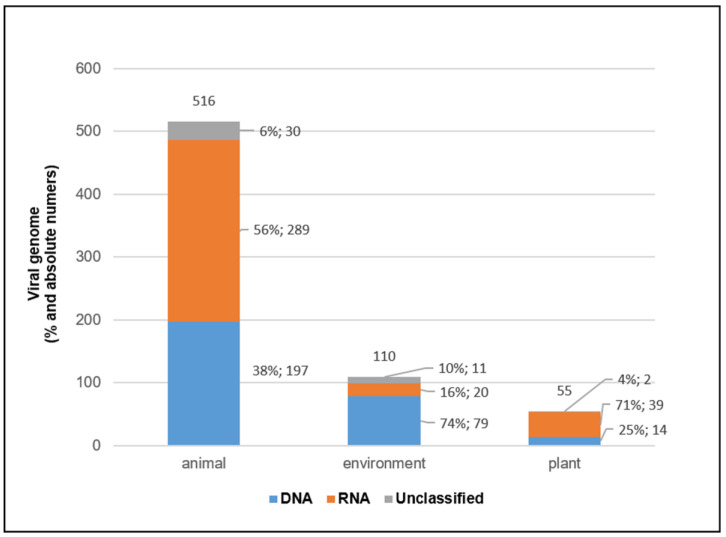
**Viral Genomes**. The graph shows viral genomes of the identified viruses: DNA (blue bar), RNA (orange bar) and unclassified (gray bar) identified in the three sample types (animal, environment, plant). The number of viruses (percentage and absolute number) for each genome type is indicated on the side of the bars. X axis: DNA or RNA genomes in different sample types; y-axis: viral genomes.

In total, 681 different novel viruses were identified, 290 with DNA genome, 348 with RNA genome and 43 unclassified, as defined in NCBI taxonomy and viral zone databases. In particular, 516 novel viruses were identified in animal samples, 110 in environmental samples and 55 in plants. Analysis performed by type of sample revealed that the distribution of classified viral genomes in the different types of samples was not homogeneous, with a prevalence of RNA viruses compared to DNA viruses in plants (RNA 71% vs. DNA 25%) and animals (RNA 56% vs. DNA 38%), while in environmental samples, a prevalence of DNA viruses over RNA viruses (DNA 74% vs. RNA 16%) was observed (Figure 8).

### 3.5. Novel Viruses Found by mNGS Studies

Among viruses infecting animals, new bacteriophages were identified [195]. Bacteriophages are implicated in the dynamics and diversity of bacterial populations in a number of ecosystems, including the human gut [196,197,198], confirming that mNGS technologies allow investigation of the so-called “viral dark matter” [199].

Potential pathogenic viruses for species at zoonotic risk to humans were also identified. For example, new *Bocaparvoviruses* were identified in different animal species, such as alpacas [200], wild squirrel [124] and tufted deer [121]. Novel *Bocaparvoviruses* were identified in different geographical areas and in different animal species, including bats [201], camels [202], gorillas [203], marmots [204], pigs [205] and rodents [206], and are associated with various veterinary diseases of the respiratory and gastrointestinal tract and acute respiratory diseases in humans. Their presence in previously unreported animal species, such as the alpaca, whose close contact with humans is favored by breeding and exposure in geographical areas other than that of origin, may be an important element in relation to possible zoonotic risks. 

Among the viruses that infect plants, a new *Grablovirus* was identified in *Prunus *spp., confirming the possible use of mNGS in the diagnosis of viral infections in economically important sectors such as agriculture. The appearance of these viruses in temperate and tropical woody plant species and herbaceous plants is symptomatic of climate change consequences, since a common feature of viruses within the *Geminiviridae* family was that they were primarily pathogens of economically important plant species, mainly in the tropical and subtropical regions of the world [207]. Other viruses belonging to the same family *Geminiviridae* are the *Begomoviruses*, which infect dicotyledonous plants and have an economic effect linked to the cultivation of tomatoes. A new *Begomovirus* associated with severe symptoms in tomatoes was identified in Brazil, where the preferential strategy for *Begomovirus* management in tomatoes is the employment of cultivars carrying disease resistance/tolerance genes, such as the Ty-1 gene [208]. The new *Begomovirus* identified in this article suggests a mechanism of potential adaptation to the tolerance factor Ty-1, which highlights the potential drawbacks of employing virus-specific resistance in tomato breeding [209].

Among the environmental viruses, an example is the identification of a new *Phycodnavirus* belonging to the *Phycodnaviridae* family in a phytoplankton bloom occurring in the West Antarctic Peninsula (WAP) [210]. The identification of this new virus suggests the usefulness of mNGS in the study and monitoring of plankton dynamics as responses to climate change in this warming region [211]. Another study identifies a new virus belonging to the *Picobirnaviridae* family in the wastewater from Santiago de Chile, confirming the relevance of sewage viromes as epidemiological surveillance tools and supporting the usefulness of sewage viral metagenomics for public health surveillance [40]. 

Some examples of new viruses identified in different sample types are described in Table 1.

### 3.6. Bioinformatics Pipelines

The various published articles show different algorithms for the metagenomic analysis or different reference databases, but present four common steps in the bioinformatics analysis process: (a) quality control (QC) check, (b) read trimming, adapter removal, and further filtering, (c) viral genome identification and (d) analysis of the results [19].

#### 3.6.1. Quality Control (QC) Check, Sequence Trimming and Filtering

Quality control (QC) is a critical step in the processing of NGS data and aims to produce high quality data, starting from the raw sequences generated by the sequencing platform, to be provided to the algorithms involved in the subsequent analysis phases (raw-read processing). The programs that carry out this phase are responsible for performing the quality control of the obtained data, the removal of the sequences of the adapters and indexes (sequences artificially inserted in the reads of each sample, in order to recognize the fragments belonging to each sample), the filtering of low quality sequences, and in some cases the filtering of polyclonal sequences. The QC protocol must be designed for a specific dataset, taking into account the differences inherent in different sequencing technologies (short-read platform vs. long-read platform) [229].

The list of the most commonly used programs/algorithms for these phases is provided in Table 2.

#### 3.6.2. Viral Genome Identification

The next step involves the removal of high quality reads belonging to the host genome, through alignment to the host reference genome, using common alignment algorithms (for example Bowtie2 [236], STAR [237], Blast [238], BWA [239] and SAMtools [240]). High quality reads, filtered by the host genome, can now be used for identification of viral genomes through two different strategies: (1) by alignment of reads to a reference sequence database (normally the NCBI nucleotide database (nt) [241], the non-redundant protein database (nr) [241] or the Reference Viral Database (RVDB) [242]); (2) by new de-novo assembly, based on overlapping reads rather than mapping reads, to reference genomes. 

In the first case, since amino acid sequences are more conserved than nucleotide sequences, the alignment of the reads to a protein database instead of a nucleotide database, improves the sensitivity of the classification, However, it requires more computational power and more computational time. De novo assembly instead allows the reconstruction of the original genome, starting from the sequenced fragments (generally in the range from 100 to 250 bp), through the production of longer assembled sequences, called “contigs” and “scaffolds”. Metagenomic assembly is a very complex process and requires a high uniformity of coverage throughout the genome, and also between different genomes, in case there are more viruses in the sample [243]. The most used tools for this process are listed in Table 3.

The quality and completeness of the final assembly can be evaluated by parameters such as N50 (which summarizes assembly contiguity in a single number: half the genome is assembled on contigs of length N50 or longer), NG50 or U50 (which corrects misrepresented N50 values in high background noise sequences). This evaluation can be carried out via algorithms QUAST [256], Assemblathon [257], GAGE [258] and BUSCO [259]. 

#### 3.6.3. Analysis of the Results

It is necessary to verify the viral origin of the contigs and/or scaffold produced in the previous step, especially in mixed samples, in which other organisms may be present in addition to the virus of interest. This can be done by comparing sequences with reference sequence databases (such as GenBank non-redundant (NR), nucleotide (NT), Refseq viral, Uniprot viral) or custom viral databases generated in-house and using BLASTx (for comparison with protein databases), blastn (for comparison with nucleotide databases) and DIAMOND [260] (translated protein search mode). Alternatively the HMMER [261] (http://hmmer.org/, accessed on 28 June 2022) algorithm can be used to detect true homologs rather than traditional BLAST-based approaches, based on the fact that certain positions in a sequence alignment are likely to differ in their probability of containing an insertion or a deletion [262]. Finally, through the use of the Sequence Demarcation Tool program, it is possible to verify the result of the previous analysis using a graphic approach that can make it easier to identify the sequences where the similarity is stronger. It is also possible to use the nucleotide or reconstructed amino acid sequence of the virus for phylogenetic analysis. This procedure is carried out by aligning the viral genome sequence with other reference sequences (ideally of similar length), and the result provides information about homology with viruses of different species. This aspect can be very important in the field of public health, such as in the discovery of viruses responsible for new outbreaks of infection, as a potential pathogenic virus can be recognized and investigated by analyzing the epidemiological link between genetic sequences of other pathogens. The most used algorithms to perform this analysis are CLUSTAL, MUSCLE, MAFFT, T-Coffee [263], (available from: https://www.ebi.ac.uk/Tools/msa/, accessed on 28 June 2022) and Megan [264], which, in addition to taxonomic analysis, also allows functional analysis, generation of graphs, clustering and networks analysis. 

Having obtained the complete (or almost complete) genomic sequence, it is possible to proceed to the presumed identification of the open reading frames (ORF), which is performed by prediction. The main algorithms that are used for this process are NCBI ORF FINDER, Glimmer (Gene Locator and Interpolated Markov ModelER) and Geneious. Estimation of the relationships between the identified sequence of the virus and its common ancestors [265] or between sequences that supposedly contain genes to assume their function [266] is carried out through the creation of phylogenetic trees, estimated through different methods (Neighbor Joining, UPGMA Maximum Parsiony, Bayesian Inference and Maximum Likelihood [ML]) [267] and algorithms (MEGA, PhyML and IQ-Tree).

## 4. Discussion

mNGS is one of the most rapidly evolving fields of biology, allowing broadening of our understanding of diversity, ecology and the evolution of microbial communities from different habitats. Its application to the identification of new pathogens or for monitoring known agents in clinical and environmental samples makes it an instrument of choice in the One Health prevention approach. This strategy is based on the awareness that human health is closely linked to that of animals and the environment, an awareness that also aims at reducing the risk of potential epidemics [268,269,270,271,272]. This review highlighted that the application of NGS technologies is currently feasible also in middle and low-income countries, mainly thanks to international collaborations [45,49,104,151,273,274,275]. In these regions, costs associated with infrastructure, equipment, reagents and expertise could pose serious challenges to the use of NGS for pathogen identification [276]; one possible proposal to overcome this limitation may be the establishment of omics international networks.

mNGS allows primer-independent, unbiased detection of the viroma and the reconstruction of full-length viral genomes, even in the case of unknown or poorly characterized viruses [17,277,278], comprising bacteriophages, providing an unprecedented opportunity for the discovery of novel viruses [180]. Since viruses do not share conserved sequences, the definition of viroma is obtainable exclusively through shotgun metagenomic sequencing of the entire microbial community.

This review summarizes previous studies related to the use of mNGS for the identification of new viruses from different types of samples (animal, plant and environmental), through a systematic review of the literature [279,280]. The different processes involved in the studies include processing of different sample types for nucleic acid purification, sequencing and bioinformatics data analysis. For each of these aspects, the literature analysis has highlighted heterogeneous approaches that make it impossible to compare the results but allow the identification of new viruses from different matrices using a plethora of different strategies, responding flexibly to different research questions.

As regards the purification of nucleic acids, the methods used are based on commercial kits producing similar samples in terms of purified microbial communities [281,282]. The different kits used are indicated for the purification of DNA, RNA, or both; however, it has been shown that identification of RNA virus (*Norovirus*) by mNGS in biological samples such as feces is also possible from nucleic acid obtained by a DNA purification kit, after a retro-transcription step before library preparation [178]. For environmental samples, different sample enrichment strategies before purification have been identified, based on tangential flow filtration, pore filtration, PEG precipitation and FeCl3 precipitation, ultracentrifugation, fluidic circuit, chemical flocculation and syringe filtration, and it has been previously shown that viral richness, viral specificity, viral pathogen detection and viral community composition for metagenomic analyses are influenced by concentration protocols [283]. 

It has been shown that the use of pre-extraction enrichment methods can introduce bias in the identification of microbial species present in the sample [283]. For example, filtration can reduce the abundance of bacterial [284] or viral species [285,286], depending on pore size dimension. Similarly, enrichment based on low-force centrifugation can induce depletion of large viruses [285]. The use of PEG for the identification of viral species from wastewater induced a better recovery of *Adenovirus* but a lower recovery efficiency of Human Rotavirus A, compared to methods based on charged membrane or glass wool [287], while enrichment methods based on microfluidics devices have proven to be effective in the characterization of airway microbiomes [288].

To improve shotgun metagenomics results some post-extraction enrichment technologies have also been developed: they aim to reduce host nucleic acid sequences, enriching samples for microbial genomes. Thanks to these methods, an increased number of microbial reads could be obtained from sequencing, improving the number of species and taxa detected and the coverage of each sequence and allowing detection of less-abundant species. Further, they could reduce costs associated with mNGS, since more samples can be analyzed in the same sequencing run. Some enrichment technologies used to remove human DNA from samples have been described [289,290]. In addition, some commercial kits are available, and their efficiency in human DNA depletion has been tested and compared [289,291,292,293]. However, bias in the phylogenetic composition of samples could be introduced by using these post-extraction enrichment methods because of the unspecific removal of some bacterial DNA [289,290,291,293].

With regard to library preparation methods and sequencing platforms, a decrease in heterogeneity has been observed over the years, thanks to the gradual increase in the use of Illumina, which is currently the most widespread among the NGS methods available. 

Millions of sequence reads are generated from a single run and must be analyzed through dedicated software and bioinformatics pipelines, to produce meaningful results [19,243,294,295]. The various published articles show different algorithms for metagenomic analysis or different reference databases, but present common steps in the bioinformatics analysis process, which has been described in detail and allows the identification of known and new viruses in the samples.

However, it is possible that erroneous chimeric sequences could be generated from multiple related viruses, if present in the same sample, or even between viral and non-viral sequences. It has been suggested that the use of bioinformatic pipelines that include a chimera checking step could improve the quality of sequencing data [296]. Another possible approach to improve metagenome assemblies from community microbial samples could be the use of sequencing platforms that generate long-range reads [297,298], such as Oxford Nanopore Technologies (ONT, Oxford, UK) [299], Pacific Biosciences (PacBio) [300], 10X Genomics (Pleasanton, CA, USA) [301] and Hi-C [298]. Moreover, mixed approaches, which associate the use of long-range and short nucleotide technologies, could improve the quality of the data [302].

Although there are not many published reports in which the sensitivity of mNGS in identifying viral species is evaluated, it is known that this may vary depending on the sequencing platform used, the depth of sequencing, and the type of virus present in the sample. Frey et al. [303] found that the sensitivity of both Illumina MiSeq and Ion Torrent PGM in identifying Influenza A virus in blood samples stood at 10^4^ genome copies/mL, using an Ion 314 chip for Ion Torrent PGM with an output of 30–50 million single-end reads, and a 300 cycle kit for Illumina MiSeq with an output of 24–30 million paired-end reads. Be et al. [304] found that the sensitivity of Illumina GA IIx to identify B. antracis in Aerosol DNA extract and Soil DNA extract is 10 GEs and 10^2^ GEs, respectively, with a depth of 37.5 million single-end reads/sample (1 sample per lane). Bukowska-Osko et al. [305] detected the presence of HIV in CSF HIV RNA-positive samples containing at least 10^2^ viral copies/mL; they also detected the presence of HSV-1 DNA in CSF samples containing at least 10^3^ viral copies/µL, using an Illumina Hi-Seq 1500 and with a depth of about 33 million reads/sample.

In conclusion, despite the lack of standard protocols from the sampling phase to the production of the interpreted data, the different methods currently existing at each stage of the process offer effective tools for the exploration of the earth viroma (animal, plant, environment). The lack of standardization, moreover, seems to be better suited to the exploration of the sequences that are generated by sequencing but that are not attributable to any known organism/sequence. In fact, the continuous development of new analysis tools also allows the study of previously generated High Throughput Sequencing datasets, which allow the detection of new viral sequences, even pathogenic ones, not previously recognized in the samples analyzed. Though the lack of standardized approaches is currently a constraint on the use of this technology in the regulatory area, for example, for the monitoring of zoonoses or water quality by official control bodies, which require standardized processes [33], mNGS technologies have proven to be suitable and crucial for tracking novel SARS-CoV-2 hosts, evolution, and spread patterns [17,24]. mNGS is characterized by a constant and ultra-rapid development of new analytical methods, both biological and bioinformatics, that seem better suited to follow the dynamic of pandemic events and in general viral outbreaks. This suggests the opportunity to implement these technologies to establish early warning systems [74] and to design effective disease control and prevention strategies. 

## Figures and Tables

**Table 1:** Examples of novel viruses found by mNGS studies.

Sample Type	Novel Viruses Found in mNGS Studies	References
**Animal**	*Flavivirus*	[45,67,85,147,212,213]
	*Coronanvirus*	[25,214,215]
	*Circovirus*	[63,120,159,216,217]
	*Bocaparvovirus*	[121,124,200]
	*Siphoviridae*, *Myoviridae*, *Podoviridae*, *crAss-like viruses*	[195]
	*Sapovirus*	[44,95,218].
**Plant**	*Prunus Geminivirus*	[207]
	*Mastrevirus*	[219,220,221]
	*Begomovirus*	[132,209]
	*Genomovirus*	[222]
	*Narnavirus*	[223]
	*Tepovirus*	[224]
**Environment**	*Phycodnavirus*	[210,225]
	*Picornavirus*	[50,226]
	*PA-SR01*	[163]
	*Picobirnaviridae*	[40]
	*Epatitis E virus*	[171]
	*Methanosarcina virus MV* (*MetMV*)	[227]
	*Halovirus*	[42,228]
	*SAR11 phage*	[185]

**Table 2:** Most commonly used programs/algorithms for QC protocol.

Software	Reference	Available at
FastQC	[230]	https://www.bioinformatics.babraham.ac.uk/projects/fastqc/ (accessed on 28 June 2022)
Trimmomatic	[231]	
Trim Galore	[232]	https://www.bioinformatics.babraham.ac.uk/projects/trim_galore/ (accessed on 28 June 2022)
Cutadapt	[233]	https://journal.embnet.org/index.php/embnetjournal/article/view/200/479 (accessed on 28 June 2022)
CLC Genomics Workbench (Qiagen)	[234]	https://digitalinsights.qiagen.com/products-overview/discovery-insights-portfolio/qiagen-clc-genomics/ (accessed on 28 June 2022)
BBDuk (part of BBTools/BBMap package)	[235]	https://jgi.doe.gov/data-and-tools/software-tools/bbtools/ (accessed on 28 June 2022)

**Table 3:** Most-used tools for metagenomic assembly.

Software	Reference
SPAdes	[31]
MetaSPAdes	[244]
Megahit	[245]
Velvet	[246]
Trinity	[247]
SOAPdenovo2	[248]
Ensemble Assembler	[249]
MIRA	[250]
PRICE	[251]
Codon Code Aligner	[252]; https://www.codoncode.com/aligner/ (accessed on 28 June 2022)
ABySS	[253]
Ray Meta	[254]
CAP3	[255]
CLC Genomics Workbench (Qiagen)	[234]

## Data Availability

All data generated or analyzed during this study are included in this published article and its supplementary information files. The dataset supporting the conclusion of this article is included within the article and its additional files.

## Supplementary Materials

The following supporting information can be downloaded at: https://www.mdpi.com/article/10.3390/life12122048/s1, Figure S1: Use of the “de novo Assembly”, Table S1: List of commercial kits employed, Table S2: Characteristic table and list of the included studies.
