# Supplementary material for: Novel Virus Identification through Metagenomics: A Systematic Review
Source: Life (Basel). 2022 Dec 7;12(12):2048. doi: 10.3390/life12122048 (PMC9784588; doi:10.3390/life12122048)
Supplement: Supplementary file 1 [file life-12-02048-s001.zip › Bassi_Table S1.pdf]

Table S1: list of commercial kits employed

Extraction kit	Manufacturer	Genome	Extraction method	Specimen	n	References
Agencourt Formapure Kit	Beckman Coulter	DNA/RNA	Magnetic beads-based	brain tissue	1	[169]
Ampliprep DNA extraction machine	Roche	DNA	Magnetic beads-based	stool samples	1	[147]
AMPure beads	PacBio	DNA	Magnetic beads-based	faecal samples	1	[298]
beads, thermo fisher	ThermoFisher	RNA	Magnetic beads-based	potato leaf	1	[36]
Cador Qiagen Pathogen minikit	Qiagen	DNA/RNA	Column-based	mucus samples	1	[92]
ChargeSwitch gDNA Mini Bacteria Kit	ThermoFisher	DNA	Magnetic beads-based	water	1	[31]
Chemagic MSM I	Perkin Elmer	DNA/RNA	Magnetic beads-based	fecal samples	1	[377]
Direct-zol RNA Miniprep kit	Zymo Research	RNA	Column-based	fecal sample	1	[104]
DNA/RNA Viral Extraction Kit	Invitrogen	DNA/RNA	Silica membrane-based	hot spring samples	1	[40]
DNeasy Blood & Tissue Kit	Qiagen	DNA	Column-based	fish, tumor tissue, venous blood, spleen, thymus, and lung, wastewater, Hepatopancreas tissue, water	6	[19,51,84,133,305,348]
DNeasy Plant DNA extraction kit	Qiagen	DNA	Silica membrane-based	Leaf	1	[65]
DNeasy Plant Mini Kit	Qiagen	DNA	Column-based	plants, mummified peach fruits	2	[121,155]

E.Z.N.A. Plant Kit	Omega Biotek	DNA	Magnetic beads-based	leaf	1	[297]
Extract-N-Amp	Sigma–Aldrich	DNA	Solvent-based	larvae	1	[79]
EZ1 Advanced XL device	Qiagen	DNA/RNA	Magnetic beads-based	sewage sample	1	[15]
FastDNA Kit	MP Biomedicals	DNA	Silica membrane-based	freshwater	1	[262]
GeneJet Viral DNA and RNA Purification Kit	ThermoFisher	DNA/RNA	Silica membrane-based	bat guano, fecal sample	2	[25,160]
Genjet RNA extraction kit	ThermoFisher	RNA	Column-based	ticks homogenization	1	[35]
Genomic-tip 20/G purification kit	Qiagen	DNA	Column-based	seawater	1	[27]
High Pure template preparation kit	Roche	DNA	Filter tube	intestinal contents	1	[91]
High Pure Viral Nucleic Acid kit	Roche	DNA/RNA	Filter tube	swab samples, sewage and reclaimed water samples, buccal swab, cassava leaf, brains and hemolymph, blackflies homogenization, faeces, mosquito homogenization, Fecal Samples, blood, lung, liver, spleen, kidney and intestinal contents	11	[37,56,70,78,165,166,183,213,214,232,276]
High pure viral nucleic acid large volume kit	Roche	RNA	Column-based	insect	1	[205]
High Pure Viral RNA Kit	Roche	RNA	Filter tube	Salivary gland, raw sewage	3	[73,127,188]
iPrep PureLink Virus Kit	Life Technologies	DNA/RNA	Other/multiple type	bat guano	1	[135]

iPrep virus kit	Invitrogen	DNA/RNA	Other/multiple type	guano, feces and saliva	2	[286,287]
ISGEN LS	NipponGene	RNA	Solvent-based	fecal samples	1	[189]
Isogen II	NipponGene	RNA	Solvent-based	mosquitoes	1	[102]
Mag-Bind Viral DNA/RNA kit	Omega Biotek	DNA/RNA	Magnetic beads-based	mosquitoes	1	[103]
MagMAX Express-96 Deep Well Magnetic Particle Processor	ThermoFisher	DNA/RNA	Magnetic beads-based	mosquitoes homogenization	2	[270,347]
MagMAX Viral Isolation Kit	ThermoFisher	DNA/RNA	Magnetic beads-based	bee homogenization	1	[120]
MagMAX Viral RNA Isolation kit	ThermoFisher	RNA	Magnetic beads-based	Spleen, liver, lung, kidney, and small intestine, tissue pools, mesenteric lymph node, lung swab, Gut tissue, nasal swabs, cerebrospinal fluid, Feces, plasma, cloacal swabs, fecal sample, nasopharyngeal aspirates, serum pools, mosquito pool, oral sample, neat piece of meats	23	[7-13,97,100,101,107,140,229,247,271,272,282,319,320,342,343,358,219]
MagMAX-96 Viral RNA Isolation Kit	Ambion	RNA	Magnetic beads-based	heart and lung tissues homogenate	1	[228]
MagNA Pure 96 system	Roche	DNA/RNA	Magnetic glass particles-based	fecal sample	1	[176]
MagNA Pure Large Volume kit	Roche	DNA/RNA	Magnetic glass particles-based	Ticks homogenization	1	[222]
MagNA Pure LC - DNA Kit	Roche	DNA	Magnetic glass particles-based	maize samples	1	[344]
MagNA Pure LC - Total Nucleic Acid Kit	Roche	DNA/RNA	Magnetic glass particles-based	swab samples, Serum and CSF	2	[17,292]

MasterPure Complete DNA and RNA Purification kit	Biosearch Technologies	DNA/RNA	Solvent-based	mosquitos, seawater	2	[58,193]
Maxwell 16 automated extractor	Promega	DNA/RNA	Magnetic beads-based	serum, diarrhea samples, fecal samples	3	[225-227]
Maxwell 16 viral total nucleic acid purification kit	Promega	DNA/RNA	Magnetic beads-based	blood	1	[157]
MiniBEST Universal Genomic DNA Extraction Kit	TaKaRa	DNA	Column-based	fecal samples, intestinal content samples	3	[266-268]
MiniBEST Viral RNA/DNA Extraction Kit	TaKaRa	DNA/RNA	Column-based	fecal samples	1	[354]
mirVana miRNA isolation kit	ThermoFisher	RNA	Column-based	hot spring	1	[39]
NEBNext Ultra RNA Library Prep Kit for Illumina	NEB	RNA	Magnetic beads-based	fecal samples	1	[210]
Nucleon Bacc2 kit	GE Healthcare	DNA	Solvent-based	brain tissue	1	[44]
NucleoSpin 96 Virus Core Kit	Macherey-Nagel	DNA/RNA	Silica membrane-based	leaf samples, mites homogenization	3	[110,115,216]
Nucleospin Extract II kit	Macherey–Nagel	DNA	Silica membrane-based	peatsoilandwater	1	[238]
NucleoSpin kit	Macherey–Nagel	DNA/RNA	Silica membrane-based	Wild Poaceae	1	[77]
Nucleospin RNA II kit	Macherey–Nagel	RNA	Silica membrane-based	ticks	1	[199]

NucleoSpin RNA Stool kit	Macherey–Nagel	RNA	Silica membrane-based	fecal sample	1	[1]
Nucleospin RNA XS Kit	Macherey–Nagel	RNA	Silica membrane-based	Intestinal contents	1	[330]
NucleoSpin RNA Midi kits	Macherey–Nagel	RNA	Silica membrane-based	water	1	[50]
NucliSens easyMAG system	bioMérieux	DNA/RNA	Other/multiple type	nasopharyngeal aspirate samples	1	[173]
NZYol	NZYTech	RNA	Solvent-based	liver, skin, gills	1	[108]
Phage DNA isolation kit	Norgen Biotek	DNA	Column-based	Intesti bacteriophage	1	[379]
PowerSoil DNA Isolation kit	Mo Bio	DNA	Silica membrane-based	Intesti bacteriophage, wastewater, seaflor, air, soil	4	[128,215,242,280]
PowerSoil Max kit	MO BIO	DNA	Silica membrane-based	compost sample, soil and water	2	[14,198]
PowerSoil RNA Isolation kit	MO BIO	RNA	Column-based	water	1	[348]
PureLink Microbiome DNA purification kit	Invitrogen	DNA	Column-based	Plant Metagenome	1	[105]
PureLink Viral DNA and RNA extraction kit	Invitrogen	DNA/RNA	Column-based	hot spring water sample	1	[146]
QIAamp 96 Virus QIAcube HT kit	Qiagen	DNA/RNA	Silica membrane-based	Mosquito homogenization, nasal-throat swabs	2	[273,317]
QIAamp Circulating Nucleic Acid Kit	Qiagen	DNA/RNA	Silica membrane-based	blood samples	1	[164]
QIAamp DNA blood mini kit	Qiagen	DNA	Silica membrane-based	plasma	1	[265]

QIAamp DNA FFPE Tissue Kit	Qiagen	DNA	Column-based	Plant Metagenome	1	[105]
QIAamp DNA Mini and Blood kit	Qiagen	DNA	Silica membrane-based	nasopharyngeal aspirate samples	1	[173]
QIAamp DNA Mini Kit	Qiagen	DNA	Silica membrane-based	brain tissue, serum, lymph nodes, nasopharyngeal swabs, plasma, water	7	[32-34,53,57,72,260]
QIAamp DNA Stool kit	Qiagen	DNA	Silica membrane-based	fecal sample, feces	3	[137,192,201]
QIAamp Fast DNA Stool Mini Kit	Qiagen	DNA	Silica membrane-based	surface water	1	[124]
QIAamp MinElute Virus Spin Kit	Qiagen	DNA/RNA	Column-based	wastewater, feces, blood, fecal samples, copepods, oral secretion, nasal secretion, genital secretion, skin swab, water, heart, liver, spleen, lung, and kidney, insects homogenization, mussels, dragonfly abdomens, dragonflies, variety of marine invertebrate species,adult whiteflies, Conjunctival swabs, oral swabs, fibropapilloma swabs, bone marrow, ballast water	22	[20,55,75,76,93,132,150,177,181,250, 256-259,299,301,307,308,350,356,359,376]
QIAamp MinEluteVirus Vacuum Kit	Qiagen	DNA/RNA	Other/multiple type	feces	1	[60]
QIAamp spin-column	Qiagen	DNA/RNA	Column-based	fecal samples, cloacal and tissue samples, sewage waters	4	[138,219,246,302]
QIAamp UCP Micro Kit	Qiagen	DNA/RNA	Column-based	insect	1	[315]
QIAamp viral DNA kit	Qiagen	DNA	Column-based	samples of shrimp	1	[237]

QIAamp viral RNA kit	Qiagen	RNA	Silica membrane-based	serum, nasal and throat swabs, stools, cerebrospinal fluid, water, faecal sample, several tissues, feces, whole-blood, mosquitoes homogenization, infected gingival epithelium samples, periodontal pockets, gingival epithelium, and connective tissue facing the sulcus	9	[16,122,217,231,283,309,355,364,366]
QIAamp Viral RNA Mini Elute Kit	Qiagen	RNA	Column-based	liver, heart, intestine and stomach	1	[352]
QIAamp viral RNA mini kit	Qiagen	RNA	Other/multiple type	faeces, mosquitoes homogenization, mosquitoes and midges, diarrheic stools, swab and cloacal samples, stool samples, parsley leaves, mosquitoes, oral, throat and anal swab samples, serum samples, fecal samples, blood, bat guano, plasma, nasal swab, mixed biological samples, plasma, vaginal swab, insect, C6/36 cells (Aedes albopictus), cell culture, Mosquito homogenization, tissue pools from liver and lymph nodes, Tick homogenization, bovine serum, brain tumor samples, feline feces, Rectal swabs, nasopharyngeal secretion, heart, liver, spleen, lung, and kidney tissues, gut	53	[21,24,66,67,81,94,106,117,118,125,129,136,139,151,159,168,170,171,174,175,182,187,221,234-236,240,241,245,264,275,285,288,289,294,300,303,310,316,321,324,331-334,340,351,353,362,363,367,369,372]
QIAamp virus minikit	Qiagen	DNA/RNA	Column-based	fecal sample	1	[357]
Qiamp Viral RNA isolation kit	Qiagen	RNA	Column-based	insect pools, skunk brain, human feces and sewer effluent	1	[335]

QIAprep Spin Miniprep kit	Qiagen	DNA	Column-based	deep Mediterranean Sea water	1	[196]
QIAquick viral RNA column purification system	Qiagen	RNA	Column-based	skin	1	[89]
QIAseq FX DNA Library Kit	Qiagen	DNA	Magnetic beads-based	serum	1	[98]
QIAsymphony	Qiagen	DNA/RNA	Other/multiple type	cerebral biopsy	1	[252]
Ribopure RNA extraction kit	Life Technologies	RNA	Other/multiple type	gut content	1	[87]
RNA viral extraction kit	Qiagen	RNA	Other/multiple type	honey bees homogenization	1	[83]
RNeasy kit	Qiagen	RNA	Column-based	lungs, liver and brain, spinal cord and spleen material	2	[74,223]
RNeasy Lipid Tissue Mini kit	Qiagen	RNA	Column-based	bee thorax, swabs and tissue	2	[279,318]
RNeasy Mini Kit	Qiagen	RNA	Silica membrane-based	mosquitos, guts, lungs, kidney, hot spring samples, brain tissue, faecal samples	7	[30,40,42,44,144,253,278]
RNeasy Plant Mini Kit	Qiagen	RNA	Column-based	leaves, plant	5	[52,109,145,161,199]
RNeasy Plus Mini Kit	Qiagen	RNA	Column-based	faecal, liver, and ectoparasite samples, Dead fish, Ticks homogenization, Brain, heart, seawater	6	[27,46,62,123,141,233]
Spectrum Plant Total RNA kit instructions	Sigma-Aldrich	RNA	Column-based	fungus	1	[263]
SV Total Viral RNA mini kit	Qiagen	RNA	Column-based	neonatal pig diarrhea	1	[284]

TRI reagent	Sigma-Aldrich	DNA/RNA	Solvent-based	faecal sample, spleen and lung, A. mellifera and mite samples	4	[42,48,142,178]
Viral DNA kit	Zymo Research	DNA	Column-based	pyloric caeca and body wall tissue	1	[153]
Viral Gene-spin Viral DNA/RNA Extraction Kit	iNtRON Biotechnolog y	DNA/RNA	Column-based	proventriculus samples	1	[131]
Wizard DNA purification kit	Promega	DNA	Solvent-based	insect	1	[116]
Wizard Prep kit	Promega	DNA	Column-based	water	1	[261]
ZR Viral DNA/RNA Kit	Zymo Research	DNA/RNA	Other/multiple type	leafhopper homogenization, faeces, fecal samples	3	[112,113,311]
ZymoBIOMICS DNA kits	MoBio	DNA	Other/multiple type	water	1	[54]
