# Supplementary material for: Novel Virus Identification through Metagenomics: A Systematic Review
Source: Life (Basel). 2022 Dec 7;12(12):2048. doi: 10.3390/life12122048 (PMC9784588; doi:10.3390/life12122048)
Supplement: Supplementary file 1 [file life-12-02048-s001.zip › Bassi_Table S2.pdf]

Table S2: Characteristic Table and list of the included studies

n.Ref	Year	Sample size	Host	Specimen	Genome	Sequencing	Assembly	Virus	Viral Families	Provenance of the sample	WHO Regions	Extraction kits	Extraction methods	Enrichment methods
[1]	2021	195	animal	fecal samples	RNA	illumina	De novo	Aichivirus D	Picornaviridae,	China	South-East Asia region	NucleoSpin RNA Stool kit (Macherey-Nagel)	Silica membrane-based	
[2]	2015	6	environment	water	DNA	illumina	/	Several	Circoviridae, Unclassified	Arctic		Phenol-chloroform extraction method	Solvent-based	tangential flow filtration
[3]	2018	1	plant	plants	RNA	illumina	/	prunus geminivirus	Geminiviridae,	California	America region	/	ND	
[4]	2019	2	environment	water	DNA	illumina	De novo	Phycodnavirus	Phycodnaviridae,	Chile	America region	/	ND	pore filtration
[5]	2021	5	environment	water	DNA	illumina	/	Pp_CBA virus, PaV virus	Myoviridae, Phycodnaviridae	Southern Ocean		Formamide Procedure	Solvent-based	tangential flow filtration
[6]	2014	1	animal	blood	RNA	Roche_454	/	paramyxovirus	Paramyxoviridae,	Uganda	Africa region	/	ND	
[7]	2018	6	animal	tissue pools	DNA	illumina	De novo	amdoparvovirus	Parvoviridae,	Sacramento	America region	MagMAX Viral RNA Isolation kit	Magnetic beads-based	
[8]	2020	17	animal	tissue pools	DNA	illumina	De novo	Circoviridae, Parvoviridae, Anelloviridae, Polyomaviridae, and Papillomaviridae	Parvoviridae, Anelloviridae, Circoviridae, Papillomaviridae, Polyomaviridae,	California	America region	MagMAX Viral RNA Isolation kit	Magnetic beads-based	
[9]	2020	1	animal	mesenteric lymph node	DNA	illumina	De novo	parvovirus, polyomavirus, rotavirus, anellovirus	Parvoviridae, Anelloviridae, Reoviridae, Polyomaviridae	California	America region	MagMAX Viral RNA Isolation kit	Magnetic beads-based	
[10]	2021	6	animal	lung swab	DNA and RNA	illumina	De novo	Parvoviruses and Picornavirus	Picornaviridae, Parvoviridae	California	America region	MagMAX Viral RNA Isolation kit	Magnetic beads-based	
[11]	2019	ND	animal	gut	RNA	illumina	De novo	Cyprivirus	Picornaviridae,	North America, Europe, and Asia	America region, European region and	MagMAX Viral RNA Isolation kit	Magnetic beads-based	

											South-East Asia region			
[12]	2019	68	animal	plasma and cerobrospinal fluid	DNA	Roche_454	De novo	parvovirus	Parvoviridae,	/		MagMAX Viral RNA Isolation kit	Magnetic beads-based	
[13]	2019	57	animal	nasal swabs	DNA	illumina	De novo	taupapillomaviruses	Papillomaviridae,	/		MagMAX Viral RNA Isolation kit	Magnetic beads-based	
[14]	2017	ND	environment	compost sample	DNA	illumina	/	Pseudomonas phages	Siphoviridae,	Brazil	America region	PowerSoil Max kit (MO BIO)	Silica membrane-based	pore filtration
[15]	2021	1	environment	sewage waters	DNA	illumina	De novo	Fadolivirus	Mimiviridae,	Algeria	Africa region	EZ1 Advanced XL device (Qiagen)	Magnetic beads-based	
[16]	2019	492	animal	serum, nasal and throat swabs, stools, cerebrospinal fluid	DNA and RNA	illumina	De novo	several	Adenoviridae, Coronaviridae, Nodaviridae, Picornaviridae, Picobirnaviridae, Parvoviridae, Papillomaviridae, Partitiviridae, Reoviridae, Rhabdoviridae, Totiviridae, Unclassified	Vietnam	South-East Asia region	QIAamp viral RNA kit (Qiagen)	Silica membrane-based	
[17]	2015	40	animal	swab samples	DNA	illumina	/	hpv	Papillomaviridae,	Sweden	European region	MagNA Pure LC - Total Nucleic Acid Kit (Roche)	Magnetic glass particles-based	
[18]	2014	Database	animal	muscle tissue	DNA	illumina and sanger	/	Herpesvirus	Herpesviridae,	Germany	European region	/	ND	
[19]	2017	Database	animal	fish	DNA	sanger	/	Herpesvirus	Herpesviridae,	Oxford	European region	DNeasy Blood & Tissue Kit (Qiagen)	Column-based	
[20]	2014	2	environment	wastewater	DNA	illumina	/	Cyclovirus	Cycloviridae,	Philippine	South-East Asia region	QIAamp MinElute Virus Spin Kit (Qiagen), RNeasy Plus Mini Kit (Qiagen)	Column-based, Column-based	PEG precipitation
[21]	2014	27	animal	fecal samples	DNA and RNA	illumina	De novo	sapovirus, kobuvirus, PBV, PBoV-2 and PBoV-4	Caliciviridae, Picornaviridae	China	South-East Asia region	QIAamp viral RNA mini kit (Qiagen)	Other/multiple type	

[22]	2019	3	environment	sediments	DNA	illumina	De novo	pythoviruses, marseilleviruses, iridoviruses, klosneuviruses	Pithoviridae, Marseilleviridae, Iridoviridae, Mimiviridae	Atlantic Ocean		/	ND	ultracentrifu gation
[23]	2015	2	plant	wood tissue	RNA	illumina	De novo	Luteovirus	Luteoviridae,	California	America region	/	ND	
[24]	2020	ND	animal	insects	RNA	illumina	/	flaviviruses	Flaviviridae,	Cambodia and Thailand	Western Pacific region	QIAamp viral RNA mini kit (Qiagen)	Other/multip le type	
[25]	2017	10	animal	fecal samples	RNA	Ion Torrent	De novo	rotaviruses	Reoviridae,	Serbia	European region	GeneJet Viral DNA and RNA Purification Kit (Thermo Fisher)	Column-based	
[26]	2018	Database	animal	nasopharynge al aspirates, serum, feces, and cerebrospinal fluid	DNA	illumina	/	Torque Teno virus (TTV)	Anelloviridae,	Sweden	European region	/	ND	
[27]	2020	3	environment	seawater	DNA	illumina	/	Pelagiphages	Podoviridae,	Hawaii	America region	Genomic-tip 20/G purification kit (Qiagen)	Column-based	tangential flow filtration
[28]	2019	12	environment	water	DNA	illumina	De novo	Mamiellales SAG-associated circular virus' (MACV)	Unclassified,	Indian Ocean		multiple displacement amplification (MDA)	Other/multip le type	tangential flow filtration
[29]	2020	91	plant	wood tissue	RNA	illumina	De novo	Potyviridae and Bunyavirales order	Peribunyaviridae, Potyviridae	Several eupean country	European region	Phenol-chloroform extraction method	Solvent-based	
[30]	2020	30	animal	insects	RNA	illumina	De novo	Bunyavirales	Peribunyaviridae,	Spain	European region	RNeasy Mini Kit (Qiagen)	Silica membrane-based	
[31]	2019	ND	environment	water	DNA	illumina	/	cobaviruses phage	Podoviridae,	North Sea	America region	ChargeSwitc h gDNA Mini Bacteria Kit (ThermoFish er Scientific)	Magnetic beads-based	pore filtration
[32]	2010	3	animal	brain sample	RNA	Roche_454	/	astrovirus	Astroviridae,	Denmark	European region	QIAamp DNA Mini Kit (Qiagen) , RNeasy mini kit (Qiagen)	Silica membrane-based, Silica membrane-based	
[33]	2012	3	animal	serum	DNA	Roche_454	/	TTSuV1, TTSuV2, Porcine parvovirus 4	Anelloviridae, Anelloviridae, Parvoviridae,	Uganda	Africa region	QIAamp DNA Mini Kit (Qiagen) ,	Silica membrane-based, Silica	

												RNeasy mini kit (Qiagen)	membrane-based	
[34]	2016	8	animal	lymph nodes	RNA	Roche_454	De novo	circoviruses	Circoviridae,	Sweden	European region	QIAamp DNA Mini Kit (Qiagen) , RNeasy mini kit (Qiagen)	Silica membrane-based, Silica membrane-based	
[35]	2019	180	animal	insects	RNA	illumina	De novo	Nairoviridae family	Bunyaviridae,	Brazil	America region	Genjet RNA extraction kit (Thermo Fisher)	Column-based	
[36]	2016	ND	plant	leaves	RNA	illumina	De novo	Macluravirus	Potyviridae,	New Zealand	South-East Asia region	beads, termo fisher	Magnetic beads-based	
[37]	2015	ND	animal	swab samples	DNA	Roche_454	/	Gammaherpesvirus	Herpesviridae,	Netherlands	European region	High Pure Viral Nucleic Acid kit (Roche)	Filter tube	
[38]	2020	98	animal	oral/anal swab	DNA	illumina	De novo	Gemykibivirus, Papillomavirus	Genomoviridae, Papillomaviridae	Argentina	America region	/	ND	
[39]	2012	7	environment	hot spring samples	RNA	Roche_454	/	Nodaviruses, Tetraviruses, Birnaviruses	Nodaviridae, Tetraviridae, Birnaviridae,	United States	America region	mirVana miRNA isolation kit	Column-based	pore filtration
[40]	2015	10	environment	hot spring samples	DNA	illumina	/	several	/,	United States	America region	DNA/RNA Viral Extraction Kit (Invitrogen)	Silica membrane-based	ultracentrifu gation
[41]	2020	ND	environment	water	DNA	illumina	De novo	Yaravirus	Unclassified,	Brazil	America region	/	ND	ultracentrifu gation
[42]	2020	111	animal	fecal samples	RNA	illumina	/	Sapelovirus (family Picornaviridae)	Picornaviridae,	Hungary	European region	TRI reagent (Sigma-Aldrich)	Solvent-based	
[43]	2017	717	plant	leaves	DNA	Roche_454	De novo	Mastrevirus	Geminiviridae,	Florida and Guadeloupe	America region	/	ND	
[44]	2014	25	animal	brain sample	RNA	illumina	De novo	Astrovirus	Astroviridae,	Switzerland	European region	Nucleon Bacc2 kit (GE Healthcare), TRIzol reagents	Solvent-based, Solvent-based	

[45]	2016	7	animal	Trematomus pennellii	DNA	illumina	De novo	polyomavirus	Polyomaviridae,	Ross Sea		/	ND	
[46]	2020	27	animal	fecal samples, liver, and ectoparasite samples	RNA	illumina	De novo	picornavirus	Picornaviridae,	Australia	South-East Asia region	RNeasy Plus Mini Kit (Qiagen)	Column-based	
[47]	2013	ND	plant	tomato	RNA	Roche_454	/	Totiviridae and Partitiviridae	Partiviridae,	France	European region	/	ND	
[48]	2021	29	animal	spleen and lung	DNA	illumina	De novo	Anellovirus, Circovirus	Anelloviridae, Circoviridae	South American	America region	TRI reagent (Sigma-Aldrich)	Solvent-based	
[49]	2017	54	animal	organs and skin lesions	DNA	Ion Torrent	De novo	Orthopoxvirus	Poxviridae,	Italy	European region	/	ND	
[50]	2018	68	environment	water	DNA and RNA	illumina	/	Chuviridae, Yanvirus, Weivirus, Zhaovirus, Qinvirus, Yuevirus	Chuviridae, Unclassified, Unclassified, Unclassified, Qinviridae, Yueviridae	/		NucleoSpin RNA Midi kits (Macherey-Nagel)	Silica membrane-based	
[51]	2020	1	animal	tumor tissue	DNA	illumina	De novo	papillomavirus	Papillomaviridae,	Australia	South-East Asia region	DNeasy Blood & Tissue Kit (Qiagen)	Column-based	
[52]	2021	10	plant	leaves	RNA	illumina	De novo	Iflavirus	Iflaviridae,	United States	America region	RNeasy Plant Mini Kit (Qiagen)	Column-based	
[53]	2021	125	animal	nasopharyngeal swabs	RNA	illumina	/	Coronavirus	Coronaviridae,	/		QIAamp DNA Mini Kit (Qiagen), QIAamp viral RNA mini kit (Qiagen)	Silica membrane-based	
[54]	2019	2	environment	water	DNA	illumina	/	several	Siphoviridae, Podoviridae, Myoviridae, Adenoviridae, Herpesviridae, Phycodnaviridae, Poxviridae, Mimiviridae, Pandoraviridae,	Peru	America region	ZymoBIOMICS DNA kits (MoBio)	Other/multiple type	pore filtration
[55]	2013	1	animal	fecal samples	DNA	sanger	/	circo-like virus	Circoviridae,	Brazil	America region	QIAamp MinElute Virus Spin Kit (Qiagen)	Column-based	
[56]	2017	177	environment	sewage and reclaimed water samples	DNA	sanger	/	circo-like virus	Circoviridae,	Brazil	America region	High Pure Viral Nucleic Acid kit (Roche)	Filter tube	pore filtration

[57]	2021	120	animal	plasma	DNA	illumina	/	Anellovirus	Anelloviridae,	Spain	European region	QIAamp DNA Mini Kit (Qiagen)	Silica membrane-based
[58]	2015	ND	animal	insects	RNA	illumina	/	narnavirus	Narnaviridae,	California	America region	MasterPure Complete DNA and RNA Purification kit	Magnetic glass particles-based
[59]	2018	1359	animal	insects	RNA	Ion Torrent	De novo	Uxmal virus, Mayapan virus	Unclassified,	Mexico	America region	TRIzol reagents	Solvent-based
[60]	2013	6	animal	fecal samples	DNA	Roche_454	/	porcine stool-associated circular virus	Smacoviridae,	United States	America region	QIAamp MinEluteVirus Vacuum Kit (Qiagen)	Other/multiple type
[61]	2016	540	animal	insects	RNA	Ion Torrent	/	Cuacua virus	Flaviviridae,	Mozambique	Africa region	TRIzol reagents	Solvent-based
[62]	2019	54	animal	fecal samples	DNA and RNA	illumina	De novo	sapelovirus, astroviruses, rotaviruses, picobirnaviruses, parvoviruses, papillomaviruses, polyomaviruses, gammaherpesvirus	Picornaviridae, Astroviridae, Sedoreoviridae, Picobirnaviridae, Parvoviridae, Papillomaviridae, Polyomaviridae, Hepeviridae	Tasmania	South-East Asia region	RNeasy Plus Mini Kit (Qiagen)	Column-based
[63]	2020	24	animal	spleen, liver and bursa tissues	DNA	illumina	De novo	gyrovirus	Anelloviridae,	Brazil	America region	Phenol-chloroform extraction method	Solvent-based
[64]	2021	10	animal	meats	DNA	illumina	/	genomoviruses	Genomoviridae,	Brazil	America region	TRIzol reagents	Solvent-based
[65]	2019	144	plant	leaves	DNA	illumina	/	mastrevirus	Geminiviridae,	Reunion Island	European region	DNeasy Plant DNA extraction kit (Qiagen)	Silica membrane-based
[66]	2014	ND	animal	insects	RNA	illumina	De novo	rhabdovirus, bunyavirus	Rhabdoviridae, Bunyaviridae	Australia	South-East Asia region	QIAamp viral RNA mini kit (Qiagen)	Other/multiple type
[67]	2017	18	animal	fecal samples	DNA and RNA	illumina	/	bocavirus	Parvoviridae,	Portugal	European region	QIAamp viral RNA mini kit (Qiagen)	Other/multiple type
[68]	2016	9	environment	corals	DNA	illumina	De novo	Herpesvirus	Herpesviridae,	Australia	South-East Asia region	Formamide Procedure	Solvent-based

[69]	2019	Database	environment	multiple ecosystems	DNA	Analyzes sequences obtained in other studies	De novo	several	Myoviridae, Podoviridae, Siphoviridae,	/		/	ND	
[70]	2018	37	animal	buccal swab	DNA	illumina	De novo	Anellovirus, Torquo teno virus	Anelloviridae, Anelloviridae	Antartic		High Pure Viral Nucleic Acid kit (Roche)	Filter tube	
[71]	2021	5	plant	leaves	DNA	illumina	/	SiLCV	Geminiviridae,	China	South-East Asia region	/	ND	
[72]	2020	1	environment	water	DNA	illumina	De novo	PA-SR01	Myoviridae,	Singapore	Western Pacific region	QIAamp DNA Mini Kit (Qiagen)	Silica membrane-based	ultracentrifugation
[73]	2021	323	animal	salivary glands	RNA	illumina	De novo	several	Flaviviridae, Partitiviridae, Reoviridae, Phasmaviridae, Rhabdoviridae, Orthomyxoviridae, Picornaviridae,	Brazil	America region	High Pure Viral RNA Kit (Roche)	Filter tube	
[74]	2014	9	animal	lungs, liver and brain	DNA and RNA	illumina	De novo	nairovirus	Bunyaviridae, Orthomyxoviridae	France	European region	RNeasy kit (Qiagen)	Column-based	
[75]	2021	12	animal	blood	DNA	illumina	De novo	circovirus	Circoviridae,	China	South-East Asia region	QIAamp MinElute Virus Spin Kit (Qiagen)	Column-based	
[76]	2021	4	animal	fecal samples	DNA	illumina	De novo	bocaparvovirus	Parvoviridae,	China	South-East Asia region	QIAamp MinElute Virus Spin Kit (Qiagen)	Column-based	
[77]	2021	150	plant	plants	RNA	illumina	De novo	Umbravirus, closterovirus, tritimovirus	Tombusviridae, Closteroviridae, Potyviridae,	France	European region	NucleoSpin kit (Macherey-Nagel)	Silica membrane-based	
[78]	2012	4	plant	leaves	DNA	sanger	/	cassava associated circular DNA virus (CasCV)	Genomoviridae,	England	European region	High Pure Viral Nucleic Acid kit (Roche)	Filter tube	
[79]	2014	nd	animal	larvae	DNA	illumina	/	CRESS-DNA virus	ND,	Ghana	Africa region	Extract-N-Amp (Sigma–Aldrich)	Solvent-based	
[80]	2020	107	plant	leaves	DNA	illumina	/	Begomovirus	Geminiviridae,	Brazil	America region	solvent	Solvent-based	
[81]	2019	93	animal	swab and cloacal samples	RNA	illumina	De novo	picornavirus	Picornaviridae,	Antartic		QIAamp viral RNA mini kit (Qiagen)	Other/multiple type	

[82]	2019	Database	environment	sediments	DNA and RNA	illumina	/	Phages	Fiersviridae, Fiersviridae, Microviridae, Microviridae, Cystoviridae, Siphoviridae	United States	America region	/	ND	fluidic circuit
[83]	2020	102	animal	insects	DNA and RNA	illumina	De novo	several	Siphoviridae, Myoviridae, Podoviridae, Inoviridae, Microviridae, Cystoviridae	/		RNA viral extraction kit (Qiagen)	Other/multiple type	
[84]	2017	17	animal	venous blood, spleen, thymus, and lung	DNA	illumina	De novo	polyomavirus	Polyomaviridae,	California	America region	DNeasy Blood & Tissue Kit (Qiagen), QIAamp DNA FFPE Tissue Kit (Qiagen)	Column-based, Column-based	
[85]	2014	1	environment	surface water	DNA	illumina	De novo	Podovirus, siphovirus	Podoviridae, Siphoviridae	California	America region	/	ND	pore filtration
[86]	2018	Database	animal	Database, ant tissue	DNA	sanger	/	circovirus	Circoviridae,	/		/	ND	
[87]	2016	ND	animal	gut	DNA and RNA	illumina	De novo	chicken megrivirus, sicinivirus 1	Picornaviridae, Picornaviridae	UK	European region	Ribopure RNA extraction kit (Life Technologies)	Other/multiple type	
[88]	2012	1	environment	pore water	DNA and RNA	Roche_454	/	BSL RDHV	Unclassified,	California	America region	Phenol-chloroform extraction method	Solvent-based	tangential flow filtration
[89]	2018	1	animal	skin	DNA	illumina	De novo	adomavirus	Adomaviridae,	/		QIAquick viral RNA column purification system	Column-based	
[90]	2018	1	animal	serum and liver	DNA	illumina	/	parvovirus	Parvoviridae,	Nebraska (USA)	America region	/	ND	
[91]	2015	ND	animal	intestinal content samples	RNA	illumina	De novo	coronavirus	Coronaviridae,	/		High Pure template preparation kit (Roche)	Filter tube	
[92]	2015	ND	animal	mucus samples	DNA and RNA	illumina	/	Porcine astrovirus, sapovirus, Po-Circo-like virus, parvovirus, Rotavirus	Astroviridae, Caliciviridae, Circoviridae, Parvoviridae, Reoviridae,	Europe	European region	Cador Qiagen Pathogen minikit (Hilden)	Column-based	

[93]	2013	2	animal	marine invertebrate species	DNA	sanger	/	circo-like virus	Circoviridae,	Florida	America region	QIAamp MinElute Virus Spin Kit (Qiagen)	Column-based
[94]	2018	35	animal	fecal samples	RNA	illumina	De novo	picobirnaviruses, partitivirus, Picornavirales	Picornaviridae, Picobirnaviridae, Partiviridae,	Congo	Africa region	QIAamp viral RNA mini kit (Qiagen)	Other/multiple type
[95]	2014	Database	animal	fecal samples	DNA	Analyzes sequences obtained in other studies	De novo	CRESS-DNA virus	ND,	/		/	ND
[96]	2019	1	animal	Cerebrospinal fluid and plasma	RNA	illumina	/	orthobunyavirus	Peribunyaviridae,	Uganda	Africa region	Phenol-chloroform extraction method	Solvent-based
[97]	2019	190	animal	cerebrospinal fluid	DNA	illumina	/	betatorquevirus	Anelloviridae,	Ghana	Africa region	MagMAX Viral RNA Isolation kit	Magnetic beads-based
[98]	2018	12	animal	serum	RNA	illumina	De novo	Jingmen tick virus	Flaviviridae,	Kosovo	European region	QIAseq FX DNA Library Kit (Qiagen)	Magnetic beads-based
[99]	2013	ND	animal	bats tissues	DNA and RNA	illumina	/	adenovirus, bocavirus, picobirnavirus, parvovirus	Adenoviridae, Parvoviridae, Picobirnaviridae, Parvoviridae	China	South-East Asia region	/	ND
[100]	2019	9	animal	fecal samples	DNA	illumina	De novo	Chapparvovirus	Parvoviridae,	Colorado	America region	MagMAX Viral RNA Isolation kit	Magnetic beads-based
[101]	2020	781	animal	plasma	DNA	illumina	/	parvovirus and densovirus	Parvoviridae, Parvoviridae	Brazil	America region	MagMAX Viral RNA Isolation kit	Magnetic beads-based
[102]	2020	652	animal	insects	DNA and RNA	illumina	De novo	Flaviviridae, Rhabdoviridae, Totiviridae, and Iflaviridae families	Flaviviridae, Rhabdoviridae, Iflaviridae, Totiviridae	Japan	South-East Asia region	Isogen II (Nippon Gene)	Solvent-based
[103]	2016	464	animal	insects	RNA	illumina	/	flavivirus, BOAV, GAMV, AToV	Flaviviridae, Totiviridae	Senegal	Africa region	Mag-Bind Viral DNA/RNA kit (Omega)	Magnetic beads-based
[104]	2021	23	animal	fecal samples	DNA	Ion Torrent	De novo	CRESS-DNA virus	ND,	Hungary	European region	Direct-zol RNA Miniprep kit (Zymo Research)	Column-based
[105]	2021	ND	plant	plants	DNA	sanger	/	Genomovirus	Genomoviridae,	United States	America region	PureLink Microbiome DNA purification	Column-based

												kit (Invitrogen)		
[106]	2017	28	environment	leaves	RNA	illumina	De novo	Picornaviruses, hepeviruses	Picornaviridae, Hepeviridae	Spain	European region	QIAamp viral RNA mini kit (Qiagen)	Other/multiple type	chemical flocculation
[107]	2019	406	animal	cloacal swabs	RNA	illumina	De novo	astrovirus	Astroviridae,	French Guiana	America region	MagMAX Viral RNA Isolation kit	Magnetic beads-based	
[108]	2020	35	animal	liver, skin, gills	DNA and RNA	illumina	De novo	Betanodaviruses, hepadnaviruses, Rhinovirus A	Nodaviridae, Hepadnaviridae, Picornaviridae,	Portugal	European region	NZYol (NZYTech)	Solvent-based	
[109]	2015	ND	plant	plants	RNA	sanger	/	Potyvirus	Potyviridae,	France	European region	RNeasy Plant Mini Kit (Qiagen)	Column-based	
[110]	2018	214	plant	leaves	DNA and RNA	Roche_454	De novo	Chrysovirus and Umbravirus.	Chrysoviridae, Tombusviridae	Florida	America region	NucleoSpin 96 Virus Core Kit (Macherey-Nagel)	Silica membrane-based	
[111]	2021	36	animal	fecal samples	DNA	illumina	/	Siphoviridae, Myoviridae, Podoviridae, crAss-like viruses	Siphoviridae, Myoviridae, Podoviridae, /	Ireland	European region	/	ND	
[112]	2018	20	plant	leaves	DNA	illumina	De novo	mastrevirus	Geminiviridae,	Brazil	America region	ZR Viral DNA/RNA Kit (Zymo Research)	Other/multiple type	
[113]	2019	2	animal	fecal samples	DNA	illumina	De novo	cyclovirus	Circoviridae,	Brazil	America region	ZR Viral DNA/RNA Kit (Zymo Research)	Other/multiple type	
[114]	2021	13	environment	sediments	DNA	illumina	/	Haloviruses	Unclassified,	Spain	European region	/	ND	
[115]	2019	200	animal	insects	DNA	illumina	De novo	densovirus	Parvoviridae,	France, Greece, Netherlands, Belgium and Portugal	European region	NucleoSpin 96 Virus Core Kit (Macherey-Nagel)	Silica membrane-based	
[116]	2021	16	animal	insects	RNA	illumina	De novo	HpaAV, HpaAV1, HpaAV2	Alphaflexiviridae, Iflaviridae, Iflaviridae,	France	European region	Wizard DNA purification kit (Promega)	Solvent-based	
[117]	2016	378	animal	insects	RNA	illumina	De novo	several	Bunyaviridae, Orthomyxoviridae, Dicistroviridae,	United States	America region	QIAamp viral RNA mini kit (Qiagen)	Other/multiple type	

[118]	2021	12	animal	oral, throat, and anal swab samples	RNA	illumina	/	Picornavirus	Picornaviridae,	China	South-East Asia region	QIAamp viral RNA mini kit (Qiagen)	Other/multiple type	
[119]	2017	1	animal	fecal samples	RNA	illumina	De novo	Camula virus	Unclassified,	/		/	ND	
[120]	2018	37	animal	insects	DNA and RNA	illumina	De novo	Dicistro-like virus, Seco-like virus, Noda-like virus, Tymo-like virus, ARV-1, Partiti-like virus, Circo-like virus	Dicistroviridae, Secoviridae, Nodaviridae, Tymoviridae, Rhabdoviridae, Partitiviridae, Circoviridae,	Several		MagMAX Viral Isolation Kit (Thermo-Fisher)	Magnetic beads-based	
[121]	2018	ND	plant	plants	DNA	sanger	De novo	nanoviruses	Nanoviridae,	France	European region	DNeasy Plant Mini Kit (Qiagen)	Column-based	
[122]	2013	4	environment	water	DNA	illumina	De novo	myoviruses	Myoviridae,	China	South-East Asia region	QIAamp viral RNA kit (Qiagen)	Silica membrane-based	ultracentrifugation, tangential flow filtration
[123]	2021	230	animal	Dead fish	DNA and RNA	illumina	De novo	several	Hantaviridae, Paramyxoviridae, Arenaviridae, Flaviviridae, Filoviridae, Hepadnaviridae, Rhabdoviridae, Matonaviridae, Picornaviridae, Reoviridae, Astroviridae,	Australia	South-East Asia region	RNeasy Plus Mini Kit (Qiagen)	Column-based	
[124]	2016	10	environment	surface water	DNA	illumina	/	DSLVs	Phycodnaviridae,	China	South-East Asia region	QIAamp Fast DNA Stool Mini Kit (Qiagen)	Silica membrane-based	pore filtration
[125]	2017	42	animal	serum	DNA	illumina	De novo	anellovirus	Anelloviridae,	Spain	European region	QIAamp viral RNA mini kit (Qiagen)	Other/multiple type	
[126]	2018	3	environment	microbial mats	DNA	illumina	/	T7-related virus	Podoviridae,	Patagonia	America region	/	ND	tangential flow filtration
[127]	2020	1	environment	raw sewage	RNA	illumina	/	Picobirnaviridae	Picobirnaviridae,	Chile	America region	High Pure Viral RNA Kit (Roche)	Filter tube	ultrafiltration, ultracentrifugation
[128]	2020	Database	environment	wastewater	DNA	illumina	/	virophage	ND,	New York	America region	PowerSoil DNA Isolation kit (MO BIO)	Silica membrane-based	

[129]	2017	37	animal	fecal samples	DNA	illumina	De novo	Microviridae	Microviridae,	China	South-East Asia region	QIAamp viral RNA mini kit (Qiagen)	Other/multiple type	
[130]	2021	696	animal	blood and fecal samples	DNA	illumina	De novo	Anellovirus	Anelloviridae,	China	South-East Asia region	/	ND	
[131]	2015	5	animal	chicken proventriculus	RNA	illumina	/	picornavirus	Picornaviridae,	South Korea	South-East Asia region	Viral Gene-spin Viral DNA/RNA Extraction Kit (iNtRON Biotechnology)	Column-based	
[132]	2019	1795	animal	fecal samples, blood, oral secretion, nasal secretion, genital secretions, skin swab, water	DNA	illumina	De novo	Microviridie	Microviridae,	China	South-East Asia region	QIAamp MinElute Virus Spin Kit (Qiagen)	Column-based	
[133]	2020	26	environment	wastewater	RNA	illumina	De novo	Epatitis E virus	Hepeviridae,	Sweden	European region	DNeasy Blood & Tissue Kit (Qiagen)	Column-based	chemical flocculation
[134]	2016	1	animal	pericardial fluid	DNA	Roche_454	/	gemycircularviruses	Genomoviridae,	France	European region	/	ND	
[135]	2014	4	animal	fecal samples	RNA	illumina	De novo	alphacoronavirus	Coronaviridae,	New Zealand	South-East Asia region	iPrep PureLink Virus Kit (Life Technologies)	Other/multiple type	
[136]	2020	91	animal	fecal samples	RNA	illumina	De novo	Picornavirales	Picornaviridae,	China	South-East Asia region	QIAamp viral RNA mini kit (Qiagen)	Other/multiple type	
[137]	2015	10	animal	fecal samples	DNA	illumina	/	papillomavirus, CRESS-DNA virus	Papillomaviridae,	Malaysia	South-East Asia region	QIAamp DNA Stool kit (Qiagen), QIAamp DNA Mini and Blood kit (Qiagen)	Silica membrane-based, Silica membrane-based	
[138]	2016	36	animal	fecal samples	DNA	illumina	De novo	parvovirus	Parvoviridae,	Hungary	European region	QIAamp spin-column (Qiagen)	Column-based	

[139]	2021	11	animal	fecal samples	DNA	illumina	De novo	Parvovirus	Parvoviridae,	Hungary	European region	QIAamp viral RNA mini kit (Qiagen)	Other/multiple type	
[140]	2021	75	animal	fecal samples	RNA	illumina	/	Picornavirus	Picornaviridae,	Hungary	European region	MagMAX Viral RNA Isolation kit	Magnetic beads-based	
[141]	2019	146	animal	insects	RNA	illumina	De novo	several	Chuviridae, Orthomyxoviridae, Picornaviridae, Flaviviridae, Narnaviridae, Luteoviridae, Virgaviridae, Reoviridae, Phenuiviridae, Partitiviridae	New South Wales	America region	RNeasy Plus Mini Kit (Qiagen)	Column-based	
[142]	2021	76	animal	fecal samples	RNA	Ion Torrent	De novo	Picornavirus	Picornaviridae,	Chile	America region	TRI reagent (Sigma-Aldrich)	Solvent-based	
[143]	2013	853	animal	thorax and abdomen	DNA and RNA	illumina	De novo	Mamastrovirus, Bocavirus, Circovirus, Iflavirus and Orthohepadnavirus	Astroviridae, Circoviridae, Parvoviridae, Flaviviridae, Iflaviridae,	China	South-East Asia region	TRIzol reagents	Solvent-based	
[144]	2013	78	animal	guts and lungs	RNA	illumina	/	rotavirus	Reoviridae,	China	South-East Asia region	RNeasy Mini Kit (Qiagen)	Silica membrane-based	
[145]	2018	39	plant	leaves	RNA	illumina	De novo	GRSPaV	Betaflexiviridae,	France	European region	RNeasy Plant Mini Kit (Qiagen)	Column-based	
[146]	2016	1	environment	hot spring samples	DNA	illumina	/	Acidianus tailed spindle virus (ATSV	Bicaudaviridae,	Australia	South-East Asia region	PureLink Viral DNA and RNA extraction kit (Invitrogen)	Column-based	ultracentrifugation
[147]	2014	579	animal	fecal samples	DNA and RNA	Roche_454	/	adenoviridae and Picornaviridae	Adenoviridae, Picornaviridae	Australia	South-East Asia region	Ampliprep DNA extraction machine (Roche)	Magnetic beads-based	
[148]	2018	90	animal	fecal samples	DNA	illumina	De novo	adenovirus	Adenoviridae,	Bangladesh	Western Pacific region	/	ND	
[149]	2021	5	animal	plasma	DNA	illumina	/	Circovirus	Circoviridae,	Kentucky	America region	/	ND	

[150]	2020	1	animal	heart, liver, spleen, lung, and kidney	DNA	Not available	De novo	bocaparvovirus	Parvoviridae,	China	South-East Asia region	QIAamp MinElute Virus Spin Kit (Qiagen)	Column-based	
[151]	2021	5	animal	blood	DNA	illumina	De novo	Anellovirus	Anelloviridae,	China	South-East Asia region	QIAamp viral RNA mini kit (Qiagen)	Other/multiple type	
[152]	2016	ND	animal	marine invertebrate species	DNA	illumina	/	CRESS-DNA virus	ND,	Several		/	ND	
[153]	2020	6	animal	pyloric caeca and body wall tissue	DNA	illumina	De novo	AfaDV	Parvoviridae,	Atlantic Ocean		Viral DNA kit (Zymo Research)	Column-based	
[154]	2019	6	plant	Mangrove soil samples	DNA	illumina	De novo	Caudovirales phage	Podoviridae,	China	South-East Asia region	/	ND	
[155]	2020	3	plant	fruits	RNA	illumina	De novo	Monilinia umbra-like virus 1 (MULV1)	Tombusviridae,	South Korea	South-East Asia region	DNeasy Plant Mini Kit (Qiagen), RNeasy Plant Mini Kit (Qiagen)	Column-based, Column-based	
[156]	2019	3	environment	Groundwater	DNA	illumina	/	Myoviridae and Siphoviridae	Siphoviridae, Myoviridae	Germany	European region	/	ND	ultrafiltration, ultracentrifugation
[157]	2015	87	animal	blood	RNA	illumina	De novo	Simian pegivirus, simian anelloviruses	Flaviviridae, Anelloviridae	Several		Maxwell 16 viral total nucleic acid purification kit (Promega)	Magnetic beads-based	
[158]	2019	48	animal	fecal samples	RNA	illumina	/	sapovirus	Caliciviridae,	Japan	South-East Asia region	TRIzol reagents	Solvent-based	
[159]	2019	164	animal	fecal samples	RNA	illumina	/	astrovirus	Astroviridae,	Switzerland	European region	QIAamp viral RNA mini kit (Qiagen)	Other/multiple type	
[160]	2015	ND	animal	fecal samples	DNA	Ion Torrent	/	Parvovirus	Parvoviridae,	Hungary	European region	GeneJet Viral DNA and RNA Purification Kit (Thermo Fisher)	Silica membrane-based	
[161]	2017	105	plant	leaves	RNA	illumina	De novo	Ilarvirus-like	Bromoviridae,	Australia	South-East Asia region	RNeasy Plant Mini Kit (Qiagen)	Column-based	

[162]	2018	ND	animal	fecal samples	RNA	Roche_454	De novo	calicivirus	Caliciviridae,	United States	America region	/	ND
[163]	2021	ND	environment	water	DNA	illumina	De novo	several	Microviridae, Inoviridae	Tennessee	America region	/	ND
[164]	2017	1351	animal	blood	DNA	illumina	De novo	torquo teno virus	Anelloviridae,	/		QIAamp Circulating Nucleic Acid Kit (Qiagen)	Silica membrane-based
[165]	2019	40	animal	brain and hemolymph	DNA	illumina	De novo	genomoviruses and microvirus	Microviridae, Genomoviridae	Arizona	America region	High Pure Viral Nucleic Acid kit (Roche)	Filter tube
[166]	2019	40	animal	insects	DNA	illumina	/	genomoviruses, microvirus, circovirus	Microviridae, Genomoviridae, Circoviridae,	New Zealand	South-East Asia region	High Pure Viral Nucleic Acid kit (Roche)	Filter tube
[167]	2019	ND	animal	intestinal sample	DNA	illumina	De novo	bocaparvovirus	Parvoviridae,	United States	America region	/	ND
[168]	2010	5	animal	fecal samples	DNA and RNA	Roche_454	/	several	Circoviridae, Picornaviridae, Dicistroviridae,	California	America region	QIAamp viral RNA mini kit (Qiagen)	Other/multiple type
[169]	2013	32	animal	brain sample	RNA	illumina	De novo	BoAstV-NeuroS1	Astroviridae,	California	America region	Agencourt Formapure Kit	Magnetic beads-based
[170]	2015	34	animal	plasma, nasal swab, fecal samples	DNA and RNA	illumina	De novo	parvovirus, cyclovirus, kirkovirus	Parvoviridae, Circoviridae, Circoviridae,	United States	America region	QIAamp viral RNA mini kit (Qiagen), MagMAX Viral RNA Isolation kit	Other/multiple type, Magnetic beads-based
[171]	2011	18	animal	fecal samples	RNA	Roche_454	De novo	kobuvirus and sapovirus	Caliciviridae, Picornaviridae	California	America region	QIAamp viral RNA mini kit (Qiagen)	Other/multiple type
[172]	2011	10	animal	spleen, heart and lung tissues	DNA	sanger	/	amdovirus	Parvoviridae,	California	America region	/	ND
[173]	2017	368	animal	nasopharyngeal aspirates	RNA	illumina	/	HPIV 3	Paramyxoviridae,	China	South-East Asia region	NucliSens easyMAG system (bioMérieux)	Other/multiple type

[174]	2021	ND	animal	insects	RNA	illumina	De novo	Negevirus	Unclassified,	Kenya	Africa region	QIAamp viral RNA mini kit (Qiagen)	Other/multiple type
[175]	2013	7	animal	plasma	DNA	illumina	De novo	circovirus	Circoviridae,	Canada	America region	QIAamp viral RNA mini kit (Qiagen)	Other/multiple type
[176]	2021	7	animal	fecal samples	RNA	illumina	De novo	alphacoronavirus	Coronaviridae,	Denmark	European region	MagNA Pure 96 system (Roche)	Magnetic beads-based
[177]	2018	ND	animal	insects	DNA	illumina	De novo	NV11X, NG24X	ND,	/		QIAamp MinElute Virus Spin Kit (Qiagen)	Column-based
[178]	2019	48	animal	insects	RNA	illumina	De novo	VOV1, VDV4	Orthomyxoviridae, Orthomyxoviridae	Thailand	Western Pacific region	TRI reagent (Sigma-Aldrich)	Solvent-based
[179]	2014	15	animal	intestinal content samples	RNA	illumina	/	coronavirus	Coronaviridae,	France	European region	/	ND
[180]	2019	70	animal	fecal samples	DNA	illumina	De novo	Calcivirus, cress-DNA virus	Caliciviridae, /	Brazil	America region	TRIzol reagents, phenol-chloroform extraction method	Solvent-based, Solvent-based
[181]	2019	80	animal	genital tract secretion	DNA	illumina	De novo	papillomavirus	Papillomaviridae,	China	South-East Asia region	QIAamp MinElute Virus Spin Kit (Qiagen)	Column-based
[182]	2016	88	animal	vaginal swab	DNA	illumina	/	papillomavirus	Papillomaviridae,	China	South-East Asia region	QIAamp viral RNA mini kit (Qiagen)	Other/multiple type
[183]	2019	10	animal	fecal samples	DNA	illumina	De novo	Smacovirus	Smacoviridae,	Brazil	America region	High Pure Viral Nucleic Acid kit (Roche)	Filter tube
[184]	2018	145	animal	fecal samples	DNA	Analyzes sequences obtained in other studies	/	CRESS-DNA virus	ND,	China	South-East Asia region	/	ND
[185]	2019	1200	plant	plants	RNA	illumina	De novo	Several	Endornaviridae,	France	European region	/	ND
[186]	2020	170	plant	leaves	RNA	illumina	De novo	Ilarvirus	Bromoviridae,	/		/	ND

[187]	2019	20	animal	fecal samples	RNA	illumina	/	cardiovirus	Picornaviridae,	China	South-East Asia region	QIAamp viral RNA mini kit (Qiagen)	Other/multiple type	
[188]	2019	1657	animal	salivary glands	RNA	illumina	De novo	Pirizal iflavirus, Furrundu phlebovirus, Pixé phlebovirus, Guampa vesiculovirus, Chacororé flavivirus, Rasqueado orbivirus, Uru chuvirus, Bororo circovirus	Iflaviridae, Flaviviridae, Phenuiviridae, Rhabdoviridae, Chuviridae, Reoviridae, Circoviridae,	Brazil	America region	High Pure Viral RNA Kit (Roche)	Filter tube	
[189]	2014	5	animal	fecal samples	RNA	illumina	De novo	rotavirus	Reoviridae,	Japan	South-East Asia region	ISGEN LS (NipponGene)	Solvent-based	
[190]	2018	107	animal	fecal samples	RNA	illumina	/	picornavirus	Picornaviridae,	Japan	South-East Asia region	/	ND	
[191]	2015	63	animal	fecal samples	RNA	Ion Torrent	De novo	rotavirus	Reoviridae,	Hungary	European region	/	ND	
[192]	2013	1	animal	fecal samples	DNA	illumina	/	microviridae	Microviridae,	Pennsylvania	America region	QIAamp DNA Stool kit (Qiagen)	Silica membrane-based	
[193]	2016	6	environment	seawater	RNA	Roche_454	/	picornavirus	Picornaviridae,	Antartic		MasterPure Complete DNA and RNA Purification kit	Solvent-based	tangential flow filtration
[194]	2021	10	animal	bronchoalveolar lavage	RNA	illumina	/	Rhinovirus	Picornaviridae,	Australia	South-East Asia region	/	ND	
[195]	2013	1	environment	Mediterranean deep chlorophyll maximum	DNA	illumina	De novo	Phages	Podoviridae,	Spain	European region	Phenol-chloroform extraction method	Solvent-based	pore filtration
[196]	2016	12	environment	seawater	DNA	illumina	De novo	UvDeep viruses	Phycodnaviridae, Iridoviridae	Mediterranean Sea	European region	QIAprep Spin Miniprep kit (Qiagen)	Column-based	pore filtration
[197]	2017	1	animal	cutaneous malignant melanoma	DNA	illumina	De novo	cutavirus	Parvoviridae,	Brazil	American region	/	ND	

[198]	2020	3	environment	soil and water	DNA	illumina	/	Methanosarcina virus MV (MetMV)	Unclassified,	Hungary	European region	PowerSoil Max kit (MO BIO)	Silica membrane-based	
[199]	2016	2236	animal	insects	RNA	illumina	De novo	Nairovirus and Phlebovirus	Bunyaviridae, Bunyaviridae	France	European region	Nucleospin RNA II kit (Macherey-Nagel)	Silica membrane-based	
[200]	2014	153	environment	planktonic organisms	DNA	Roche_454	/	mimiviridae	Mimiviridae,	/		/	ND	pore filtration
[201]	2019	6	animal	fecal samples	DNA	illumina	De novo	crane-associated adenovirus 1 (CrAdV-1)	Adenoviridae,	Japan	South-East Asia region	QIAamp DNA Stool kit (Qiagen)	Silica membrane-based	
[202]	2016	Database	plant	non-angiosperm green plant	RNA	Analyzes sequences obtained in other studies	/	alphapartitivirus	Partitiviridae,	/		/	ND	
[203]	2015	50	animal	fecal samples	RNA	illumina	De novo	picornavirus	Picornaviridae,	Japan	South-East Asia region	TRIzol reagents	Solvent-based	
[204]	2016	1	animal	fecal samples	RNA	illumina	De novo	picornavirus	Picornaviridae,	Japan	South-East Asia region	TRIzol reagents	Solvent-based	
[205]	2021	4265	animal	insects	DNA and RNA	illumina	/	several	Rhabdoviridae, Totiviridae, Iflaviviridae, Circoviridae, Sobemoviridae,	France	European region	High pure viral nucleic acid large volume kit (Roche)	Column-based	
[206]	2018	33	plant	leaves	RNA	illumina	De novo	alfalfa virus F	Tymoviridae,	France	European region	TRIzol reagents	Solvent-based	
[207]	2020	60	plant	leaves	RNA	illumina	De novo	HDaV1, HDaV2	Picornaviridae,	Brazil	America region	TRIzol reagents	Solvent-based	
[208]	2017	9	environment	water	DNA	illumina	De novo	Haloviruses, T4-like myoviruses, podoviruses, siphovirus, dwarf myoviruse, Synechococcus phage S-EIV1, Prochlorococcus phage P-RSP2	Unclassified, Myoviridae, Podoviridae, Siphoviridae, Myoviridae, Unclassified, Autographiviridae,	Japan	South-East Asia region	/	ND	FeCl3 precipitation
[209]	2015	ND	animal	insects	DNA	illumina	De novo	Diaphorina citri densovirus	Parvoviridae,	Florida	America region	TRIzol reagents	Solvent-based	

[210]	2018	222	animal	fecal samples	RNA	illumina	De novo	Teschovirus A-related virus	Picornaviridae,	Japan	South-East Asia region	NEBNext Ultra RNA Library Prep Kit for Illumina (NEB)	Magnetic beads-based	
[211]	2016	13	environment	surface water	DNA	Roche_454 and Sanger	/	phycodnaviruses	Phycodnaviridae,	Tibet	South-East Asia region	/	ND	pore filtration
[212]	2019	953	animal	insects	RNA	Ion Torrent	De novo	several	Iflaviridae, Nodaviridae, Orthomyxoviridae, Partitviridae, Peribunyaviridae, Phasmaviridae, Reoviridae, Rhabdoviridae, Solemoviridae, Tombusviridae	Sweden	European region	TRIzol reagents	Solvent-based	
[213]	2018	76	animal	insects	RNA	illumina	De novo	flavivirus	Flaviviridae,	Several		Tungsten carbide beads (Qiagen)	Magnetic beads-based	
[214]	2020	40	animal	fecal samples	DNA	illumina	De novo	genomoviruses and microvirus	Microviridae, Genomoviridae	Arizona	America region	High Pure Viral Nucleic Acid kit (Roche)	Filter tube	
[215]	2021	2	environment	seafloor	DNA	illumina	/	Caudovirales	ND,	Pacific Ocean		PowerSoil DNA Isolation kit (MO BIO)	Silica membrane-based	
[216]	2016	406	plant	leaves	RNA	Roche_454	De novo	polerovirus-like, tombusvirus-like	Luteoviridae, Tombusviridae	Burkina Faso	Africa region	NucleoSpin 96 Virus Core Kit (Macherey-Nagel)	Silica membrane-based	
[217]	2018	311	animal	serum	DNA	illumina	De novo	torquo teno mini virus	Anelloviridae,	China	South-East Asia region	QIAamp viral RNA kit (Qiagen)	Silica membrane-based	
[218]	2015	19	animal	fecal samples	RNA	Roche_454	/	astrovirus	Astroviridae,	Hungary	European region	/	ND	
[219]	2018	1	animal	cloacal and tissue samples	RNA	illumina	De novo	passerivirus	Picornaviridae,	Hungary	European region	QIAamp spin-column (Qiagen)	Column-based	
[220]	2016	1	environment	sewage waters	DNA	illumina	De novo	several	Circoviridae, Geminiviridae, Microviridae,	Florida	America region	Phenol-chloroform extraction method	Solvent-based	ultrafiltration

[221]	2021	7447	animal	insects	RNA	illumina	/	Jingchuvirales and Bunyavirales	Peribunyaviridae,	Korea	South-East Asia region	QIAamp viral RNA mini kit (Qiagen)	Other/multiple type
[222]	2019	4100	animal	insects	RNA	illumina	/	Uukuniemi virus and phlebovirus	Phenuiviridae, Phenuiviridae	Zealand	European region	MagNA Pure Large Volume kit (Roche)	Magnetic glass particles-based
[223]	2017	1	animal	brain, spinal cord and spleen material	RNA	Ion Torrent	De novo	astrovirus	Astroviridae,	UK	European region	RNeasy kit (Qiagen), TRIzol reagents	Column-based, Solvent-based
[224]	2017	ND	environment	soil	DNA	illumina	De novo	phage NCTB	Myoviridae,	New Caledonia	South-East Asia region	/	ND
[225]	2018	88	animal	serum	RNA	illumina	De novo	partitivirus, dicistrovirus	Dicistroviridae, Partiviridae	Peru	America region	Maxwell 16 automated extractor (Promega)	Solvent-based
[226]	2016	58	animal	fecal samples	DNA	illumina	/	pecoviruses	Unclassified,	Peru	America region	Maxwell 16 automated extractor (Promega)	Magnetic beads-based
[227]	2016	5	animal	fecal samples	DNA	illumina	De novo	protoparvovirus	Parvoviridae,	Bhutan	Western Pacific region	Maxwell 16 automated extractor (Promega)	Magnetic beads-based
[228]	2016	3	animal	heart and lung tissues homogenate	DNA	illumina	De novo	PCV3	Circoviridae,	United States	America region	MagMAX-96 Viral RNA Isolation Kit (Ambion)	Magnetic beads-based
[229]	2014	120	animal	nasopharyngeal aspirates	DNA	illumina	/	cyclovirus	Circoviridae,	Chile	America region	MagMAX Viral RNA Isolation kit	Magnetic beads-based
[230]	2014	48	animal	fecal samples	RNA	illumina	/	astrovirus	Astroviridae,	Burkina Faso	Africa region	/	ND
[231]	2020	Database	animal	fecal samples and tissues	RNA	illumina	/	picorna-like virus, noroviruses, toti-like virus, Alongshan virus	Picornaviridae, Caliciviridae, Totiviridae, Flaviviridae	Finland	European region	QIAamp viral RNA kit (Qiagen)	Silica membrane-based
[232]	2013	10	animal	blood	DNA	Roche_454	/	marseillevirus-like	Marseilleviridae,	France	European region	High Pure Viral Nucleic Acid kit (Roche)	Filter tube
[233]	2020	5	animal	Brain, heart, tic, liver	RNA	illumina	/	Hepacivirus, Pegivirus	Flaviviridae, Flaviviridae	Australia	South-East Asia region	RNeasy Plus Mini Kit (Qiagen)	Column-based

[234]	2021	6674	animal	insects	RNA	illumina	De novo	flavivirus and orthomyxovirus	Flaviviridae,	Australia	South-East Asia region	QIAamp viral RNA mini kit (Qiagen)	Other/multip le type	
[235]	2020	85	animal	fecal samples	DNA	illumina	De novo	several	Smacoviridae, Unclassified	China	South-East Asia region	QIAamp viral RNA mini kit (Qiagen)	Other/multip le type	
[236]	2017	1	animal	nasal swabs and fecal samples	DNA	illumina	De novo	polyomavirus	Polyomaviridae,	China	South-East Asia region	QIAamp viral RNA mini kit (Qiagen)	Other/multip le type	
[237]	2017	625	animal	shrimps	DNA	illumina	De novo	SHIV	Iridoviridae,	China	South-East Asia region	QIAamp viral DNA kit (Qiagen)	Column-based	
[238]	2015	12	environment	peat soil water samples	DNA	Roche_454	De novo	microviridae	Microviridae,	France	European region	Nucleospin Extract II kit (Macherey–Nagel)	Silica membrane-based	PEG precipitation
[239]	2016	12	environment	peat soil water samples	DNA	Roche_454	De novo	crucivirus	Cruciviridae,	France	European region	/	ND	PEG precipitation
[240]	2020	ND	animal	mosquito excreta samples	RNA	illumina	De novo	Smithfield permutotetra-like virus, Redbank virus, Old Port virus, Perrin Park virus, Warroolaba Creek virus 1, Warroolaba Creek virus 2, Warroolaba Creek virus 3	Unclassified, Unclassified, Unclassified, Iflaviridae, Dicistroviridae, Dicistroviridae, Dicistroviridae,	Australia	South-East Asia region	QIAamp viral RNA mini kit (Qiagen)	Other/multip le type	
[241]	2021	24	animal	insects	RNA	illumina	De novo	rhabdovirus	Rhabdoviridae,	Mexico	America region	QIAamp viral RNA mini kit (Qiagen)	Other/multip le type	
[242]	2018	3	environment	air	DNA	illumina	/	Caulimoviridae	Caulimoviridae,	Spain	European region	PowerSoil DNA Isolation kit (MO BIO)	Silica membrane-based	
[243]	2017	ND	animal	thorax and abdomen	RNA	illumina	De novo	ADV, ARV, ABV, ANV, AFV	Rhabdoviridae, Bunyaviridae, Flaviviridae, Iflaviridae, Unclassified,	Several		TRIzol reagents	Solvent-based	
[244]	2013	2	animal	intestinal content samples	RNA	Roche_454	/	seadornavirus	Reoviridae,	Hungary	European region	TRIzol reagents	Solvent-based	
[245]	2014	1	animal	fecal samples	RNA	Roche_454	/	dicistrovirus	Dicistroviridae,	Hungary	European region	QIAamp viral RNA mini kit (Qiagen)	Other/multip le type	

[246]	2018	39	animal	fecal samples	RNA	illumina	De novo	hepevirus	Hepeviridae,	Hungary	European region	QIAamp spin-column (Qiagen)	Column-based	
[247]	2020	98	animal	fecal samples	RNA	illumina	De novo	hepevirus	Hepeviridae,	Hungary	European region	MagMAX Viral RNA Isolation kit	Magnetic beads-based	
[248]	2015	24	animal	fecal samples	RNA	illumina	De novo	picornavirus	Picornaviridae,	Hungary	European region	/	ND	
[249]	2015	1	animal	intestinal content samples	RNA	Roche_454	/	posavirus	Smacoviridae,	Hungary	European region	/	ND	
[250]	2020	58	animal	mussels	DNA	illumina	De novo	densovirus	Parvoviridae,	United States	America region	QIAamp MinElute Virus Spin Kit (Qiagen)	Column-based	
[251]	2019	ND	plant	plants	DNA	sanger	De novo	CRESS-DNA virus	Unclassified,	New Zealand	South-East Asia region	/	ND	
[252]	2020	1	animal	cerebral biopsy	RNA	illumina	De novo	cristoli virus	peribunyaviridae,	France	European region	QIAsymphony (Qiagen)	Other/multiple type	
[253]	2018	Database	animal	kidney	DNA	illumina	De novo	parvovirus	Parvoviridae,	Australia	South-East Asia region	RNeasy Mini Kit (Qiagen)	Silica membrane-based	
[254]	2010	384	plant	plants	RNA	Roche_454	/	several	Bromoviridae, Caulimoviridae, Chrysoviridae, Closteroviridae, Endornaviridae, Luteoviridae, Narnaviridae, Partitiviridae, Potyviridae, Totiviridae, Tymoviridae, Unclassified	Costa Rica	America region	Phenol-chloroform extraction method	Solvent-based	
[255]	2009	Database	environment	reclaimed and sea water	DNA	Analyzes sequences obtained in other studies	/	circovirus	Circoviridae,	Several		/	ND	tangential flow filtration
[256]	2011	ND	animal	insects	DNA	Not available	/	cyclovirus	Circoviridae,	New Zealand	South-East Asia region	QIAamp MinElute Virus Spin Kit (Qiagen)	Column-based	
[257]	2013	6	animal	insects	DNA	Not available	/	mastrevirus	Geminiviridae,	United States	America region	QIAamp MinElute Virus Spin Kit (Qiagen)	Column-based	

[258]	2015	21	animal	marine invertebrate species	DNA	Roche_454	/	CRESS-DNA virus	ND,	/		QIAamp MinElute Virus Spin Kit (Qiagen)	Column-based	
[259]	2015	ND	animal	insects	DNA	Roche_454	/	Begomovirus	Geminiviridae,	Several		QIAamp MinElute Virus Spin Kit (Qiagen)	Column-based	
[260]	2012	Database	environment	water	DNA	Roche_454	/	Chlamydiae phage	Microviridae,	France	European region	QIAamp DNA Mini Kit (Qiagen)	Silica membrane-based	tangential flow filtration, PEG precipitation
[261]	2014	127	environment	water	DNA	illumina	/	microviridae and caudovirales	Microviridae, Podoviridae	Canada	America region	Wizard Prep kit (Promega)	Column-based	pore filtration
[262]	2017	2	environment	freshwater	DNA	illumina	De novo	several	Mimiviridae,	Wisconsin	America region	FastDNA Kit (MP Biomedicals)	Silica membrane-based	pore filtration
[263]	2021	384	plant	fungus	DNA	illumina	De novo	narnavirus	Narnaviridae,	Italy and Spain	European region	Spectrum Plant Total RNA kit instructions (Sigma-Aldrich)	Column-based	
[264]	2020	1	animal	C6/36 cells (Aedes albopictus)	RNA	Ion Torrent	De novo	arbovirus	Flaviviridae,	Brazil	America region	QIAamp viral RNA mini kit (Qiagen)	Other/multiple type	
[265]	2012	10	animal	plasma	DNA	illumina	/	HERV	Retroviridae,	China	South-East Asia region	QIAamp DNA blood mini kit (Qiagen)	Silica membrane-based	
[266]	2016	40	animal	fecal samples	DNA	illumina	De novo	Chapparvovirus	Parvoviridae,	China	South-East Asia region	MiniBEST Universal Genomic DNA Extraction Kit (TaKaRa)	Magnetic beads-based	
[267]	2016	40	animal	intestinal content samples	DNA	illumina	/	RatBuV	Parvoviridae,	China	South-East Asia region	MiniBEST Universal Genomic DNA Extraction Kit (TaKaRa)	Column-based	
[268]	2016	20	animal	fecal samples	DNA	illumina	De novo	bocavirus	Parvoviridae,	China	South-East Asia region	MiniBEST Universal Genomic DNA Extraction Kit (TaKaRa)	Column-based	

[269]	2021	120	plant	fruits	RNA	illumina	De novo	tepovirus	Betaflexiviridae,	Florida	America region	/	ND
[270]	2018	12290	animal	insects	DNA and RNA	illumina	De novo	several	Luteoviridae, Tombusviridae, Tymoviridae, Virgaviridae, Astroviridae, Mesoniviridae, Nodaviridae, Unclassified, Hytrosaviridae, Anelloviridae, Bidnaviridae, Circoviridae, Parvoviridae,	California	America region	MagMAX Express-96 Deep Well Magnetic Particle Processor	Magnetic beads-based
[271]	2017	4	animal	serum	DNA	illumina	/	parvovirus	Parvoviridae,	United States	America region	MagMAX Viral RNA Isolation kit	Magnetic beads-based
[272]	2017	ND	animal	insects	RNA	illumina	De novo	Tetravirus, reoviruses, orbivirus,	Alphatetraviridae, Reoviridae, Reoviridae,	Texas	America region	MagMAX Viral RNA Isolation kit	Magnetic beads-based
[273]	2019	6368	animal	insects	RNA	illumina	De novo	YBV1, YPLV3, YSLV1	Bunyaviridae, Picornaviridae, Unclassified,	Oklahoma	America region	QIAamp 96 Virus QIAcube HT kit (Qiagen)	Silica membrane-based
[274]	2016	155	animal	fecal samples	RNA	illumina	De novo	Posavirus	Picornaviridae,	Japan	South-East Asia region	TRIzol reagents	Solvent-based
[275]	2021	140	animal	cell culture	RNA	illumina	/	Hedwig virus	Peribunyaviridae,	Germany	European region	QIAamp viral RNA mini kit (Qiagen)	Other/multiple type
[276]	2015	72	animal	lung, liver, spleen, kidney and intestinal contents	RNA	Ion Torrent	/	cyclovirus	Circoviridae,	Zambia	Africa region	High Pure Viral Nucleic Acid kit (Roche)	Filter tube
[277]	2013	9	plant	plants	RNA	Roche_454	/	panicovirus	Tombusviridae,	Oklahoma	America region	/	ND
[278]	2016	1	animal	brain sample	RNA	illumina	/	astrovirus	Astroviridae,	Germany	European region	TRIzol reagents, RNeasy Mini Kit (Qiagen)	Solvent-based, Silica membrane-based
[279]	2016	ND	animal	insects	RNA	Ion Torrent	De novo	SRBV, GABV	Aliusviridae, Peribunyaviridae	Belgium	European region	RNeasy Lipid Tissue Mini kit (Qiagen)	Column-based

[280]	2018	28	environment	soil	DNA and RNA	illumina	/	mimiviridae	Mimiviridae,	Massachusetts	America region	PowerSoil DNA Isolation kit (MO BIO), PowerSoil RNA Isolation kit (MO BIO)	Silica membrane-based, Column-based	syringe filtratione
[281]	2014	39	animal	swab samples	RNA	Roche_454	De novo	parvovirus, polyomavirus	Parvoviridae, Polyomaviridae	Arctic		/	ND	
[282]	2017	261521	animal	insects	RNA	illumina	De novo	rhabdovirus	Rhabdoviridae,	Germany	European region	MagMAX Viral RNA Isolation kit	Magnetic beads-based	
[283]	2011	12	animal	fecal samples	RNA	Roche_454	/	astroviruses and bocaviruses	Astroviridae, Parvoviridae	North Carolina	America region	QIAamp viral RNA kit (Qiagen)	Silica membrane-based	
[284]	2017	1	animal	fecal samples	RNA	illumina	De novo	Enterovirus	Picornaviridae,	California	America region	SV Total Viral RNA mini kit (Qiagen)	Column-based	
[285]	2019	95	animal	insects	RNA	illumina	De novo	several	Luteoviridae, Phenuiviridae, Totiviridae, Unclassified, Orthomyxoviridae, Rhabdoviridae, Tymoviridae,	Guadeloupe	America region	QIAamp viral RNA mini kit (Qiagen)	Other/multiple type	
[286]	2019	ND	animal	fecal samples	DNA	illumina	De novo	circo-like virus	Circoviridae,	Croatia	European region	iPrep virus kit (Invitrogen)	Other/multiple type	
[287]	2020	15	animal	guano, feces and saliva	DNA	illumina	De novo	Adeno-associated virus, densovirus and iflavirus	Adenoviridae, Iflaviridae, Parvoviridae,	Croatia	European region	iPrep virus kit (Invitrogen)	Other/multiple type	
[288]	2021	2142	animal	fecal samples	RNA	illumina	De novo	Rotavirus	Reoviridae,	Ghana, Gabon, Bulgaria, Romania, Germany, and Costa Rica	Multiple (Africa, Europe and America)	QIAamp viral RNA mini kit (Qiagen)	Other/multiple type	
[289]	2017	69	animal	tissue pools	DNA	illumina	De novo	Parvovirus, polyomavirus, adenovirus	Parvoviridae, Polyomaviridae, Adenoviridae,	California	America region	QIAamp viral RNA mini kit (Qiagen)	Other/multiple type	
[290]	2014	13	animal	fecal samples	DNA	Roche_454	/	circular DNA viruses	Circoviridae,	Netherlands	European region	/	ND	

[291]	2013	39	animal	fecal samples	DNA and RNA	sanger	/	Picornavirus, papillomavirus, anellovirus	Picornaviridae, Papillomaviridae, Anelloviridae,	Netherlands	European region	/	ND	
[292]	2013	58	animal	Serum and CSF	DNA	Roche_454	De novo	cyclovirus	Circoviridae,	Malawi	Africa region	MagNA Pure LC - Total Nucleic Acid Kit (Roche)	Magnetic glass particles-based	
[293]	2021	107	animal	nasopharyngeal swabs	RNA	illumina	/	orthopneumovirus	Pneumoviridae,	China	South-East Asia region	/	ND	
[294]	2018	36	animal	ticks, bovine serum	RNA	illumina	De novo	tymovirus	Tymoviridae,	Brazil	America region	QIAamp viral RNA mini kit (Qiagen)	Other/multiple type	
[295]	2021	10	environment	water	DNA	illumina	De novo	several	Siphoviridae, Myoviridae, Phycodnaviridae,	Atlantic Ocean		Phenol-chloroform extraction method	Solvent-based	FeCl3 precipitation
[296]	2017	12	plant	leaves	DNA	illumina	De novo	capulavirus	Geminiviridae,	Finland	European region	TRIzol reagents	Solvent-based	
[297]	2019	254	plant	leaves	RNA	illumina	De novo	Caulimovirus, Betapartitivirus, Enamovirus, Closterovirus	Caulimoviridae, Partiviridae, Solemoviridae, Closteroviridae	Finland	European region	E.Z.N.A. Plant Kit (Omega Biotek)	Magnetic beads-based	
[298]	2019	12	animal	fecal samples	DNA	illumina	De novo	CRESS-DNA virus	ND,	Japan	South-East Asia region	AMPure beads (PacBio)	Magnetic beads-based	
[299]	2011	13	animal	lung samples	DNA	sanger	/	anellovirus	Anelloviridae,	California	America region	QIAamp MinElute Virus Spin Kit (Qiagen)	Column-based	
[300]	2017	20	animal	brain tumor samples	DNA	illumina	De novo	betatorquevirus	Anelloviridae,	/		QIAamp viral RNA mini kit (Qiagen)	Other/multiple type	
[301]	2009	62	animal	external swabs	DNA	sanger	/	tornovirus 1 (STTV1)	Circoviridae,	Florida	America region	QIAamp MinElute Virus Spin Kit (Qiagen)	Column-based	
[302]	2012	ND	environment	sewage waters	DNA and RNA	Roche_454	/	kobuvirus, salivirus, sapovirus, astrovirus	Picornaviridae, Picornaviridae, Caliciviridae, Astroviridae	Several		QIAamp spin columns (Qiagen)	Column-based	tangential flow filtration
[303]	2014	55	animal	fecal samples	RNA	illumina	De novo	Sakobuvirus, feline bocavirus 2	Picornaviridae, Parvoviridae	Portugal	European region	QIAamp viral RNA mini kit (Qiagen)	Other/multiple type	

[304]	2015	50	animal	sea otters	DNA	illumina	/	papillomavirus	Papillomaviridae,	California	America region	/	ND	
[305]	2013	12	animal	Hepatopancreas tissue	DNA	sanger	/	nodavirus	Nodaviridae,	Florida	America region	DNeasy Blood & Tissue Kit (Qiagen)	Column-based	
[306]	2011	480	animal	insects	DNA	Roche_454	De novo	densovirus	Parvoviridae,	California	America region	Formamide Procedure	Solvent-based	
[307]	2013	ND	animal	fecal samples and tissues	RNA	Roche_454	/	astrovirus	Astroviridae,	Japan	South-East Asia region	QIAamp MinElute Virus Spin Kit (Qiagen)	Column-based	
[308]	2011	2	plant	insects	DNA	sanger	/	Begomovirus	Geminiviridae,	Florida	America region	QIAamp MinElute Virus Spin Kit (Qiagen)	Column-based	
[309]	2019	70	animal	blood	RNA	illumina	De novo	Zaire Ebola virus (EBOV)	Filoviridae,	Several		QIAamp viral RNA kit (Qiagen)	Silica membrane-based	
[310]	2020	573	animal	insects	RNA	illumina	De novo	Chuviridae, Phenuiviridae	Chuviridae, Phenuiviridae	China	South-East Asia region	QIAamp viral RNA mini kit (Qiagen)	Other/multiple type	
[311]	2020	251	animal	fecal samples	DNA	illumina	De novo	Adenovirus C	Adenoviridae,	Brazil	America region	ZR Viral DNA/RNA Kit (Zymo Research)	Other/multiple type	
[312]	2012	1	environment	plants	DNA	Roche_454	/	Bordetella phages bpp1	Podoviridae,	Singapore	Western Pacific region	Formamide Procedure	Solvent-based	tangential flow filtration
[313]	2019	ND	animal	insects	RNA	Ion Torrent	/	orbivirus	Reoviridae,	United States	America region	TRIzol reagents	Solvent-based	
[314]	2016	315	animal	insects	RNA	illumina	/	Thogotovirus, Rhabdovirus	Rhabdoviridae,	Senegal	Africa region	TRIzol reagents	Solvent-based	
[315]	2021	27	animal	insects	RNA	illumina	De novo	zika virus	Flaviviridae,	Barbados	America region	QIAamp UCP Micro Kit (Qiagen)	Column-based	
[316]	2016	1	animal	fecal samples	RNA	illumina	De novo	Rotavirus astrovirus enterovirus	Astroviridae, Reoviridae, Picornaviridae,	Belgium	European region	QIAamp viral RNA mini kit (Qiagen)	Other/multiple type	
[317]	2020	91	animal	nasal-throat swabs	DNA and RNA	illumina	De novo	Cyclovirus, gemycircularvirus, and statovirus	Circoviridae,	California	America region	QIAamp 96 Virus	Silica membrane-based	

												QIAcube HT Kit (Qiagen)		
[318]	2020	70	animal	swabs and tissue specimens	DNA	illumina	De novo	Papillomavirus	Papillomaviridae,	/		RNeasy Lipid Tissue Mini kit (Qiagen)	Column-based	
[319]	2019	50	animal	cloacal, oral, and blood samples	DNA	illumina	De novo	Gyrovirus	Anelloviridae,	French Guiana	America region	MagMAX Viral RNA Isolation kit	Magnetic beads-based	
[320]	2020	406	animal	fecal samples	RNA	illumina	De novo	Picornaviridae, Reoviridae Hepeviridae.	Picornaviridae, Hepeviridae, Reoviridae,	French Guiana	America region	MagMAX Viral RNA Isolation kit	Magnetic beads-based	
[321]	2012	156	animal	Rectal swabs	DNA	Roche_454	De novo	Papillomavirus	Papillomaviridae,	Hong Kong	South-East Asia region	QIAamp viral RNA mini kit (Qiagen)	Other/multiple type	
[322]	2017	23	animal	fecal samples	RNA	illumina	De novo	Enterovirus	Picornaviridae,	Japan	South-East Asia region	TRIzol reagents	Solvent-based	
[323]	2018	222	animal	fecal samples	RNA	illumina	De novo	bovine enterovirus	Picornaviridae,	Japan	South-East Asia region	TRIzol reagents	Solvent-based	
[324]	2021	1000	animal	fecal samples	RNA	illumina	De novo	Picobirnavirus	Picobirnaviridae,	China	South-East Asia region	QIAamp viral RNA mini kit (Qiagen)	Other/multiple type	
[325]	2018	4	environment	water	RNA	illumina	De novo	Several	Megabirnaviridae, Hypoviridae	North Pacific Ocean		Phenol-chloroform extraction method	Solvent-based	FeCl3 precipitation
[326]	2009	59	animal	crop, poison sac, midgut, hindgut, Malpighian tubules, fat body (queens only), ovaries (queens only), and the remaining tissues of the abdominal carcass.	RNA	sanger	/	Solenopsis invicta virus 3	Solinviviridae,	/		TRIzol reagents	Solvent-based	
[327]	2019	3189	animal	insects	RNA	illumina	De novo	several	Dicistroviridae, Dicistroviridae, Dicistroviridae, Dicistroviridae, Polycipiviridae,	Argentina	America region	TRIzol reagents	Solvent-based	

									Iflaviridae, Totiviridae, Unclassified, Unclassified,				
[328]	2012	609	animal	insects	RNA	Roche_454	De novo	Polydnavirus	Polyomaviridae,	Florida	America region	TRIzol reagents	Solvent-based
[329]	2018	2730	animal	insects	RNA	illumina	De novo	SINV-5	Unclassified,	Argentina	America region	TRIzol reagents	Solvent-based
[330]	2017	151	animal	intestinal content samples	DNA	Roche_454	De novo	Adenovirus	Adenoviridae,	North Sea	America region	Nucleospin RNA XS Kit (Machery-Nagel)	Silica membrane-based
[331]	2020	978	animal	serum	RNA	illumina	De novo	Flavivirus	Flaviviridae,	Pennsylvania	America region	QIAamp viral RNA mini kit (Qiagen)	Other/multiple type
[332]	2021	767	animal	insects	RNA	illumina	/	coltiviruses, reoviruses	Reoviridae, Reoviridae	Belgium	European region	QIAamp viral RNA mini kit (Qiagen)	Other/multiple type
[333]	2020	ND	animal	fecal samples	DNA and RNA	Ion Torrent	/	Aviadenovirus, gyrovirus	Adenoviridae, Anelloviridae	Australia	South-East Asia region	QIAamp viral RNA mini kit (Qiagen)	Other/multiple type
[334]	2020	ND	animal	fecal samples	DNA and RNA	Ion Torrent	/	Parvoviruses, Picornaviruses	Picornaviridae, Parvoviridae	Australia	South-East Asia region	QIAamp viral RNA mini kit (Qiagen)	Other/multiple type
[335]	2008	5	animal	insect pools, skunk brain, human feces and sewer effluent	RNA	Not available	/	Orthoreovirus, orbivirus	Reoviridae,	Montana	America region	Qiamp Viral RNA isolation kit (Qiagen)	Column-based
[336]	2018	5	environment	water	DNA	illumina	De novo	Holosalinivirus	Siphoviridae,	Spain	European region	/	ND
[337]	2021	253	animal	fecal samples	RNA	illumina	/	SqMV	Secoviridae,	Brazil	America region	/	ND
[338]	2016	14	environment	cyanobacterial mat samples	DNA	illumina	De novo	PhV1	Herpesviridae,	Lake Huron	America region	/	ND
[339]	2016	22	animal	blood and fecal samples	DNA	sanger	De novo	FelineAV621	Anelloviridae,	China	South-East Asia region	/	ND

[340]	2017	46	animal	fecal samples, blood, nasopharyngeal secretion, heart, liver, spleen, lung, and kidney tissues	DNA and RNA	illumina	De novo	several	Papillomaviridae, Picornaviridae, Unclassified, Anelloviridae, Genomoviridae,	China	South-East Asia region	QIAamp viral RNA mini kit (Qiagen)	Other/multiple type	
[341]	2021	ND	environment	water	DNA	illumina	De novo	vB_OliS_GJ44	Siphoviridae,	Yellow Sea	South-East Asia region	/	ND	tangential flow filtration
[342]	2014	22	animal	meats	DNA	illumina	De novo	BPyV2-SF, GyV7-SF	Polyomaviridae,	United States	America region	MagMAX Viral RNA Isolation kit	Magnetic beads-based	
[343]	2014	25	animal	fecal samples	DNA and RNA	illumina	De novo	astroviruses bocaviruses, cyclovirus	Astroviridae, Circoviridae, Parvoviridae,	California	America region	MagMAX Viral RNA Isolation kit	Magnetic beads-based	
[344]	2017	18	plant	maize samples	RNA	illumina	De novo	BSRV)-like dicistrovirus	Dicistroviridae,	Kenya	Africa region	MagNA Pure LC - DNA Kit (Roche)	Magnetic glass particles-based	
[345]	2018	520	animal	serum	DNA	illumina	De novo	Parvovirus anellovirus	Parvoviridae, Anelloviridae	Brazil	America region	TRIzol reagents, phenol-chloroform extraction method	Solvent-based, Solvent-based	
[346]	2016	1600	animal	insects	RNA	illumina	De novo	Iflaviruses, Rhabdoviruses, Nodaviruses, Reoviruses, Negeviruses, Sobemoviruses, Poleroviruses, Flaviviridae, Tombusviridae	Iflaviridae, Rhabdoviridae, Nodaviridae, Reoviridae, Unclassified, Solemoviridae, Solemoviridae, Flaviviridae, Tombusviridae,	UK	European region	TRIzol reagents	Solvent-based	
[347]	2020	300	animal	insects	DNA and RNA	illumina	/	Jogalong virus, Parry's Creek negev-like virus 1, Broome virga-like virus 1, Fitzroy Crossing iflavirus 1, Broome luteo-like virus 1, Broome phasivirus 1, Parry's Creek phasivirus 1, Fitzroy Crossing tenui-like virus 1, Fitzroy Crossing qinvirus 1, Broome reo-like virus 1, Fitzroy Crossing toti-	Flaviviridae, Unclassified, Unclassified, Iflaviridae, Unclassified, Phenuiviridae, Phenuiviridae, Phenuiviridae, Unclassified, Reoviridae, Unclassified, Unclassified, Unclassified, Chrysoviridae, Unclassified, Parvoviridae	Australia	South-East Asia region	MagMAX Express-96 Deep Well Magnetic Particle Processor	Magnetic beads-based	

								like virus 1, Fitzroy Crossing toti-like virus 2, Parry's Creek toti-like virus 1, Broome chryso-like virus 1, Broome partiti-like virus 1, Broome densovirus 1						
[348]	2020	13	environment	water	DNA	Roche_454	De novo	Several	Podoviridae, Siphoviridae, Myoviridae,	China	South-East Asia region	DNeasy Blood & Tissue Kit (Qiagen)	Column-based	ultracentrifugation, tangential flow filtration
[349]	2016	16	animal	brain sample	RNA	illumina	De novo	BoRV-CH15.	Retroviridae,	Switzerland	European region	TRIzol reagents	Solvent-based	
[350]	2020	24	animal	bone marrow	DNA	illumina	De novo	Anellovirus	Anelloviridae,	China	South-East Asia region	QIAamp MinElute Virus Spin Kit (Qiagen)	Column-based	
[351]	2015	20	animal	gut	RNA	illumina	De novo	astrovirus	Astroviridae,	China	South-East Asia region	QIAamp viral RNA mini kit (Qiagen)	Other/multiple type	
[352]	2020	10	animal	liver, heart, intestine and stomach	DNA	illumina	/	parvovirus	Parvoviridae,	China	South-East Asia region	QIAamp Viral RNA Mini Elute Kit (Qiagen)	Column-based	
[353]	2020	215	animal	fecal samples	RNA	illumina	De novo	astrovirus	Astroviridae,	China	South-East Asia region	QIAamp viral RNA mini kit (Qiagen)	Other/multiple type	
[354]	2016	69	animal	fecal samples	RNA	illumina	/	Enterovirus	Picornaviridae,	China	South-East Asia region	MiniBEST Viral RNA/DNA Extraction Kit (TaKaRa)	Column-based	
[355]	2018	2222	animal	insects	RNA	illumina	De novo	several	Unclassified, Phenuiviridae, Rhabdoviridae, Unclassified, Picornaviridae, Rhabdoviridae, Unclassified, Unclassified	China	South-East Asia region	QIAamp viral RNA kit (Qiagen)	Silica membrane-based	
[356]	2015	5	environment	ballast water	DNA	illumina	De novo	Cyprinid herpesvirus 3	Alloherpesviridae,	Minnesota	America region	QIAamp MinElute Virus Spin Kit (Qiagen)	Column-based	
[357]	2021	14	animal	fecal samples	DNA and RNA	illumina	De novo	astrovirus, chaphamaparvovirus , nodavirus, densoviruses, CRESS-DNA viruses	Astroviridae, Parvoviridae, Nodaviridae, Parvoviridae, ND,	California	America region	QIAamp virus minikit (Qiagen)	Column-based	

[358]	2021	12	animal	fecal samples	RNA	illumina	De novo	astrovirus	Astroviridae,	Canada	America region	MagMAX Viral RNA Isolation kit	Magnetic beads-based
[359]	2020	43	animal	fecal samples	DNA	illumina	De novo	bocavirus	Parvoviridae,	Canada	America region	QIAamp MinElute Virus Spin Kit (Qiagen)	Column-based
[360]	2020	ND	animal	liver, spleen and brain	RNA	Ion Torrent	/	picornavirus	Picornaviridae,	China	South-East Asia region	/	ND
[361]	2018	40	animal	fecal samples	RNA	illumina	/	Picornavirus, parvovirus	Picornaviridae, Parvoviridae	China	South-East Asia region	/	ND
[362]	2019	93	animal	fecal samples	DNA and RNA	illumina	De novo	Cardiovirus	Picornaviridae,	China	South-East Asia region	QIAamp viral RNA mini kit (Qiagen)	Other/multiple type
[363]	2017	1	animal	fecal samples	RNA	Ion Torrent	/	Parainfluenza virus	Paramyxoviridae,	China	South-East Asia region	QIAamp viral RNA mini kit (Qiagen)	Other/multiple type
[364]	2016	48	animal	infected gingival epithelium samples	DNA	illumina	De novo	TTMV-222	Anelloviridae,	China	South-East Asia region	QIAamp viral RNA kit (Qiagen)	Silica membrane-based
[365]	2017	48	animal	periodontal pockets gingival epithelium and connective tissue facing the sulcus	DNA	illumina	De novo	TTMV-204	Anelloviridae,	China	South-East Asia region	/	ND
[366]	2019	48	animal	periodontal pockets gingival epithelium and connective tissue facing the sulcus	DNA	illumina	De novo	Siphoviridae_29632	Siphoviridae,	China	South-East Asia region	QIAamp viral RNA kit (Qiagen)	Silica membrane-based
[367]	2016	87	animal	fecal samples	RNA	illumina	De novo	Rotaviruses	Sedoreoviridae,	Cameroon	Africa region	QIAamp viral RNA mini kit (Qiagen)	Other/multiple type
[368]	2018	87	animal	fecal samples	DNA and RNA	illumina	De novo	Picorna virus	Picornaviridae,	Cameroon	Africa region	/	ND

[369]	2017	87	animal	fecal samples	RNA	illumina	De novo	sapovirus	Caliciviridae,	Cameroon	Africa region	QIAamp viral RNA mini kit (Qiagen)	Other/multiple type	
[370]	2017	87	animal	fecal samples	RNA	illumina	De novo	Sapovirus	Caliciviridae,	Cameroon	Africa region	/	ND	
[371]	2015	1	animal	gut	DNA	Analyzes sequences obtained in other studies	/	bacteriophage	Mimiviridae,	Wyoming	America region	/	ND	
[372]	2021	300	animal	insects	DNA	illumina	De novo	Hepe-like virus	Hepeviridae,	China	South-East Asia region	QIAamp viral RNA mini kit (Qiagen)	Other/multiple type	
[373]	2021	200	animal	nasopharyngeal swabs	RNA	illumina	De novo	Coltivirus	Reoviridae,	Saudi Arabia	Eastern Mediterranean region	/	ND	
[374]	2020	Database	environment	water	DNA	Analyzes sequences obtained in other studies	/	SAR11 phage	Myoviridae,	/		/	ND	
[375]	2016	1	environment	water	DNA	illumina	De novo	DSS3Φ8	Siphoviridae,	Meryland	America region	Phenol-chloroform extraction method	Solvent-based	pore filtration
[376]	2015	9	animal	fecal samples	RNA	sanger	/	Sicinivirus	Picornaviridae,	China	South-East Asia region	QIAamp MinElute Virus Spin Kit (Qiagen)	Column-based	
[377]	2018	30	animal	fecal samples	DNA	illumina	De novo	TM7x	ND,	United States	America region	Chemagic MSM I (Perkin Elmer)	Magnetic beads-based	
[378]	2011	42	animal	enteric samples	DNA	sanger	De novo	Microviridae (batteriofago)	Microviridae,	California	America region	/	ND	
[379]	2015	ND	Not available	Intestinal bacteriophage	DNA	illumina	De novo	Proteus phage cluster (Siphoviridae)	Siphoviridae,	Georgia	European region	Phage DNA isolation kit (NorgenBiotek)	Column-based	

1. Abi, K.M., et al., *Identification of a novel Aichivirus D in sheep.* Infect Genet Evol, 2021. **91**: p. 104810.
2. Aguirre de Cárcer, D., et al., *Biodiversity and distribution of polar freshwater DNA viruses.* Sci Adv, 2015. **1**(5): p. e1400127.
3. Al Rwahnih, M., et al., *Prunus geminivirus A: A Novel Grablovirus Infecting Prunus spp.* Plant Dis, 2018. **102**(7): p. 1246-1253.
4. Alarcón-Schumacher, T., et al., *Elucidating Viral Communities During a Phytoplankton Bloom on the West Antarctic Peninsula.* Front Microbiol, 2019. **10**: p. 1014.
5. Alarcón-Schumacher, T., et al., *Ecogenomics and Adaptation Strategies of Southern Ocean Viral Communities.* mSystems, 2021. **6**(4): p. e0039621.
6. Albariño, C.G., et al., *Novel paramyxovirus associated with severe acute febrile disease, South Sudan and Uganda, 2012.* Emerg Infect Dis, 2014. **20**(2): p. 211-6.
7. Alex, C.E., et al., *Amdoparvovirus Infection in Red Pandas (Ailurus fulgens).* Vet Pathol, 2018. **55**(4): p. 552-561.
8. Alex, C.E., et al., *Viruses in unexplained encephalitis cases in American black bears (Ursus americanus).* PLoS One, 2020. **15**(12): p. e0244056.
9. Altan, E., et al., *Complex Virome in a Mesenteric Lymph Node from a Californian Sea Lion (Zalophus Californianus) with Polyserositis and Steatitis.* Viruses, 2020. **12**(8).
10. Altan, E., et al., *New Parvoviruses and Picornavirus in Tissues and Feces of Foals with Interstitial Pneumonia.* Viruses, 2021. **13**(8).
11. Altan, E., et al., *A Highly Divergent Picornavirus Infecting the Gut Epithelia of Zebrafish (Danio rerio) in Research Institutions Worldwide.* Zebrafish, 2019. **16**(3): p. 291-299.
12. Altan, E., et al., *Viruses in Horses with Neurologic and Respiratory Diseases.* Viruses, 2019. **11**(10).
13. Altan, E., et al., *Nasal virome of dogs with respiratory infection signs include novel taupapillomaviruses.* Virus Genes, 2019. **55**(2): p. 191-197.
14. Amgarten, D., et al., *Three novel Pseudomonas phages isolated from composting provide insights into the evolution and diversity of tailed phages.* BMC Genomics, 2017. **18**(1): p. 346.
15. Andreani, J., et al., *Morphological and Genomic Features of the New Klosneuvirinae Isolate Fadolivirus IHUMI-VV54.* Front Microbiol, 2021. **12**: p. 719703.
16. Anh, N.T., et al., *Viruses in Vietnamese Patients Presenting with Community-Acquired Sepsis of Unknown Cause.* J Clin Microbiol, 2019. **57**(9).
17. Arroyo Mühr, L.S., et al., *Does human papillomavirus-negative condylomata exist?* Virology, 2015. **485**: p. 283-8.
18. Aswad, A. and A. Katzourakis, *The first endogenous herpesvirus, identified in the tarsier genome, and novel sequences from primate rhadinoviruses and lymphocryptoviruses.* PLoS Genet, 2014. **10**(6): p. e1004332.
19. Aswad, A. and A. Katzourakis, *A novel viral lineage distantly related to herpesviruses discovered within fish genome sequence data.* Virus Evol, 2017. **3**(2): p. vex016.
20. Aw, T.G., A. Howe, and J.B. Rose, *Metagenomic approaches for direct and cell culture evaluation of the virological quality of wastewater.* J Virol Methods, 2014. **210**: p. 15-21.
21. Zhang, B., et al., *Viral metagenomics analysis demonstrates the diversity of viral flora in piglet diarrhoeic faeces in China.* J Gen Virol, 2014. **95**(Pt 7): p. 1603-1611.
22. Bäckström, D., et al., *Virus Genomes from Deep Sea Sediments Expand the Ocean Megavirome and Support Independent Origins of Viral Gigantism.* mBio, 2019. **10**(2).
23. Bag, S., et al., *Detection of a New Luteovirus in Imported Nectarine Trees: A Case Study to Propose Adoption of Metagenomics in Post-Entry Quarantine.* Phytopathology, 2015. **105**(6): p. 840-6.
24. Baidaliuk, A., et al., *Novel genome sequences of cell-fusing agent virus allow comparison of virus phylogeny with the genetic structure of Aedes aegypti populations.* Virus Evol, 2020. **6**(1): p. veaa018.
25. Bányai, K., et al., *Candidate new rotavirus species in Schreiber's bats, Serbia.* Infect Genet Evol, 2017. **48**: p. 19-26.
26. Barrientos-Somarribas, M., et al., *Discovering viral genomes in human metagenomic data by predicting unknown protein families.* Sci Rep, 2018. **8**(1): p. 28.
27. Beaulaurier, J., et al., *Assembly-free single-molecule sequencing recovers complete virus genomes from natural microbial communities.* Genome Res, 2020. **30**(3): p. 437-446.
28. Benites, L.F., et al., *Single cell ecogenomics reveals mating types of individual cells and ssDNA viral infections in the smallest photosynthetic eukaryotes.* Philos Trans R Soc Lond B Biol Sci, 2019. **374**(1786): p. 20190089.

29. Bertazzon, N., et al., *Two New Putative Plant Viruses from Wood Metagenomics Analysis of an Esca Diseased Vineyard.* Plants (Basel), 2020. **9**(7).
30. Birnberg, L., et al., *Viromics on Honey-Baited FTA Cards as a New Tool for the Detection of Circulating Viruses in Mosquitoes.* Viruses, 2020. **12**(3).
31. Bischoff, V., et al., *Cobaviruses - a new globally distributed phage group infecting Rhodobacteraceae in marine ecosystems.* Isme j, 2019. **13**(6): p. 1404-1421.
32. Blomström, A.L., et al., *Detection of a novel astrovirus in brain tissue of mink suffering from shaking mink syndrome by use of viral metagenomics.* J Clin Microbiol, 2010. **48**(12): p. 4392-6.
33. Blomström, A.L., et al., *Viral metagenomic analysis of bushpigs (Potamochoerus larvatus) in Uganda identifies novel variants of Porcine parvovirus 4 and Torque teno sus virus 1 and 2.* Virol J, 2012. **9**: p. 192.
34. Blomström, A.L., et al., *Viral Metagenomic Analysis Displays the Co-Infection Situation in Healthy and PMWS Affected Pigs.* PLoS One, 2016. **11**(12): p. e0166863.
35. Blomström, A.L., et al., *Novel Viruses Found in Antricola Ticks Collected in Bat Caves in the Western Amazonia of Brazil.* Viruses, 2019. **12**(1).
36. Blouin, A.G., et al., *A new virus discovered by immunocapture of double-stranded RNA, a rapid method for virus enrichment in metagenomic studies.* Mol Ecol Resour, 2016. **16**(5): p. 1255-63.
37. Bodewes, R., et al., *Identification of DNA sequences that imply a novel gammaherpesvirus in seals.* J Gen Virol, 2015. **96**(Pt 5): p. 1109-1114.
38. Bolatti, E.M., et al., *A Preliminary Study of the Virome of the South American Free-Tailed Bats (Tadarida brasiliensis) and Identification of Two Novel Mammalian Viruses.* Viruses, 2020. **12**(4).
39. Bolduc, B., et al., *Identification of novel positive-strand RNA viruses by metagenomic analysis of archaea-dominated Yellowstone hot springs.* J Virol, 2012. **86**(10): p. 5562-73.
40. Bolduc, B., et al., *Viral assemblage composition in Yellowstone acidic hot springs assessed by network analysis.* Isme j, 2015. **9**(10): p. 2162-77.
41. Boratto, P.V.M., et al., *Yaravirus: A novel 80-nm virus infecting Acanthamoeba castellanii.* Proc Natl Acad Sci U S A, 2020. **117**(28): p. 16579-16586.
42. Boros, Á., et al., *High prevalence, genetic diversity and a potentially novel genotype of Sapelovirus A (Picornaviridae) in enteric and respiratory samples in Hungarian swine farms.* J Gen Virol, 2020. **101**(6): p. 609-621.
43. Boukari, W., et al., *Occurrence of a novel mastrevirus in sugarcane germplasm collections in Florida, Guadeloupe and Réunion.* Virol J, 2017. **14**(1): p. 146.
44. Bouzalas, I.G., et al., *Neurotropic astrovirus in cattle with nonsuppurative encephalitis in Europe.* J Clin Microbiol, 2014. **52**(9): p. 3318-24.
45. Buck, C.B., et al., *The Ancient Evolutionary History of Polyomaviruses.* PLoS Pathog, 2016. **12**(4): p. e1005574.
46. Campbell, S.J., et al., *Red fox viromes in urban and rural landscapes.* Virus Evol, 2020. **6**(2): p. veaa065.
47. Candresse, T., A. Marais, and C. Faure, *First Report of Southern tomato virus on Tomatoes in Southwest France.* Plant Dis, 2013. **97**(8): p. 1124.
48. Canova, R., et al., *Spleen and lung virome analysis of South American fur seals (Arctocephalus australis) collected on the southern Brazilian coast.* Infect Genet Evol, 2021. **92**: p. 104862.
49. Cardeti, G., et al., *Fatal Outbreak in Tonkean Macaques Caused by Possibly Novel Orthopoxvirus, Italy, January 2015 (1).* Emerg Infect Dis, 2017. **23**(12): p. 1941-1949.
50. Carradec, Q., et al., *A global ocean atlas of eukaryotic genes.* Nat Commun, 2018. **9**(1): p. 373.
51. Carrai, M., et al., *Identification of A Novel Papillomavirus Associated with Squamous Cell Carcinoma in A Domestic Cat.* Viruses, 2020. **12**(1).
52. Carvalho Costa, L., et al., *Identification and characterization of a novel virus associated with an eriophyid mite in extracts of fruit trees leaves.* Arch Virol, 2021. **166**(10): p. 2869-2873.
53. Castañeda-Mogollón, D., et al., *A metagenomics workflow for SARS-CoV-2 identification, co-pathogen detection, and overall diversity.* J Clin Virol, 2021. **145**: p. 105025.
54. Castelán-Sánchez, H.G., et al., *Intermediate-Salinity Systems at High Altitudes in the Peruvian Andes Unveil a High Diversity and Abundance of Bacteria and Viruses.* Genes (Basel), 2019. **10**(11).

55. Castrignano, S.B., et al., *Two novel circo-like viruses detected in human feces: complete genome sequencing and electron microscopy analysis.* Virus Res, 2013. **178**(2): p. 364-73.
56. Castrignano, S.B., et al., *Identification of circo-like virus-Brazil genomic sequences in raw sewage from the metropolitan area of São Paulo: evidence of circulation two and three years after the first detection.* Mem Inst Oswaldo Cruz, 2017. **112**(3): p. 175-181.
57. Cebriá-Mendoza, M., et al., *Deep viral blood metagenomics reveals extensive anellovirus diversity in healthy humans.* Sci Rep, 2021. **11**(1): p. 6921.
58. Chandler, J.A., R.M. Liu, and S.N. Bennett, *RNA shotgun metagenomic sequencing of northern California (USA) mosquitoes uncovers viruses, bacteria, and fungi.* Front Microbiol, 2015. **6**: p. 185.
59. Charles, J., et al., *Detection of novel and recognized RNA viruses in mosquitoes from the Yucatan Peninsula of Mexico using metagenomics and characterization of their in vitro host ranges.* J Gen Virol, 2018. **99**(12): p. 1729-1738.
60. Cheung, A.K., et al., *A divergent clade of circular single-stranded DNA viruses from pig feces.* Arch Virol, 2013. **158**(10): p. 2157-62.
61. Cholleti, H., et al., *Discovery of Novel Viruses in Mosquitoes from the Zambezi Valley of Mozambique.* PLoS One, 2016. **11**(9): p. e0162751.
62. Chong, R., et al., *Fecal Viral Diversity of Captive and Wild Tasmanian Devils Characterized Using Virion-Enriched Metagenomics and Metatranscriptomics.* J Virol, 2019. **93**(11).
63. Cibulski, S., et al., *Viral metagenomics in Brazilian Pekin ducks identifies two gyrovirus, including a new species, and the potentially pathogenic duck circovirus.* Virology, 2020. **548**: p. 101-108.
64. Cibulski, S., et al., *A plate of viruses: Viral metagenomics of supermarket chicken, pork and beef from Brazil.* Virology, 2021. **552**: p. 1-9.
65. Claverie, S., et al., *Exploring the diversity of Poaceae-infecting mastreviruses on Reunion Island using a viral metagenomics-based approach.* Sci Rep, 2019. **9**(1): p. 12716.
66. Coffey, L.L., et al., *Enhanced arbovirus surveillance with deep sequencing: Identification of novel rhabdoviruses and bunyaviruses in Australian mosquitoes.* Virology, 2014. **448**: p. 146-58.
67. Conceição-Neto, N., et al., *Viral gut metagenomics of sympatric wild and domestic canids, and monitoring of viruses: Insights from an endangered wolf population.* Ecol Evol, 2017. **7**(12): p. 4135-4146.
68. Correa, A.M., et al., *Viral Outbreak in Corals Associated with an In Situ Bleaching Event: Atypical Herpes-Like Viruses and a New Megavirus Infecting Symbiodinium.* Front Microbiol, 2016. **7**: p. 127.
69. Coutinho, F.H., R.A. Edwards, and F. Rodríguez-Valera, *Charting the diversity of uncultured viruses of Archaea and Bacteria.* BMC Biol, 2019. **17**(1): p. 109.
70. Crane, A., et al., *Novel anelloviruses identified in buccal swabs of Antarctic fur seals.* Virus Genes, 2018. **54**(5): p. 719-723.
71. Wang, D., et al., *First report of Sida leaf curl virus and associated betasatellite in tobacco.* Plant Dis, 2021.
72. Zhang, D., et al., *Isolation and Characterization of the First Freshwater Cyanophage Infecting Pseudanabaena.* J Virol, 2020. **94**(17).
73. da Silva Neves, N.A., et al., *Sialovirome of Brazilian tropical anophelines.* Virus Res, 2021. **302**: p. 198494.
74. Dacheux, L., et al., *A preliminary study of viral metagenomics of French bat species in contact with humans: identification of new mammalian viruses.* PLoS One, 2014. **9**(1): p. e87194.
75. Dai, Z., et al., *Identification of a novel circovirus in blood sample of giant pandas (Ailuropoda melanoleuca).* Infect Genet Evol, 2021. **95**: p. 105077.
76. Dai, Z., et al., *Identification and characterization of a novel bocaparvovirus in tufted deer (Elaphodus cephalophus) in China.* Arch Virol, 2021: p. 1-6.
77. Daugrois, J.H., et al., *Comparison of the Virome of Quarantined Sugarcane Varieties and the Virome of Grasses Growing near the Quarantine Station.* Viruses, 2021. **13**(5).
78. Dayaram, A., et al., *Molecular characterisation of a novel cassava associated circular ssDNA virus.* Virus Res, 2012. **166**(1-2): p. 130-5.
79. Dayaram, A., et al., *Novel circular DNA viruses identified in Procordulia grayi and Xanthocnemis zealandica larvae using metagenomic approaches.* Infect Genet Evol, 2014. **22**: p. 134-41.

80. de Nazaré Almeida Dos Reis, L., et al., *Metagenomics of Neotropical Single-Stranded DNA Viruses in Tomato Cultivars with and without the Ty-1 Gene.* Viruses, 2020. **12**(8).
81. de Souza, W.M., et al., *Pingu virus: A new picornavirus in penguins from Antarctica.* Virus Evol, 2019. **5**(2): p. vez047.
82. Deaton, J., F.B. Yu, and S.R. Quake, *Mini-Metagenomics and Nucleotide Composition Aid the Identification and Host Association of Novel Bacteriophage Sequences.* Adv Biosyst, 2019. **3**(11): p. e1900108.
83. Deboutte, W., et al., *Honey-bee-associated prokaryotic viral communities reveal wide viral diversity and a profound metabolic coding potential.* Proc Natl Acad Sci U S A, 2020. **117**(19): p. 10511-10519.
84. Dela Cruz, F.N., Jr., et al., *A novel pulmonary polyomavirus in alpacas (Vicugna pacos).* Vet Microbiol, 2017. **201**: p. 49-55.
85. Deng, L., et al., *Viral tagging reveals discrete populations in Synechococcus viral genome sequence space.* Nature, 2014. **513**(7517): p. 242-5.
86. Dennis, T.P.W., et al., *Insights into Circovirus Host Range from the Genomic Fossil Record.* J Virol, 2018. **92**(16).
87. Devaney, R., et al., *A metagenomic comparison of endemic viruses from broiler chickens with runting-stunting syndrome and from normal birds.* Avian Pathol, 2016. **45**(6): p. 616-629.
88. Diemer, G.S. and K.M. Stedman, *A novel virus genome discovered in an extreme environment suggests recombination between unrelated groups of RNA and DNA viruses.* Biol Direct, 2012. **7**: p. 13.
89. Dill, J.A., et al., *Microscopic and Molecular Evidence of the First Elasmobranch Adomavirus, the Cause of Skin Disease in a Giant Guitarfish, Rhynchobatus djiddensis.* mBio, 2018. **9**(3).
90. Divers, T.J., et al., *New Parvovirus Associated with Serum Hepatitis in Horses after Inoculation of Common Biological Product.* Emerg Infect Dis, 2018. **24**(2): p. 303-310.
91. Ducatez, M.F. and J.L. Guérin, *Identification of a novel coronavirus from guinea fowl using metagenomics.* Methods Mol Biol, 2015. **1282**: p. 27-31.
92. Dumarest, M., et al., *Viral diversity in swine intestinal mucus used for the manufacture of heparin as analyzed by high-throughput sequencing.* Biologicals, 2015. **43**(1): p. 31-6.
93. Dunlap, D.S., et al., *Molecular and microscopic evidence of viruses in marine copepods.* Proc Natl Acad Sci U S A, 2013. **110**(4): p. 1375-80.
94. Duraisamy, R., et al., *Detection of novel RNA viruses from free-living gorillas, Republic of the Congo: genetic diversity of picobirnaviruses.* Virus Genes, 2018. **54**(2): p. 256-271.
95. Dutilh, B.E., et al., *A highly abundant bacteriophage discovered in the unknown sequences of human faecal metagenomes.* Nat Commun, 2014. **5**: p. 4498.
96. Edridge, A.W.D., et al., *Novel Orthobunyavirus Identified in the Cerebrospinal Fluid of a Ugandan Child With Severe Encephalopathy.* Clin Infect Dis, 2019. **68**(1): p. 139-142.
97. Eibach, D., et al., *Viral metagenomics revealed novel betatorquevirus species in pediatric inpatients with encephalitis/meningoencephalitis from Ghana.* Sci Rep, 2019. **9**(1): p. 2360.
98. Emmerich, P., et al., *Viral metagenomics, genetic and evolutionary characteristics of Crimean-Congo hemorrhagic fever orthonairovirus in humans, Kosovo.* Infect Genet Evol, 2018. **65**: p. 6-11.
99. Yang, F., et al., *[Metagenomic analysis of bat virome in several Chinese regions].* Sheng Wu Gong Cheng Xue Bao, 2013. **29**(5): p. 586-600.
100. Fahsbender, E., et al., *Chapparvovirus DNA Found in 4% of Dogs with Diarrhea.* Viruses, 2019. **11**(5).
101. Fahsbender, E., et al., *Plasma virome of 781 Brazilians with unexplained symptoms of arbovirus infection include a novel parvovirus and densovirus.* PLoS One, 2020. **15**(3): p. e0229993.
102. Faizah, A.N., et al., *Deciphering the Virome of Culex vishnui Subgroup Mosquitoes, the Major Vectors of Japanese Encephalitis, in Japan.* Viruses, 2020. **12**(3).
103. Fauver, J.R., et al., *West African Anopheles gambiae mosquitoes harbor a taxonomically diverse virome including new insect-specific flaviviruses, mononegaviruses, and totiviruses.* Virology, 2016. **498**: p. 288-299.

104. Fehér, E., et al., *Genomic Diversity of CRESS DNA Viruses in the Eukaryotic Virome of Swine Feces.* Microorganisms, 2021. **9**(7).
105. Feng, C., et al., *Identification of the Viral Determinant of Hypovirulence and Host Range in Sclerotiniaceae of a Genomovirus Reconstructed from the Plant Metagenome.* J Virol, 2021. **95**(17): p. e0026421.
106. Fernandez-Cassi, X., et al., *A metagenomic assessment of viral contamination on fresh parsley plants irrigated with fecally tainted river water.* Int J Food Microbiol, 2017. **257**: p. 80-90.
107. Fernández-Correa, I., et al., *A novel group of avian astroviruses from Neotropical passerine birds broaden the diversity and host range of Astroviridae.* Sci Rep, 2019. **9**(1): p. 9513.
108. Filipa-Silva, A., et al., *The Unexplored Virome of Two Atlantic Coast Fish: Contribution of Next-Generation Sequencing to Fish Virology.* Foods, 2020. **9**(11).
109. Filloux, D., et al., *Metagenomics Approaches Based on Virion-Associated Nucleic Acids (VANA): An Innovative Tool for Assessing Without A Priori Viral Diversity of Plants.* Methods Mol Biol, 2015. **1302**: p. 249-57.
110. Filloux, D., et al., *Viral Metagenomic-Based Screening of Sugarcane from Florida Reveals Occurrence of Six Sugarcane-Infecting Viruses and High Prevalence of Sugarcane yellow leaf virus.* Plant Dis, 2018. **102**(11): p. 2317-2323.
111. Fitzgerald, C.B., et al., *Probing the "Dark Matter" of the Human Gut Phageome: Culture Assisted Metagenomics Enables Rapid Discovery and Host-Linking for Novel Bacteriophages.* Front Cell Infect Microbiol, 2021. **11**: p. 616918.
112. Fontenele, R.S., et al., *Discovery of the first maize-infecting mastrevirus in the Americas using a vector-enabled metagenomics approach.* Arch Virol, 2018. **163**(1): p. 263-267.
113. Fontenele, R.S., et al., *Single Stranded DNA Viruses Associated with Capybara Faeces Sampled in Brazil.* Viruses, 2019. **11**(8).
114. Font-Verdera, F., et al., *Inverted microbial community stratification and spatial-temporal stability in hypersaline anaerobic sediments from the S'Avall solar salterns.* Syst Appl Microbiol, 2021. **44**(5): p. 126231.
115. François, S., et al., *A New Prevalent Densovirus Discovered in Acari. Insight from Metagenomics in Viral Communities Associated with Two-Spotted Mite (Tetranychus urticae) Populations.* Viruses, 2019. **11**(3).
116. François, S., et al., *Characterisation of the Viral Community Associated with the Alfalfa Weevil (Hypera postica) and Its Host Plant, Alfalfa (Medicago sativa).* Viruses, 2021. **13**(5).
117. Frey, K.G., et al., *Bioinformatic Characterization of Mosquito Viromes within the Eastern United States and Puerto Rico: Discovery of Novel Viruses.* Evol Bioinform Online, 2016. **12**(Suppl 2): p. 1-12.
118. Wang, G., et al., *Identification and genome analysis of a novel picornavirus from captive belugas (Delphinapterus leucas) in China.* Sci Rep, 2021. **11**(1): p. 21018.
119. Zhao, G., et al., *VirusSeeker, a computational pipeline for virus discovery and virome composition analysis.* Virology, 2017. **503**: p. 21-30.
120. Galbraith, D.A., et al., *Investigating the viral ecology of global bee communities with high-throughput metagenomics.* Sci Rep, 2018. **8**(1): p. 8879.
121. Gallet, R., et al., *Nanovirus-alphasatellite complex identified in Vicia cracca in the Rhône delta region of France.* Arch Virol, 2018. **163**(3): p. 695-700.
122. Ge, X., et al., *Viral metagenomics analysis of planktonic viruses in East Lake, Wuhan, China.* Virol Sin, 2013. **28**(5): p. 280-90.
123. Geoghegan, J.L., et al., *Virome composition in marine fish revealed by meta-transcriptomics.* Virus Evol, 2021. **7**(1): p. veab005.
124. Gong, C., et al., *Novel Virophages Discovered in a Freshwater Lake in China.* Front Microbiol, 2016. **7**: p. 5.
125. Gonzales-Gustavson, E., et al., *Identification of sapovirus GV.2, astrovirus VA3 and novel anelloviruses in serum from patients with acute hepatitis of unknown aetiology.* PLoS One, 2017. **12**(10): p. e0185911.
126. Guajardo-Leiva, S., et al., *Active Crossfire Between Cyanobacteria and Cyanophages in Phototrophic Mat Communities Within Hot Springs.* Front Microbiol, 2018. **9**: p. 2039.
127. Guajardo-Leiva, S., et al., *Metagenomic Insights into the Sewage RNA Virosphere of a Large City.* Viruses, 2020. **12**(9).
128. Gulino, K., et al., *Initial Mapping of the New York City Wastewater Virome.* mSystems, 2020. **5**(3).

129. Guo, L., et al., *Viral metagenomics analysis of feces from coronary heart disease patients reveals the genetic diversity of the Microviridae.* Virol Sin, 2017. **32**(2): p. 130-138.
130. Li, H., et al., *A novel anellovirus from hospitalized neonates.* Arch Virol, 2021. **166**(9): p. 2623-2625.
131. Kim, H.R., et al., *Identification of a picornavirus from chickens with transmissible viral proventriculitis using metagenomic analysis.* Arch Virol, 2015. **160**(3): p. 701-9.
132. Wang, H., et al., *Gut virome of mammals and birds reveals high genetic diversity of the family Microviridae.* Virus Evol, 2019. **5**(1): p. vez013.
133. Wang, H., et al., *Variations among Viruses in Influent Water and Effluent Water at a Wastewater Plant over One Year as Assessed by Quantitative PCR and Metagenomics.* Appl Environ Microbiol, 2020. **86**(24).
134. Halary, S., et al., *Novel Single-Stranded DNA Circular Viruses in Pericardial Fluid of Patient with Recurrent Pericarditis.* Emerg Infect Dis, 2016. **22**(10): p. 1839-41.
135. Hall, R.J., et al., *New alphacoronavirus in Mystacina tuberculata bats, New Zealand.* Emerg Infect Dis, 2014. **20**(4): p. 697-700.
136. Han, Z., et al., *The Husavirus Posa-Like Viruses in China, and a New Group of Picornavirales.* Viruses, 2020. **12**(9).
137. Hansen, T.A., et al., *New Type of Papillomavirus and Novel Circular Single Stranded DNA Virus Discovered in Urban Rattus norvegicus Using Circular DNA Enrichment and Metagenomics.* PLoS One, 2015. **10**(11): p. e0141952.
138. Hargitai, R., et al., *Detection and genetic characterization of a novel parvovirus distantly related to human bufavirus in domestic pigs.* Arch Virol, 2016. **161**(4): p. 1033-7.
139. Hargitai, R., et al., *Detection and genetic characterization of a novel parvovirus (family Parvoviridae) in barn owls (Tyto alba) in Hungary.* Arch Virol, 2021. **166**(1): p. 231-236.
140. Hargitai, R., et al., *Novel picornavirus (family Picornaviridae) from freshwater fishes (Perca fluviatilis, Sander lucioperca, and Ameiurus melas) in Hungary.* Arch Virol, 2021. **166**(9): p. 2627-2632.
141. Harvey, E., et al., *Extensive Diversity of RNA Viruses in Australian Ticks.* J Virol, 2019. **93**(3).
142. Hayer, J., et al., *Four novel picornaviruses detected in Magellanic Penguins (Spheniscus magellanicus) in Chile.* Virology, 2021. **560**: p. 116-123.
143. He, B., et al., *Virome profiling of bats from Myanmar by metagenomic analysis of tissue samples reveals more novel Mammalian viruses.* PLoS One, 2013. **8**(4): p. e61950.
144. He, B., et al., *Characterization of a novel G3P[3] rotavirus isolated from a lesser horseshoe bat: a distant relative of feline/canine rotaviruses.* J Virol, 2013. **87**(22): p. 12357-66.
145. Hily, J.M., et al., *A genome-wide diversity study of grapevine rupestris stem pitting-associated virus.* Arch Virol, 2018. **163**(11): p. 3105-3111.
146. Hochstein, R.A., et al., *Acidianus Tailed Spindle Virus: a New Archaeal Large Tailed Spindle Virus Discovered by Culture-Independent Methods.* J Virol, 2016. **90**(7): p. 3458-68.
147. Holtz, L.R., et al., *Geographic variation in the eukaryotic virome of human diarrhea.* Virology, 2014. **468-470**: p. 556-564.
148. Houldcroft, C.J., et al., *Identification of novel adenovirus genotype 90 in children from Bangladesh.* Microb Genom, 2018. **4**(10).
149. Hui, A., et al., *Circovirus in Blood of a Febrile Horse with Hepatitis.* Viruses, 2021. **13**(5).
150. Wang, J., et al., *Identification of a novel bocaparvovirus in a wild squirrel in Kunming, Yunnan Province, China.* Arch Virol, 2020. **165**(6): p. 1469-1474.
151. Yang, J., et al., *Viral metagenomic identification of a novel anellovirus in blood sample of a child with atopic dermatitis.* J Med Virol, 2021. **93**(6): p. 4038-4041.
152. Jackson, E.W., et al., *Novel Circular Single-Stranded DNA Viruses among an Asteroid, Echinoid and Holothurian (Phylum: Echinodermata).* PLoS One, 2016. **11**(11): p. e0166093.
153. Jackson, E.W., et al., *A Highly Prevalent and Pervasive Densovirus Discovered among Sea Stars from the North American Atlantic Coast.* Appl Environ Microbiol, 2020. **86**(6).
154. Jin, M., et al., *Diversities and potential biogeochemical impacts of mangrove soil viruses.* Microbiome, 2019. **7**(1): p. 58.
155. Jo, Y., et al., *Comparative Microbiome Study of Mummified Peach Fruits by Metagenomics and Metatranscriptomics.* Plants (Basel), 2020. **9**(8).

156. Kallies, R., et al., *Evaluation of Sequencing Library Preparation Protocols for Viral Metagenomic Analysis from Pristine Aquifer Groundwaters.* Viruses, 2019. **11**(6).
157. Kapusinszky, B., et al., *Local Virus Extinctions following a Host Population Bottleneck.* J Virol, 2015. **89**(16): p. 8152-61.
158. Katsuta, R., et al., *First identification of Sapoviruses in wild boar.* Virus Res, 2019. **271**: p. 197680.
159. Kauer, R.V., et al., *Discovery of novel astrovirus genotype species in small ruminants.* PeerJ, 2019. **7**: p. e7338.
160. Kemenesi, G., et al., *Genetic diversity and recombination within bufaviruses: Detection of a novel strain in Hungarian bats.* Infect Genet Evol, 2015. **33**: p. 288-92.
161. Kinoti, W.M., et al., *Generic Amplicon Deep Sequencing to Determine Ilarvirus Species Diversity in Australian Prunus.* Front Microbiol, 2017. **8**: p. 1219.
162. Kocher, J.F., et al., *Bat Caliciviruses and Human Noroviruses Are Antigenically Similar and Have Overlapping Histo-Blood Group Antigen Binding Profiles.* mBio, 2018. **9**(3).
163. Kothari, A., et al., *Ecogenomics of Groundwater Phages Suggests Niche Differentiation Linked to Specific Environmental Tolerance.* mSystems, 2021. **6**(3): p. e0053721.
164. Kowarsky, M., et al., *Numerous uncharacterized and highly divergent microbes which colonize humans are revealed by circulating cell-free DNA.* Proc Natl Acad Sci U S A, 2017. **114**(36): p. 9623-9628.
165. Kraberger, S., et al., *Diverse single-stranded DNA viruses associated with honey bees (Apis mellifera).* Infect Genet Evol, 2019. **71**: p. 179-188.
166. Kraberger, S., et al., *Unravelling the Single-Stranded DNA Virome of the New Zealand Blackfly.* Viruses, 2019. **11**(6).
167. Kumar, D., et al., *Metagenomic Next-Generation Sequencing Reveal Presence of a Novel Ungulate Bocaparvovirus in Alpacas.* Viruses, 2019. **11**(8).
168. Li, L., et al., *Bat guano virome: predominance of dietary viruses from insects and plants plus novel mammalian viruses.* J Virol, 2010. **84**(14): p. 6955-65.
169. Li, L., et al., *Divergent astrovirus associated with neurologic disease in cattle.* Emerg Infect Dis, 2013. **19**(9): p. 1385-92.
170. Li, L., et al., *Exploring the virome of diseased horses.* J Gen Virol, 2015. **96**(9): p. 2721-2733.
171. Li, L., et al., *Viruses in diarrhoeic dogs include novel kobuviruses and sapoviruses.* J Gen Virol, 2011. **92**(Pt 11): p. 2534-2541.
172. Li, L., et al., *Novel amdovirus in gray foxes.* Emerg Infect Dis, 2011. **17**(10): p. 1876-8.
173. Xu, L., et al., *Characterization of the nasopharyngeal viral microbiome from children with community-acquired pneumonia but negative for Luminex xTAG respiratory viral panel assay detection.* J Med Virol, 2017. **89**(12): p. 2098-2107.
174. Langat, S.K., et al., *Profiling of RNA Viruses in Biting Midges (Ceratopogonidae) and Related Diptera from Kenya Using Metagenomics and Metabarcoding Analysis.* mSphere, 2021. **6**(5): p. e0055121.
175. Law, J., et al., *Identification of hepatotropic viruses from plasma using deep sequencing: a next generation diagnostic tool.* PLoS One, 2013. **8**(4): p. e60595.
176. Lazov, C.M., et al., *Full-Genome Sequences of Alphacoronaviruses and Astroviruses from Myotis and Pipistrelle Bats in Denmark.* Viruses, 2021. **13**(6).
177. Leigh, B.A., et al., *Finer-Scale Phylosymbiosis: Insights from Insect Viromes.* mSystems, 2018. **3**(6).
178. Levin, S., et al., *New Viruses from the Ectoparasite Mite Varroa destructor Infesting Apis mellifera and Apis cerana.* Viruses, 2019. **11**(2).
179. Liais, E., et al., *Novel avian coronavirus and fulminating disease in guinea fowl, France.* Emerg Infect Dis, 2014. **20**(1): p. 105-8.
180. Lima, D.A., et al., *The intestinal virome of malabsorption syndrome-affected and unaffected broilers through shotgun metagenomics.* Virus Res, 2019. **261**: p. 9-20.
181. Ling, Y., et al., *Viral metagenomics reveals significant viruses in the genital tract of apparently healthy dairy cows.* Arch Virol, 2019. **164**(4): p. 1059-1067.
182. Liu, Z., et al., *Identification of a novel human papillomavirus by metagenomic analysis of vaginal swab samples from pregnant women.* Virol J, 2016. **13**: p. 122.
183. M, A.D., et al., *Faecal Virome Analysis of Wild Animals from Brazil.* Viruses, 2019. **11**(9).
184. Ma, Y., et al., *A human gut phage catalog correlates the gut phageome with type 2 diabetes.* Microbiome, 2018. **6**(1): p. 24.
185. Ma, Y., et al., *Phytovirome Analysis of Wild Plant Populations: Comparison of Double-Stranded RNA and Virion-Associated Nucleic Acid Metagenomic Approaches.* J Virol, 2019. **94**(1).
186. Ma, Y., et al., *Metagenomic analysis of virome cross-talk between cultivated Solanum lycopersicum and wild Solanum nigrum.* Virology, 2020. **540**: p. 38-44.
187. Mahmood, A., et al., *Viral Metagenomics Revealed a Novel Cardiovirus in Feces of Wild Rats.* Intervirology, 2019. **62**(1): p. 45-50.

188. Maia, L.M.S., et al., *Novel Viruses in Mosquitoes from Brazilian Pantanal.* Viruses, 2019. **11**(10).
189. Masuda, T., et al., *Identification of novel bovine group A rotavirus G15P[14] strain from epizootic diarrhea of adult cows by de novo sequencing using a next-generation sequencer.* Vet Microbiol, 2014. **171**(1-2): p. 66-73.
190. Masuda, T., et al., *Whole genome analysis of a novel picornavirus related to the Enterovirus/Sapelovirus supergroup from porcine feces in Japan.* Virus Res, 2018. **257**: p. 68-73.
191. Mihalov-Kovács, E., et al., *Candidate new rotavirus species in sheltered dogs, Hungary.* Emerg Infect Dis, 2015. **21**(4): p. 660-3.
192. Minot, S., et al., *Rapid evolution of the human gut virome.* Proc Natl Acad Sci U S A, 2013. **110**(30): p. 12450-5.
193. Miranda, J.A., et al., *RNA viruses as major contributors to Antarctic virioplankton.* Environ Microbiol, 2016. **18**(11): p. 3714-3727.
194. Mitchell, A.B., et al., *High Resolution Metatranscriptomic Characterization of the Pulmonary RNA Virome After Lung Transplantation.* Transplantation, 2021.
195. Mizuno, C.M., et al., *Expanding the marine virosphere using metagenomics.* PLoS Genet, 2013. **9**(12): p. e1003987.
196. Mizuno, C.M., et al., *Genomes of Abundant and Widespread Viruses from the Deep Ocean.* mBio, 2016. **7**(4).
197. Mollerup, S., et al., *Cutavirus in Cutaneous Malignant Melanoma.* Emerg Infect Dis, 2017. **23**(2): p. 363-365.
198. Molnár, J., et al., *Identification of a novel archaea virus, detected in hydrocarbon polluted Hungarian and Canadian samples.* PLoS One, 2020. **15**(4): p. e0231864.
199. Moutailler, S., et al., *Diversity of viruses in Ixodes ricinus, and characterization of a neurotropic strain of Eyach virus.* New Microbes New Infect, 2016. **11**: p. 71-81.
200. Mozar, M. and J.M. Claverie, *Expanding the Mimiviridae family using asparagine synthase as a sequence bait.* Virology, 2014. **466-467**: p. 112-22.
201. Mukai, Y., et al., *Identification of a distinct lineage of aviadenovirus from crane feces.* Virus Genes, 2019. **55**(6): p. 815-824.
202. Mushegian, A., A. Shipunov, and S.F. Elena, *Changes in the composition of the RNA virome mark evolutionary transitions in green plants.* BMC Biol, 2016. **14**: p. 68.
203. Nagai, M., et al., *Identification and complete genome analysis of a novel bovine picornavirus in Japan.* Virus Res, 2015. **210**: p. 205-12.
204. Naoi, Y., et al., *Characterization and phylogenetic analysis of a novel picornavirus from swine feces in Japan.* Arch Virol, 2016. **161**(6): p. 1685-90.
205. Nebbak, A., et al., *Virome Diversity among Mosquito Populations in a Sub-Urban Region of Marseille, France.* Viruses, 2021. **13**(5).
206. Nemchinov, L.G., et al., *Characterization of alfalfa virus F, a new member of the genus Marafivirus.* PLoS One, 2018. **13**(9): p. e0203477.
207. Nery, F.M.B., et al., *Molecular Characterization of Hovenia Dulcis-Associated Virus 1 (HDaV1) and 2 (HDaV2): New Tentative Species within the Order Picornavirales.* Viruses, 2020. **12**(9).
208. Nishimura, Y., et al., *Environmental Viral Genomes Shed New Light on Virus-Host Interactions in the Ocean.* mSphere, 2017. **2**(2).
209. Nouri, S., et al., *Diverse Array of New Viral Sequences Identified in Worldwide Populations of the Asian Citrus Psyllid (Diaphorina citri) Using Viral Metagenomics.* J Virol, 2015. **90**(5): p. 2434-45.
210. Oba, M., et al., *Metagenomic identification and sequence analysis of a Teschovirus A-related virus in porcine feces in Japan, 2014-2016.* Infect Genet Evol, 2018. **66**: p. 210-216.
211. Oh, S., D. Yoo, and W.T. Liu, *Metagenomics Reveals a Novel Virophage Population in a Tibetan Mountain Lake.* Microbes Environ, 2016. **31**(2): p. 173-7.
212. Öhlund, P., et al., *Viromics Reveal a Number of Novel RNA Viruses in Swedish Mosquitoes.* Viruses, 2019. **11**(11).
213. Öncü, C., et al., *West Nile virus, Anopheles flavivirus, a novel flavivirus as well as Merida-like rhabdovirus Turkey in field-collected mosquitoes from Thrace and Anatolia.* Infect Genet Evol, 2018. **57**: p. 36-45.
214. Orton, J.P., et al., *Virus Discovery in Desert Tortoise Fecal Samples: Novel Circular Single-Stranded DNA Viruses.* Viruses, 2020. **12**(2).
215. Chen, P., et al., *Revealing the full biosphere structure and versatile metabolic functions in the deepest ocean sediment of the Challenger Deep.* Genome Biol, 2021. **22**(1): p. 207.
216. Palanga, E., et al., *Metagenomic-Based Screening and Molecular Characterization of Cowpea-Infecting Viruses in Burkina Faso.* PLoS One, 2016. **11**(10): p. e0165188.
217. Pan, S., et al., *Identification of a Torque Teno Mini Virus (TTMV) in Hodgkin's Lymphoma Patients.* Front Microbiol, 2018. **9**: p. 1680.
218. Pankovics, P., et al., *Detection of a mammalian-like astrovirus in bird, European roller (Coracias garrulus).* Infect Genet Evol, 2015. **34**: p. 114-21.

219. Pankovics, P., et al., *A novel passerivirus (family Picornaviridae) in an outbreak of enteritis with high mortality in estrildid finches (Uraeginthus sp.).* Arch Virol, 2018. **163**(4): p. 1063-1071.
220. Pearson, V.M., S.B. Caudle, and D.R. Rokyta, *Viral recombination blurs taxonomic lines: examination of single-stranded DNA viruses in a wastewater treatment plant.* PeerJ, 2016. **4**: p. e2585.
221. Pérez-Sautu, U., et al., *Novel viruses in hard ticks collected in the Republic of Korea unveiled by metagenomic high-throughput sequencing analysis.* Ticks Tick Borne Dis, 2021. **12**(6): p. 101820.
222. Petersen, A., et al., *Field samplings of Ixodes ricinus ticks from a tick-borne encephalitis virus micro-focus in Northern Zealand, Denmark.* Ticks Tick Borne Dis, 2019. **10**(5): p. 1028-1032.
223. Pfaff, F., et al., *A novel astrovirus associated with encephalitis and ganglionitis in domestic sheep.* Transbound Emerg Dis, 2017. **64**(3): p. 677-682.
224. Pfreundt, U., et al., *Genome of a giant bacteriophage from a decaying Trichodesmium bloom.* Mar Genomics, 2017. **33**: p. 21-25.
225. Phan, T.G., et al., *Sera of Peruvians with fever of unknown origins include viral nucleic acids from non-vertebrate hosts.* Virus Genes, 2018. **54**(1): p. 33-40.
226. Phan, T.G., et al., *The fecal virome of South and Central American children with diarrhea includes small circular DNA viral genomes of unknown origin.* Arch Virol, 2016. **161**(4): p. 959-66.
227. Phan, T.G., et al., *A new protoparvovirus in human fecal samples and cutaneous T cell lymphomas (mycosis fungoides).* Virology, 2016. **496**: p. 299-305.
228. Phan, T.G., et al., *Detection of a novel circovirus PCV3 in pigs with cardiac and multi-systemic inflammation.* Virol J, 2016. **13**(1): p. 184.
229. Phan, T.G., et al., *Cyclovirus in nasopharyngeal aspirates of Chilean children with respiratory infections.* J Gen Virol, 2014. **95**(Pt 4): p. 922-927.
230. Phan, T.G., et al., *New astrovirus in human feces from Burkina Faso.* J Clin Virol, 2014. **60**(2): p. 161-4.
231. Plyusnin, I., et al., *Novel NGS pipeline for virus discovery from a wide spectrum of hosts and sample types.* Virus Evol, 2020. **6**(2): p. veaa091.
232. Popgeorgiev, N., et al., *Marseillevirus-like virus recovered from blood donated by asymptomatic humans.* J Infect Dis, 2013. **208**(7): p. 1042-50.
233. Porter, A.F., et al., *Novel hepaci- and pegi-like viruses in native Australian wildlife and non-human primates.* Virus Evol, 2020. **6**(2): p. veaa064.
234. Pyke, A.T., et al., *Uncovering the genetic diversity within the Aedes notoscriptus virome and isolation of new viruses from this highly urbanised and invasive mosquito.* Virus Evol, 2021. **7**(2): p. veab082.
235. Liu, Q., et al., *Viral metagenomics revealed diverse CRESS-DNA virus genomes in faeces of forest musk deer.* Virol J, 2020. **17**(1): p. 61.
236. Qi, D., et al., *A novel polyomavirus from the nasal cavity of a giant panda (Ailuropoda melanoleuca).* Virol J, 2017. **14**(1): p. 207.
237. Qiu, L., et al., *Characterization of a new member of Iridoviridae, Shrimp hemocyte iridescent virus (SHIV), found in white leg shrimp (Litopenaeus vannamei).* Sci Rep, 2017. **7**(1): p. 11834.
238. Quaiser, A., et al., *Diversity and comparative genomics of Microviridae in Sphagnum- dominated peatlands.* Front Microbiol, 2015. **6**: p. 375.
239. Quaiser, A., et al., *Diversity and comparative genomics of chimeric viruses in Sphagnum-dominated peatlands.* Virus Evol, 2016. **2**(2): p. vew025.
240. Ramírez, A.L., et al., *Metagenomic Analysis of the Virome of Mosquito Excreta.* mSphere, 2020. **5**(5).
241. Ramírez-Martínez, M.M., et al., *Bat Flies of the Family Streblidae (Diptera: Hippoboscoidea) Host Relatives of Medically and Agriculturally Important "Bat-Associated" Viruses.* Viruses, 2021. **13**(5).
242. Rastrojo, A., et al., *A New Putative Caulimoviridae Genus Discovered through Air Metagenomics.* Microbiol Resour Announc, 2018. **7**(14).
243. Remnant, E.J., et al., *A Diverse Range of Novel RNA Viruses in Geographically Distinct Honey Bee Populations.* J Virol, 2017. **91**(16).
244. Reuter, G., et al., *Novel seadornavirus (family Reoviridae) related to Banna virus in Europe.* Arch Virol, 2013. **158**(10): p. 2163-7.
245. Reuter, G., et al., *Novel dicistrovirus from bat guano.* Arch Virol, 2014. **159**(12): p. 3453-6.
246. Reuter, G., et al., *Detection of a novel RNA virus with hepatitis E virus-like non-structural genome organization in amphibian, agile frog (Rana dalmatina) tadpoles.* Infect Genet Evol, 2018. **65**: p. 112-116.

247. Reuter, G., et al., *Detection and complete genome characterization of a novel RNA virus related to members of the Hepe-Virga clade in bird species, hoopoe (Upupa epops).* Infect Genet Evol, 2020. **81**: p. 104236.
248. Reuter, G., et al., *A highly divergent picornavirus in an amphibian, the smooth newt (Lissotriton vulgaris).* J Gen Virol, 2015. **96**(9): p. 2607-2613.
249. Reuter, G., et al., *A novel posavirus-related single-stranded RNA virus from fish (Cyprinus carpio).* Arch Virol, 2015. **160**(2): p. 565-8.
250. Richard, J.C., et al., *Mass mortality in freshwater mussels (Actinonaias pectorosa) in the Clinch River, USA, linked to a novel densovirus.* Sci Rep, 2020. **10**(1): p. 14498.
251. Richet, C., et al., *Novel circular DNA viruses associated with Apiaceae and Poaceae from South Africa and New Zealand.* Arch Virol, 2019. **164**(1): p. 237-242.
252. Rodriguez, C., et al., *Fatal Encephalitis Caused by Cristoli Virus, an Emerging Orthobunyavirus, France.* Emerg Infect Dis, 2020. **26**(6): p. 1287-1290.
253. Roediger, B., et al., *An Atypical Parvovirus Drives Chronic Tubulointerstitial Nephropathy and Kidney Fibrosis.* Cell, 2018. **175**(2): p. 530-543.e24.
254. Roossinck, M.J., et al., *Ecogenomics: using massively parallel pyrosequencing to understand virus ecology.* Mol Ecol, 2010. **19 Suppl 1**: p. 81-8.
255. Rosario, K., S. Duffy, and M. Breitbart, *Diverse circovirus-like genome architectures revealed by environmental metagenomics.* J Gen Virol, 2009. **90**(Pt 10): p. 2418-2424.
256. Rosario, K., et al., *Dragonfly cyclovirus, a novel single-stranded DNA virus discovered in dragonflies (Odonata: Anisoptera).* J Gen Virol, 2011. **92**(Pt 6): p. 1302-1308.
257. Rosario, K., et al., *Discovery of a novel mastrevirus and alphasatellite-like circular DNA in dragonflies (Epiprocta) from Puerto Rico.* Virus Res, 2013. **171**(1): p. 231-7.
258. Rosario, K., et al., *Novel circular single-stranded DNA viruses identified in marine invertebrates reveal high sequence diversity and consistent predicted intrinsic disorder patterns within putative structural proteins.* Front Microbiol, 2015. **6**: p. 696.
259. Rosario, K., et al., *Vector-Enabled Metagenomic (VEM) Surveys Using Whiteflies (Aleyrodidae) Reveal Novel Begomovirus Species in the New and Old Worlds.* Viruses, 2015. **7**(10): p. 5553-70.
260. Roux, S., et al., *Assessing the diversity and specificity of two freshwater viral communities through metagenomics.* PLoS One, 2012. **7**(3): p. e33641.
261. Roux, S., et al., *Ecology and evolution of viruses infecting uncultivated SUP05 bacteria as revealed by single-cell- and meta-genomics.* Elife, 2014. **3**: p. e03125.
262. Roux, S., et al., *Ecogenomics of virophages and their giant virus hosts assessed through time series metagenomics.* Nat Commun, 2017. **8**(1): p. 858.
263. Ruiz-Padilla, A., et al., *Novel Mycoviruses Discovered in the Mycovirome of a Necrotrophic Fungus.* mBio, 2021. **12**(3).
264. S, I.S., et al., *High-Quality Resolution of the Outbreak-Related Zika Virus Genome and Discovery of New Viruses Using Ion Torrent-Based Metatranscriptomics.* Viruses, 2020. **12**(7).
265. Li, S.K., et al., *Detection and identification of plasma bacterial and viral elements in HIV/AIDS patients in comparison to healthy adults.* Clin Microbiol Infect, 2012. **18**(11): p. 1126-33.
266. Yang, S., et al., *A novel rodent Chapparvovirus in feces of wild rats.* Virol J, 2016. **13**: p. 133.
267. Yang, S., et al., *Bufavirus Protoparvovirus in feces of wild rats in China.* Virus Genes, 2016. **52**(1): p. 130-3.
268. Yang, S., et al., *A novel bocavirus from domestic mink, China.* Virus Genes, 2016. **52**(6): p. 887-890.
269. Saad, N., et al., *Discovery of Known and Novel Viruses in Wild and Cultivated Blueberry in Florida through Viral Metagenomic Approaches.* Viruses, 2021. **13**(6).
270. Sadeghi, M., et al., *Virome of > 12 thousand Culex mosquitoes from throughout California.* Virology, 2018. **523**: p. 74-88.
271. Sadeghi, M., et al., *Virome of US bovine calf serum.* Biologicals, 2017. **46**: p. 64-67.
272. Sadeghi, M., et al., *Genomes of viral isolates derived from different mosquitos species.* Virus Res, 2017. **242**: p. 49-57.
273. Sanborn, M.A., et al., *Metagenomic Analysis Reveals Three Novel and Prevalent Mosquito Viruses from a Single Pool of Aedes vexans nipponii Collected in the Republic of Korea.* Viruses, 2019. **11**(3).
274. Sano, K., et al., *Identification of further diversity among posaviruses.* Arch Virol, 2016. **161**(12): p. 3541-3548.
275. Santos, P.D., et al., *In action-an early warning system for the detection of unexpected or novel pathogens.* Virus Evol, 2021. **7**(2): p. veab085.

276. Sasaki, M., et al., *Metagenomic analysis of the shrew enteric virome reveals novel viruses related to human stool-associated viruses.* J Gen Virol, 2015. **96**(Pt 2): p. 440-452.
277. Scheets, K., *Infectious transcripts of an asymptomatic panicovirus identified from a metagenomic survey.* Virus Res, 2013. **176**(1-2): p. 161-8.
278. Schlottau, K., et al., *Detection of a Novel Bovine Astrovirus in a Cow with Encephalitis.* Transbound Emerg Dis, 2016. **63**(3): p. 253-9.
279. Schoonvaere, K., et al., *Unbiased RNA Shotgun Metagenomics in Social and Solitary Wild Bees Detects Associations with Eukaryote Parasites and New Viruses.* PLoS One, 2016. **11**(12): p. e0168456.
280. Schulz, F., et al., *Hidden diversity of soil giant viruses.* Nat Commun, 2018. **9**(1): p. 4881.
281. Schürch, A.C., et al., *Metagenomic survey for viruses in Western Arctic caribou, Alaska, through iterative assembly of taxonomic units.* PLoS One, 2014. **9**(8): p. e105227.
282. Shahhosseini, N., et al., *Detection and characterization of a novel rhabdovirus in Aedes cantans mosquitoes and evidence for a mosquito-associated new genus in the family Rhabdoviridae.* Infect Genet Evol, 2017. **55**: p. 260-268.
283. Shan, T., et al., *The fecal virome of pigs on a high-density farm.* J Virol, 2011. **85**(22): p. 11697-708.
284. Shang, P., et al., *A Naturally Occurring Recombinant Enterovirus Expresses a Torovirus Deubiquitinase.* J Virol, 2017. **91**(14).
285. Shi, C., et al., *Stable distinct core eukaryotic viromes in different mosquito species from Guadeloupe, using single mosquito viral metagenomics.* Microbiome, 2019. **7**(1): p. 121.
286. Šimić, I., et al., *Novel Circo-Like Virus Detected in a Croatian Bat Population.* Microbiol Resour Announc, 2019. **8**(16).
287. Šimić, I., et al., *Viral Metagenomic Profiling of Croatian Bat Population Reveals Sample and Habitat Dependent Diversity.* Viruses, 2020. **12**(8).
288. Simsek, C., et al., *At Least Seven Distinct Rotavirus Genotype Constellations in Bats with Evidence of Reassortment and Zoonotic Transmissions.* mBio, 2021. **12**(1).
289. Siqueira, J.D., et al., *ENDEMIC INFECTION OF STRANDED SOUTHERN SEA OTTERS (ENHYDRA LUTRIS NEREIS) WITH NOVEL PARVOVIRUS, POLYOMAVIRUS, AND ADENOVIRUS.* J Wildl Dis, 2017. **53**(3): p. 532-542.
290. Smits, S.L., et al., *New viruses in idiopathic human diarrhea cases, the Netherlands.* Emerg Infect Dis, 2014. **20**(7): p. 1218-22.
291. Smits, S.L., et al., *Metagenomic analysis of the ferret fecal viral flora.* PLoS One, 2013. **8**(8): p. e71595.
292. Smits, S.L., et al., *Novel cyclovirus in human cerebrospinal fluid, Malawi, 2010-2011.* Emerg Infect Dis, 2013. **19**(9): p. 1511-3.
293. Song, X., et al., *An emerging orthopneumovirus detected from dogs with canine infectious respiratory disease in China.* Transbound Emerg Dis, 2021. **68**(6): p. 3217-3221.
294. Souza, W.M., et al., *Viral diversity of Rhipicephalus microplus parasitizing cattle in southern Brazil.* Sci Rep, 2018. **8**(1): p. 16315.
295. Sun, M., et al., *Uncultivated Viral Populations Dominate Estuarine Viromes on the Spatiotemporal Scale.* mSystems, 2021. **6**(2).
296. Susi, H., et al., *Genome sequences of a capulavirus infecting Plantago lanceolata in the Åland archipelago of Finland.* Arch Virol, 2017. **162**(7): p. 2041-2045.
297. Susi, H., et al., *Diverse and variable virus communities in wild plant populations revealed by metagenomic tools.* PeerJ, 2019. **7**: p. e6140.
298. Suzuki, Y., et al., *Long-read metagenomic exploration of extrachromosomal mobile genetic elements in the human gut.* Microbiome, 2019. **7**(1): p. 119.
299. Ng, T.F.F., et al., *Metagenomic identification of a novel anellovirus in Pacific harbor seal (Phoca vitulina richardsii) lung samples and its detection in samples from multiple years.* J Gen Virol, 2011. **92**(Pt 6): p. 1318-1323.
300. Ng, T.F.F., et al., *Two new species of betatorqueviruses identified in a human melanoma that metastasized to the brain.* Oncotarget, 2017. **8**(62): p. 105800-105808.
301. Ng, T.F., et al., *Discovery of a novel single-stranded DNA virus from a sea turtle fibropapilloma by using viral metagenomics.* J Virol, 2009. **83**(6): p. 2500-9.
302. Ng, T.F., et al., *High variety of known and new RNA and DNA viruses of diverse origins in untreated sewage.* J Virol, 2012. **86**(22): p. 12161-75.
303. Ng, T.F., et al., *Feline fecal virome reveals novel and prevalent enteric viruses.* Vet Microbiol, 2014. **171**(1-2): p. 102-11.
304. Ng, T.F., et al., *Oral papillomatosis caused by Enhydra lutris papillomavirus 1 (ElPV-1) in southern sea otters (Enhydra lutris nereis) in California, USA.* J Wildl Dis, 2015. **51**(2): p. 446-53.

305. Ng, T.F., et al., *Metagenomic identification of a nodavirus and a circular ssDNA virus in semi-purified viral nucleic acids from the hepatopancreas of healthy Farfantepenaeus duorarum shrimp.* Dis Aquat Organ, 2013. **105**(3): p. 237-42.
306. Ng, T.F., et al., *Broad surveys of DNA viral diversity obtained through viral metagenomics of mosquitoes.* PLoS One, 2011. **6**(6): p. e20579.
307. Ng, T.F., et al., *Identification of an astrovirus commonly infecting laboratory mice in the US and Japan.* PLoS One, 2013. **8**(6): p. e66937.
308. Ng, T.F., et al., *Exploring the diversity of plant DNA viruses and their satellites using vector-enabled metagenomics on whiteflies.* PLoS One, 2011. **6**(4): p. e19050.
309. Li, T., et al., *Metagenomic Next-Generation Sequencing of the 2014 Ebola Virus Disease Outbreak in the Democratic Republic of the Congo.* J Clin Microbiol, 2019. **57**(9).
310. Zhao, T., et al., *Comparison of Viromes in Ticks from Different Domestic Animals in China.* Virol Sin, 2020. **35**(4): p. 398-406.
311. Tahmasebi, R., et al., *Genomic Analyses of Potential Novel Recombinant Human Adenovirus C in Brazil.* Viruses, 2020. **12**(5).
312. Tamaki, H., et al., *Metagenomic analysis of DNA viruses in a wastewater treatment plant in tropical climate.* Environ Microbiol, 2012. **14**(2): p. 441-52.
313. Tangudu, C.S., et al., *Skunk River virus, a novel orbivirus isolated from Aedes trivittatus in the United States.* J Gen Virol, 2019. **100**(2): p. 295-300.
314. Temmam, S., et al., *Characterization of Viral Communities of Biting Midges and Identification of Novel Thogotovirus Species and Rhabdovirus Genus.* Viruses, 2016. **8**(3): p. 77.
315. Thannesberger, J., et al., *Viral metagenomics reveals the presence of novel Zika virus variants in Aedes mosquitoes from Barbados.* Parasit Vectors, 2021. **14**(1): p. 343.
316. Theuns, S., et al., *Characterization of a genetically heterogeneous porcine rotavirus C, and other viruses present in the fecal virome of a non-diarrheic Belgian piglet.* Infect Genet Evol, 2016. **43**: p. 135-45.
317. Thi Kha Tu, N., et al., *The Virome of Acute Respiratory Diseases in Individuals at Risk of Zoonotic Infections.* Viruses, 2020. **12**(9).
318. Tisza, M.J., et al., *Discovery of several thousand highly diverse circular DNA viruses.* Elife, 2020. **9**.
319. Truchado, D.A., et al., *A Novel and Divergent Gyrovirus with Unusual Genomic Features Detected in Wild Passerine Birds from a Remote Rainforest in French Guiana.* Viruses, 2019. **11**(12).
320. Truchado, D.A., et al., *Comparative Metagenomics of Palearctic and Neotropical Avian Cloacal Viromes Reveal Geographic Bias in Virus Discovery.* Microorganisms, 2020. **8**(12).
321. Tse, H., et al., *Identification of a novel bat papillomavirus by metagenomics.* PLoS One, 2012. **7**(8): p. e43986.
322. Tsuchiaka, S., et al., *Identification of a novel bovine enterovirus possessing highly divergent amino acid sequences in capsid protein.* BMC Microbiol, 2017. **17**(1): p. 18.
323. Tsuchiaka, S., et al., *Genetic diversity and recombination of enterovirus G strains in Japanese pigs: High prevalence of strains carrying a papain-like cysteine protease sequence in the enterovirus G population.* PLoS One, 2018. **13**(1): p. e0190819.
324. Ullah, K., et al., *Detection and molecular characterization of picobirnaviruses in the wild birds: Identification of a novel picobirnavirus possessing yeast mitochondrial genetic code.* Virus Res, 2021: p. 198624.
325. Urayama, S.I., et al., *Unveiling the RNA virosphere associated with marine microorganisms.* Mol Ecol Resour, 2018. **18**(6): p. 1444-1455.
326. Valles, S.M. and Y. Hashimoto, *Isolation and characterization of Solenopsis invicta virus 3, a new positive-strand RNA virus infecting the red imported fire ant, Solenopsis invicta.* Virology, 2009. **388**(2): p. 354-61.
327. Valles, S.M. and A.R. Rivers, *Nine new RNA viruses associated with the fire ant Solenopsis invicta from its native range.* Virus Genes, 2019. **55**(3): p. 368-380.
328. Valles, S.M., et al., *Metatranscriptomics and pyrosequencing facilitate discovery of potential viral natural enemies of the invasive Caribbean crazy ant, Nylanderia pubens.* PLoS One, 2012. **7**(2): p. e31828.
329. Valles, S.M., S.D. Porter, and L.A. Calcaterra, *Prospecting for viral natural enemies of the fire ant Solenopsis invicta in Argentina.* PLoS One, 2018. **13**(2): p. e0192377.

330. van Beurden, S.J., et al., *A novel cetacean adenovirus in stranded harbour porpoises from the North Sea: detection and molecular characterization.* Arch Virol, 2017. **162**(7): p. 2035-2040.
331. Vandegrift, K.J., et al., *Presence of Segmented Flavivirus Infections in North America.* Emerg Infect Dis, 2020. **26**(8): p. 1810-1817.
332. Vanmechelen, B., et al., *Exploration of the Ixodes ricinus virosphere unveils an extensive virus diversity including novel coltiviruses and other reoviruses.* Virus Evol, 2021. **7**(2): p. veab066.
333. Vibin, J., et al., *Metagenomic characterisation of additional and novel avian viruses from Australian wild ducks.* Sci Rep, 2020. **10**(1): p. 22284.
334. Vibin, J., et al., *Metagenomic characterisation of avian parvoviruses and picornaviruses from Australian wild ducks.* Sci Rep, 2020. **10**(1): p. 12800.
335. Victoria, J.G., et al., *Rapid identification of known and new RNA viruses from animal tissues.* PLoS Pathog, 2008. **4**(9): p. e1000163.
336. Villamor, J., et al., *Characterization of ecologically diverse viruses infecting co-occurring strains of cosmopolitan hyperhalophilic Bacteroidetes.* Isme j, 2018. **12**(2): p. 424-437.
337. Villanova, F., et al., *New Variants of Squash Mosaic Viruses Detected in Human Fecal Samples.* Microorganisms, 2021. **9**(7).
338. Voorhies, A.A., et al., *Ecological and genetic interactions between cyanobacteria and viruses in a low-oxygen mat community inferred through metagenomics and metatranscriptomics.* Environ Microbiol, 2016. **18**(2): p. 358-71.
339. Zhang, W., et al., *Identification and genomic characterization of a novel species of feline anellovirus.* Virol J, 2016. **13**(1): p. 146.
340. Zhang, W., et al., *Virome comparisons in wild-diseased and healthy captive giant pandas.* Microbiome, 2017. **5**(1): p. 90.
341. Zhang, W., et al., *Characterization and genomic analysis of the first Oceanospirillum phage, vB_OliS_GJ44, representing a novel siphoviral cluster.* BMC Genomics, 2021. **22**(1): p. 675.
342. Zhang, W., et al., *What is for dinner? Viral metagenomics of US store bought beef, pork, and chicken.* Virology, 2014. **468-470**: p. 303-310.
343. Zhang, W., et al., *Faecal virome of cats in an animal shelter.* J Gen Virol, 2014. **95**(Pt 11): p. 2553-2564.
344. Wamonje, F.O., et al., *Viral metagenomics of aphids present in bean and maize plots on mixed-use farms in Kenya reveals the presence of three dicistroviruses including a novel Big Sioux River virus-like dicistrovirus.* Virol J, 2017. **14**(1): p. 188.
345. Weber, M.N., et al., *Characterization of dog serum virome from Northeastern Brazil.* Virology, 2018. **525**: p. 192-199.
346. Webster, C.L., et al., *Twenty-Five New Viruses Associated with the Drosophilidae (Diptera).* Evol Bioinform Online, 2016. **12**(Suppl 2): p. 13-25.
347. Williams, S.H., et al., *The Diversity and Distribution of Viruses Associated with Culex annulirostris Mosquitoes from the Kimberley Region of Western Australia.* Viruses, 2020. **12**(7).
348. Wu, S., et al., *Diverse and unique viruses discovered in the surface water of the East China Sea.* BMC Genomics, 2020. **21**(1): p. 441.
349. Wüthrich, D., et al., *Exploring the virome of cattle with non-suppurative encephalitis of unknown etiology by metagenomics.* Virology, 2016. **493**: p. 22-30.
350. Wang, X.C., et al., *Viral metagenomics reveals diverse anelloviruses in bone marrow specimens from hematologic patients.* J Clin Virol, 2020. **132**: p. 104643.
351. Chen, X., et al., *A novel astrovirus species in the gut of yaks with diarrhoea in the Qinghai–Tibetan Plateau, 2013.* J Gen Virol, 2015. **96**(12): p. 3672-3680.
352. Liu, X., et al., *Genomic and transcriptional analyses of novel parvoviruses identified from dead peafowl.* Virology, 2020. **539**: p. 80-91.
353. Chen, X.U., et al., *Identification and Characterization of a Novel Recombinant Porcine Astrovirus from Pigs in Anhui, China.* Pol J Microbiol, 2020. **69**(4): p. 471-478.
354. Wang, X., et al., *An enterovirus from a captive primate in China.* Springerplus, 2016. **5**(1): p. 1281.
355. Xia, H., et al., *Comparative Metagenomic Profiling of Viromes Associated with Four Common Mosquito Species in China.* Virol Sin, 2018. **33**(1): p. 59-66.
356. Kim, Y., et al., *Metagenomic Investigation of Viral Communities in Ballast Water.* Environ Sci Technol, 2015. **49**(14): p. 8396-407.
357. Li, Y., et al., *Virome of Bat Guano from Nine Northern California Roosts.* J Virol, 2021. **95**(3).
358. Li, Y., et al., *Astrovirus Outbreak in an Animal Shelter Associated With Feline Vomiting.* Front Vet Sci, 2021. **8**: p. 628082.
359. Li, Y., et al., *Virome of a Feline Outbreak of Diarrhea and Vomiting Includes Bocaviruses and a Novel Chapparvovirus.* Viruses, 2020. **12**(5).
360. Li, Y., et al., *Identification and genome characterization of a novel picornavirus from ducks in China.* Arch Virol, 2020. **165**(9): p. 2087-2089.

361. Wang, Y., et al., *A novel cardiovirus in wild rats.* Virol J, 2018. **15**(1): p. 58.
362. Wang, Y., et al., *The fecal virome of red-crowned cranes.* Arch Virol, 2019. **164**(1): p. 3-16.
363. Xu, Y., et al., *A novel Enterovirus 96 circulating in China causes hand, foot, and mouth disease.* Virus Genes, 2017. **53**(3): p. 352-356.
364. Zhang, Y., et al., *A novel species of torque teno mini virus (TTMV) in gingival tissue from chronic periodontitis patients.* Sci Rep, 2016. **6**: p. 26739.
365. Zhang, Y., et al., *Detection of a new species of torque teno mini virus from the gingival epithelium of patients with periodontitis.* Virus Genes, 2017. **53**(6): p. 823-830.
366. Zhang, Y., et al., *A novel phage from periodontal pockets associated with chronic periodontitis.* Virus Genes, 2019. **55**(3): p. 381-393.
367. Yinda, C.K., et al., *Novel highly divergent reassortant bat rotaviruses in Cameroon, without evidence of zoonosis.* Sci Rep, 2016. **6**: p. 34209.
368. Yinda, C.K., et al., *Cameroonian fruit bats harbor divergent viruses, including rotavirus H, bastroviruses, and picobirnaviruses using an alternative genetic code.* Virus Evol, 2018. **4**(1): p. vey008.
369. Yinda, C.K., et al., *Novel highly divergent sapoviruses detected by metagenomics analysis in straw-colored fruit bats in Cameroon.* Emerg Microbes Infect, 2017. **6**(5): p. e38.
370. Yinda, C.K., et al., *Highly diverse population of Picornaviridae and other members of the Picornavirales, in Cameroonian fruit bats.* BMC Genomics, 2017. **18**(1): p. 249.
371. Yutin, N., V.V. Kapitonov, and E.V. Koonin, *A new family of hybrid virophages from an animal gut metagenome.* Biol Direct, 2015. **10**: p. 19.
372. Yang, Z., et al., *Virome analysis of ticks in a forest region of Liaoning, China: characterization of a novel hepe-like virus sequence.* Virol J, 2021. **18**(1): p. 163.
373. Zakham, F., et al., *Viral RNA Metagenomics of Hyalomma Ticks Collected from Dromedary Camels in Makkah Province, Saudi Arabia.* Viruses, 2021. **13**(7).
374. Zaragoza-Solas, A., F. Rodriguez-Valera, and M. López-Pérez, *Metagenome Mining Reveals Hidden Genomic Diversity of Pelagimyophages in Aquatic Environments.* mSystems, 2020. **5**(1).
375. Zhan, Y., et al., *A novel roseobacter phage possesses features of podoviruses, siphoviruses, prophages and gene transfer agents.* Sci Rep, 2016. **6**: p. 30372.
376. Zhou, H., et al., *Identification and Genome Characterization of the First Sicinivirus Isolate from Chickens in Mainland China by Using Viral Metagenomics.* PLoS One, 2015. **10**(10): p. e0139668.
377. Zhu, Q., et al., *Visualization-assisted binning of metagenome assemblies reveals potential new pathogenic profiles in idiopathic travelers' diarrhea.* Microbiome, 2018. **6**(1): p. 201.
378. Zsak, L., et al., *The complete genome sequence and genetic analysis of ΦCA82 a novel uncultured microphage from the turkey gastrointestinal system.* Virol J, 2011. **8**: p. 331.
379. Zschach, H., et al., *What Can We Learn from a Metagenomic Analysis of a Georgian Bacteriophage Cocktail?* Viruses, 2015. **7**(12): p. 6570-89.
