# Supplementary figures and images for: Novel Virus Identification through Metagenomics: A Systematic Review
Source: Life (Basel). 2022 Dec 7;12(12):2048. doi: 10.3390/life12122048 (PMC9784588; doi:10.3390/life12122048)

Figure S1

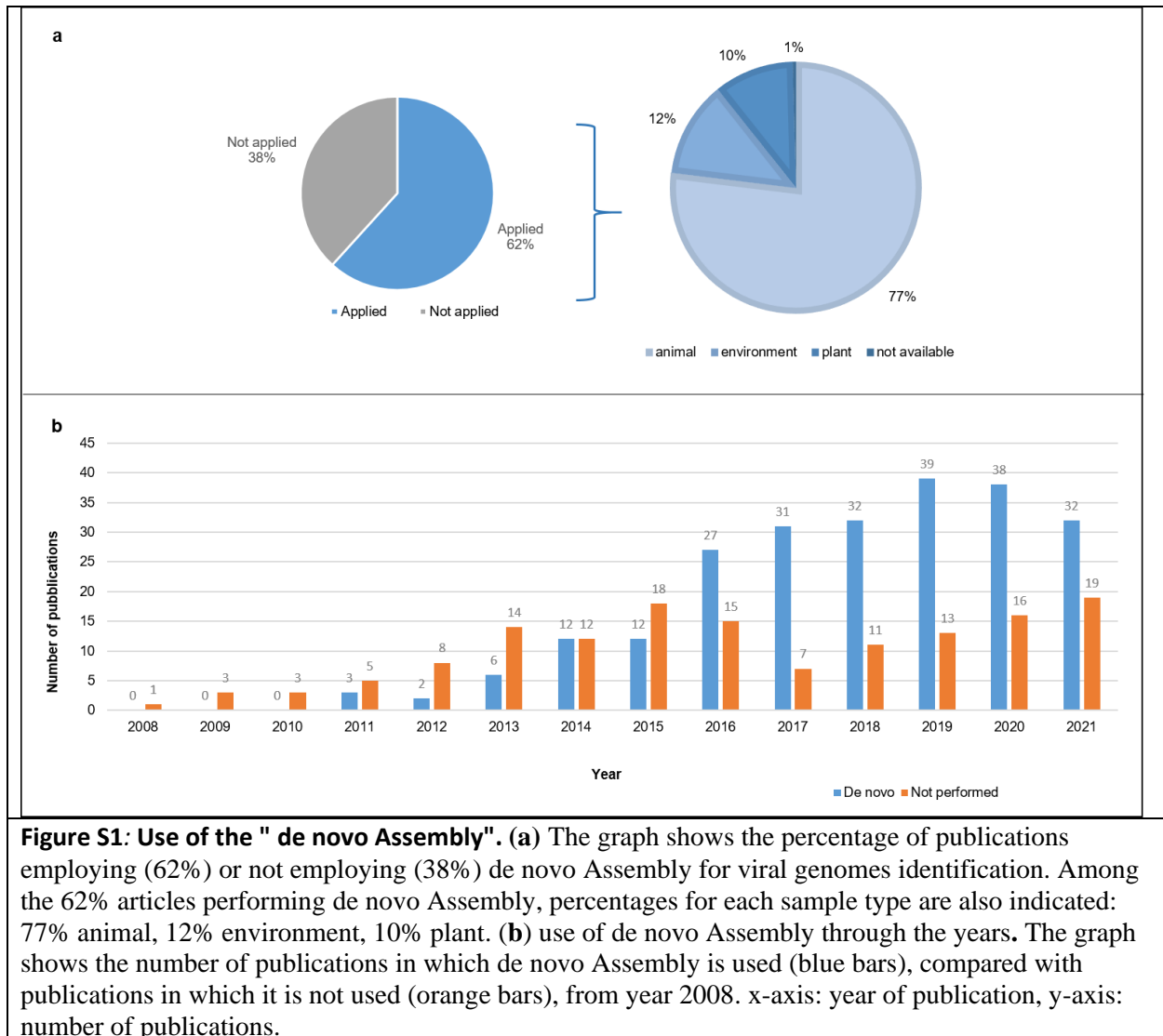

Supplement: Supplementary file 1 [file life-12-02048-s001.zip › Bassi_Figure S1.pdf]
